# Extensive genetic diversity of *Plasmodium vivax dbp*-II in Rio de Janeiro Atlantic Forest and Brazilian Amazon Basin: evidence of positive selection

**DOI:** 10.1186/s12936-020-03159-y

**Published:** 2020-02-19

**Authors:** Natália Ketrin Almeida-de-Oliveira, Lidiane Lima-Cury, Rebecca de Abreu-Fernandes, Aline de Rosa Lavigne, Anielle de Pina-Costa, Daiana de Souza Perce-da-Silva, Marcos Catanho, Patrícia Brasil, Cláudio Tadeu Daniel-Ribeiro, Maria de Fátima Ferreira-da-Cruz

**Affiliations:** 1 Laboratório de Pesquisas em Malária, Instituto Oswaldo Cruz, Fundação Oswaldo Cruz (Fiocruz), Avenida Brasil 4365, Manguinhos, Rio de Janeiro, Brazil; 2 Laboratório de Pesquisa Clínica em Doenças Febris Agudas, Instituto Nacional de Infectologia Evandro Chagas, Fundação Oswaldo Cruz (Fiocruz), Rio de Janeiro, Brazil; 3 Laboratório de Genética Molecular de Microrganismos, Instituto Oswaldo Cruz, Fundação Oswaldo Cruz (Fiocruz), Rio de Janeiro, Brazil; 4 Centro de Pesquisa, Diagnóstico e Treinamento em Malária Reference Laboratory for Malaria in the Extra-Amazonian Region for the Brazilian Ministry of Health, SVS & Fiocruz, Rio de Janeiro, Brazil; 5 Centro Universitário Serra dos Órgãos (UNIFESO), Teresópolis, Rio de Janeiro, Brazil

**Keywords:** Malaria, *Plasmodium vivax*, Genetic diversity, SNP, DBP, Positive selection

## Abstract

**Background:**

*Plasmodium vivax* is the most widespread human malaria parasite outside Africa and is the predominant parasite in the Americas. Increasing reports of *P. vivax* disease severity, together with the emergence of drug-resistant strains, underscore the urgency of the development of vaccines against *P. vivax*. Polymorphisms on DBP-II-gene could act as an immune evasion mechanism and, consequently, limited the vaccine efficacy. This study aimed to investigate the *pvdbp*-II genetic diversity in two Brazilian regions with different epidemiological patterns: the unstable transmission area in the Atlantic Forest (AF) of Rio de Janeiro and; the fixed malaria-endemic area in Brazilian Amazon (BA).

**Methods:**

216 Brazilian *P. vivax* infected blood samples, diagnosed by microscopic examination and PCR, were investigated. The region flanking *pvdbp*-II was amplified by PCR and sequenced. Genetic polymorphisms of *pvdbp*-II were estimated based on the number of segregating sites and nucleotide and haplotype diversities; the degree of differentiation between-regions was evaluated applying Wright’s statistics. Natural selection was calculated using the rate of nonsynonymous per synonymous substitutions with the Z-test, and the evolutionary distance was estimated based on the reconstructed tree.

**Results:**

79 samples from AF and 137 from BA were successfully sequenced. The analyses showed 28 polymorphic sites distributed in 21 codons, with only 5% of the samples Salvador 1 type. The highest rates of polymorphic sites were found in B- and T cell epitopes. Unexpectedly, the nucleotide diversity in *pvdbp*-II was higher in AF (0.01) than in BA (0.008). Among the 28 SNPs detected, 18 are shared between *P. vivax* isolates from AF and BA regions, but 8 SNPs were exclusively detected in AF—I322**S**, K371**N**, E385**Q**, E385**T**, K386**T**, K411**N**, I419**L** and I419**R**—and 2 (N375**D** and I419**M**) arose exclusively in BA. These findings could suggest the potential of these geographical clusters as population-specific-signatures that may be useful to track the origin of infections. The sample size should be increased in order to confirm this possibility.

**Conclusions:**

The results highlight that the *pvdbp*-*II* polymorphisms are positively selected by host’s immune pressure. The characterization of *pvdbp*-II polymorphisms might be useful for designing effective DBP-II-based vaccines.

## Background

*Plasmodium vivax* is the most widespread human malaria parasite outside sub-Saharan Africa. Globally, 7.5 million cases in 2017 were caused by *P. vivax*, that is responsible for 37% of malaria cases in South-East Asia, 31% in the Eastern Mediterranean, and 74% in the Americas [1]. *Plasmodium vivax* causes significant morbidity, as well as social and economic burden, becoming a public health challenge in endemic countries [2]. The evidence of severe vivax malaria around the world, including Brazil [3], together with the emergence of drug-resistant strains [4], underscore the urgency to reduce the infection burden and malaria elimination [5, 6]. Therefore, the development of vaccines that protect against *P. vivax* blood stages is a priority to prevent disease and onward transmission.

The Duffy Binding Protein–DBP, a 140-kDa protein expressed in the micronemes of the *P. vivax* surface in the asexual blood-stage, interacts with the Duffy antigen/receptor for chemokines (DARC) on the host erythrocytes [7], making this molecule an attractive vaccine candidate against vivax malaria.

Currently, there are ongoing studies on *pvdbp*-II diversity. In Brazil, the unique study in this sense with Brazilian Amazon Basin samples showed codons evolving under natural selection [8]. The present study now investigates, besides Brazilian Amazon, the Rio de Janeiro Atlantic Forest region (AF) that present peculiar epidemiological characteristics [9].

## Methods

### Sample collection, diagnosis, and DNA extraction

Brazil, a country of continental proportions, has three malaria transmission profiles. The first and most important occurs inside the Brazilian Amazon Basin (BA), where more than 99% of malaria cases were recorded; the second one involves imported malaria cases, which corresponds to infections that are acquired in Brazilian endemic areas different from where the individual lives or the diagnosis has been done, or from other endemic countries. The third type of transmission represents around 0.05% of all malaria cases in Brazil and corresponds to autochthonous malaria in the Atlantic Forest (AF), located primarily along the south-eastern Atlantic Coast [9].

**Fig. 1:**
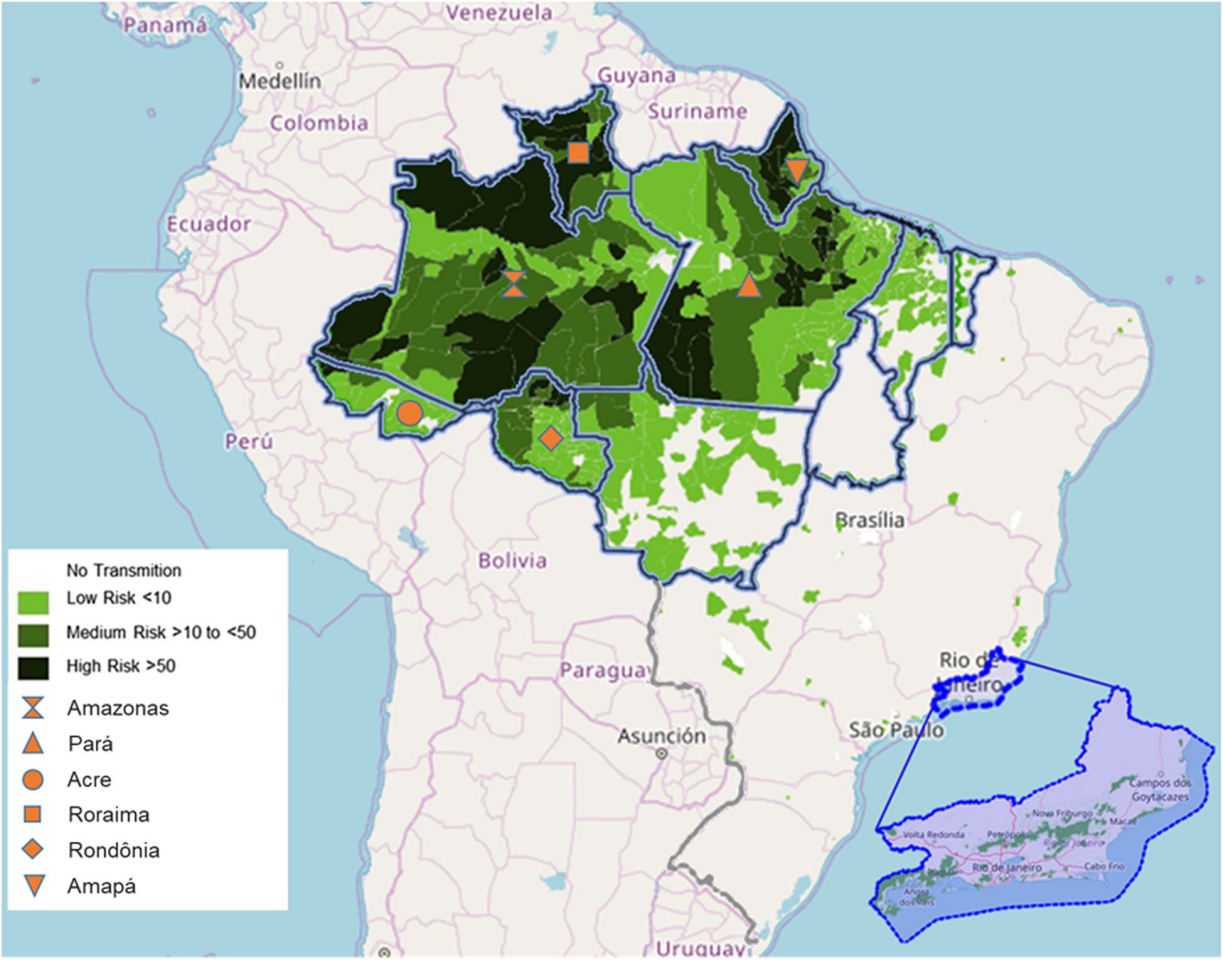
Brazilian endemic areas according to the risk (Annual Parasitic Incidence–API) (SIVEP-Malaria). Sampling regions of Rio de Janeiro Atlantic Forest and states of the Brazilian Amazon region circumscribed in blue

The polymorphisms in *pvdbp*-II were investigated in samples representing these three malaria profiles observed in Brazil. 227 *P. vivax* samples diagnosed by thin and thick blood smear and PCR were collected [13]. Two groups of patients were analysed. One group of 177 individuals (95 from BA and 82 from AF) who attended to the Reference Centre for Malaria Diagnosis CPD-Mal/Fiocruz in Rio de Janeiro (S 22° 54′ W 43° 12′) from January 2011 to March 2018; the other group of 50 individuals who sought for diagnosis at the Unit Health of Tucuruí (S 3º 46′ W 49º 40′), a municipality from Pará State, located in the Brazilian Amazon region during 2011. The samples were separated according to the state of infection (Fig. 1) and the year of blood collection (Table 1). The study was approved by the Ethics Research Committee of Instituto Oswaldo Cruz, Fiocruz, Brazil (69256317.3.0000.5248). All volunteers’ patients signed a written informed consent before collection of 4 mL of venous blood. The DNA from 1 mL blood samples was extracted using QIAamp™ DNA Blood Midi Kit (QIAGEN), according to the manufacturer’s instructions.

**Table 1:** *Plasmodium vivax* samples collected in AF and BA from 2011 to 2018

Region	State	Collection year								
		2011	2012	2013	2014	2015	2016	2017	2018	Total
BA n = 145	Acre	2	3	1	2	1	0	2	1	12
	Amapá	3	1	1	1	2	0	1	0	9
	Amazonas	2	5	6	8	4	11	10	2	48
	Para	51	1	1	1	0	0	1	0	55
	Rondônia	9	2	1	0	0	2	3	0	17
	Roraima	1	2	0	1	0	0	0	0	4
AF n = 82	Rio de Janeiro	3	2	5	0	29	14	22	7	82
Total		71	16	15	13	36	27	39	10	227

*AF* Rio de Janeiro Atlantic Forest, *BA* Brazilian Amazon

### *pvdbp*-*II* amplification

The PCR reaction to amplify a fragment of 675 bp of the DBP-II-encoding gene (amino acids from 290 to 515) was carried out with the primers 5′ ATGTATGAAGGAACTTACGAAT 3′ (forward) and 5′ ACCTGCCGTCTGAACCTTTT 3′ (reverse), as previously described [8]. The 20 μL of reaction mixture contained 2 μL (100–200 ng) of DNA, 1 μM primers (forward and reverse) and 5 × HOT FIREPol™ Blend Master Mix (Solis Biodyne), as a source of DNA polymerase, 1 mM of dNTP, 12.5 mM of MgCl_2_ and enzyme buffer (saline solution), including dyes to increase sample density during the agarose gel electrophoresis. The amplification cycles comprised: 95 ℃ for 12 min, 30 cycles of 95 ℃ for 30 s, 54 ℃ for 45 s and 72 ℃ for 2 min, followed by an extension cycle of 72 ℃ for 10 min. PCR products were purified using the kit Wizard™ SV Gel and PCR Clean-Up System (Promega), following the manufacturer’s procedure. Then, the purified forward and reverse strands were subjected to cycle sequencing with the Big Dye™ Terminator Cycle Sequencing Ready Reaction version 3.1 (Applied Biosystems) using the PCR primers at a concentration of 3.2 μM. The DNA sequencing was performed in ABI Prism DNA Analyzer™ 3730 (Applied Biosystems) with the support of Fiocruz Genomic Platform RPT01A. The sequenced reads were first analysed using NovoSNP software to investigate polymorphisms; the cutoff of electropherogram quality score was 10 to avoid losing some variations. Also, sequences were analysed in BioEdit sequence alignment editor to better-visualized SNP positions, employing ClustalW multiple sequence aligner. The Salvador 1 (Sal-1) strain was used as a reference sequence (PVX_110810, from PlasmoDB: http://www.plasmoDB.org). Sequences displaying singleton mutations and/or overlapped peaks after chromatogram inspection were re-sequenced. A single infection or the predominant variant was considered when only one nucleotide peak (allele), at any polymorphic locus of the sequence, was observed.

### Genetic diversity, natural selection, and statistical analysis

Genetic diversity of *pvdbp*-II sequences was analysed using the DnaSP 6.11 software [10] to estimate within-population diversity based on the number of segregating sites (S) and nucleotide (π) and haplotype (Hd) diversities. Wright’s fixation statistics (F) determined the degree of differentiation between-populations [11]. Evolutionary analyses were conducted with MEGA7 v7.0 software [12]. The neutrality test of evolution was calculated by Z-test using the Nei-Gojobori method [13], in which the rate of the average number of non-synonymous (dN) and synonymous (dS) SNPs define if the selective pressure is positive (dN > dS), negative (dN < dS) or neutral (dN = dS). Variance differences, in both AF and BA sequence datasets, were computed using the bootstrap method (1000 replicates). Evolutionary distances were calculated using the p-distance method [14], and the *pvdbp*-II SNP-based tree was reconstructed using the Neighbour-joining method [15] and bootstrap analysis (500 replicates) to measure accuracy. Graphs were building using GraphPad Prism software 8.1.2.

## Results

Among the 227 samples collected, 216 (95%) had the 675 bp *pvdbp*-II fragment amplified: 79 from Rio de Janeiro AF and 137 from North BA region, comprising the states of Pará (50), Amazonas (48), Acre (11), Roraima (3), Rondônia (17), and Amapá (8). The failure to satisfactory amplify 11 samples might be somehow attributed to primer limitations due to unknown polymorphisms in target sequences.

As no remarkable differences were found among *P. vivax* parasite populations from different BA states, *pvdbp*-II polymorphism will be presented, regardless of the BA state where the samples were collected. Concerning temporal differences of *pvdbp*-II polymorphism in isolates collected in the same area at different time, only in Amazonas state–São Gabriel da Cachoeira locality—several representative samples could be obtained on different occasions. In this case, temporal differences seem to occur randomly, independently of the year, and the number of haplotypes was proportional to the number of samples; the same was true in AF samples, but here the turn-over of parasite populations including identical haplotypes along the years was higher. However, as the sampling number of AF is much higher than in São Gabriel da Cachoeira, no definitive conclusions should be made (Additional file 1).

**Fig. 2:**
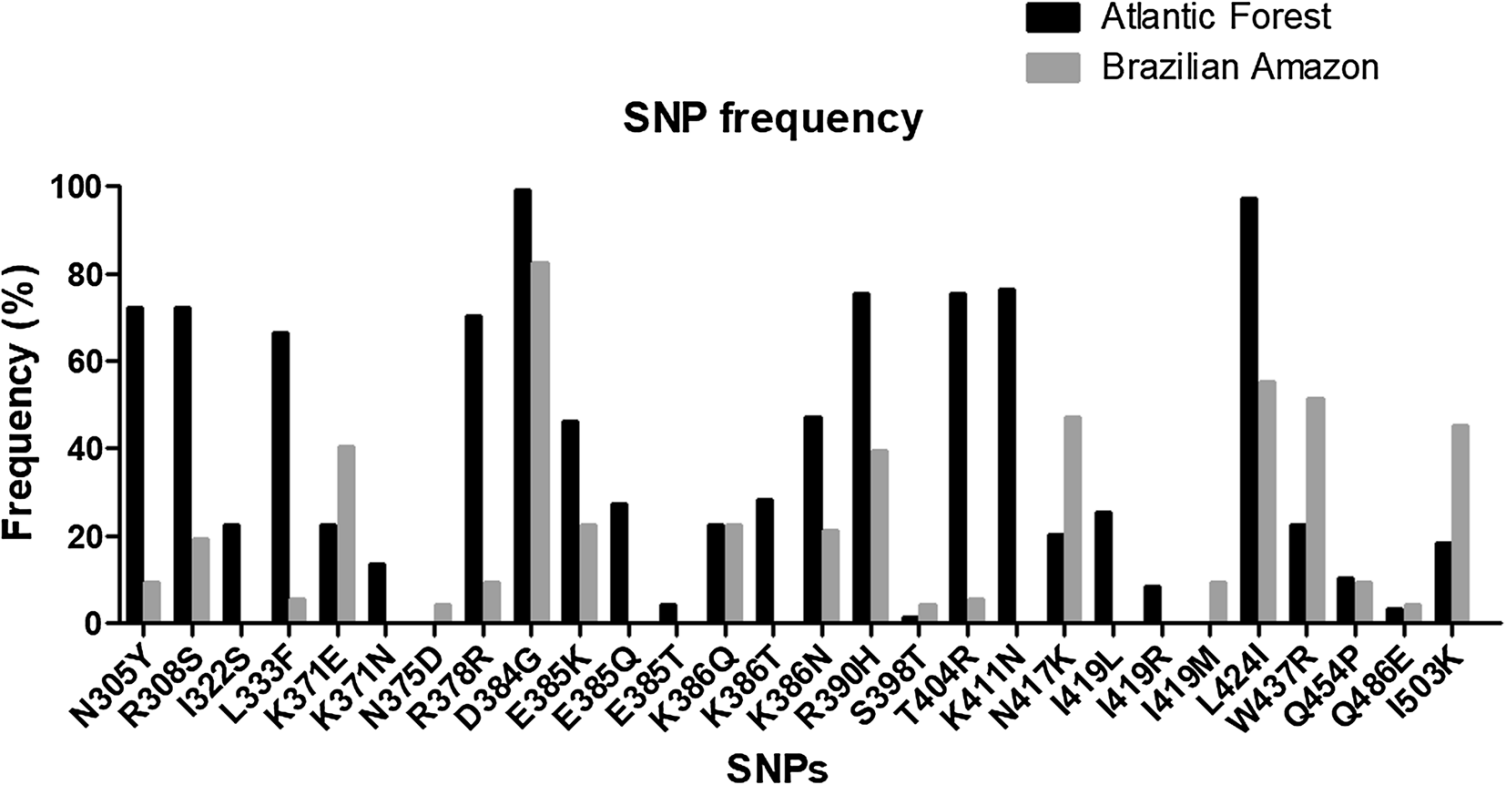
*Pvdbp*-II codons in Brazilian isolates from two Brazilian geographic regions: AF (79) and BA (137). The SNP identification code represents the amino acid in the Sal-1 reference sequence (first character), followed by the position of this residue (number), and the replacing amino acid observed at the same position (last character); Atlantic Forest from Rio de Janeiro (AF); Brazilian Amazon (BA); *N* Number, *SNPs* Single Nucleotide Polymorphisms. The graph was constructed using GraphPad Prism software 8.1.2

In all 216 Brazilian samples, the comparison of the *pvdbp*-II amplification products with the Sal-1 type strain revealed 28 polymorphic sites of which one was synonymous and 27 non-synonymous. These 28 SNPs spanned 21 codons (Table 2). Of these polymorphic codons, 19 presented only one base substitution and two codons—385 (G1153A and A1154C), and 386 (A1157C and G1158T)–presented substitutions in two bases. The 79 AF samples had 26 SNPs, of which 25 were non-synonymous. The 137 BA samples contained 20 SNPs, of which 19 are non-synonymous (Additional file 2). The AF and BA regions shared 18 SNPs, and the majority of these polymorphisms showed higher frequencies in AF (11 codons) than in BA (six codons) regions, while one codon has the same frequency in both regions (Fig. 2 and Table 2).

**Table 2:** Polymorphisms in the *pvdbp*-*II* fragment of isolates from AF and BA regions

Codons^a^	Sal-1^b^	Polymorphic sites^c^	Isolates		
			AF (79) N (%)	BA (137) N (%)	Total (216) N (%)
N305**Y**	AAT	**T**AT	57 (72)	13 (9)	70 (32)
R308**S**	AGG	AG**T**	57 (72)	26 (19)	83 (38)
I322**S**	ATT	A**G**T	17 (22)	0	0
L333**F**	CTT	**T**TT	52 (66)	7 (5)	59 (27)
K371**E**	AAA	**G**AA	17 (22)	55 (40)	72 (33)
K371**N**	AAA	AA**T**	10 (13)	0	0
N375**D**	AAT	**G**AT	0	6 (4)	0
R378**R**	CGC	CG**T**	55 (70)	12 (9)	67 (31)
D384**G**	GAT	G**G**T	78 (99)	113 (82)	191 (88)
E385**K**	GAA	**A**AA	36 (46)	30 (22)	66 (30)
E385**Q**	GAA	**C**AA	21 (27)	0	0
E385**T**	GAA	**AC**A	3 (4)	0	0
K386**Q**	AAG	**C**AG	17 (22)	30 (22)	47 (22)
K386**N**	AAG	AA**T**	37 (47)	29 (21)	66 (30)
K386**T**	AAG	A**CT**	22 (28)	0	0
R390**H**	CGT	C**A**T	59 (75)	54 (39)	113 (52)
S398**T**	TCT	**A**CT	1 (1)	6 (4)	7 (3)
T404**R**	ACA	A**G**A	59 (75)	7 (5)	66 (30)
K411**N**	AAA	AA**C**	60 (76)	0	0
N417**K**	AAT	AA**A**	16 (20)	64 (47)	80 (37)
I419**L**	ATA	**C**TA	20 (25)	0	0
I419**R**	ATA	A**G**A	6 (8)	0	0
I419**M**	ATA	AT**G**	0	13 (9)	0
L424**I**	TTA	**A**TA	77 (97)	75 (55)	152 (70)
W437**R**	TGG	**C**GG	17 (22)	70 (51)	87 (40)
Q454**P**	CAA	C**C**A	8 (10)	12 (9)	20 (9)
Q486**E**	CAA	**G**AA	2 (2)	5 (4)	7 (3)
I503**K**	ATA	A**A**A	14 (18)	62 (45)	76 (35)

^a^The first letter represents the amino acid in Sal-1 reference sequence and the last the replacing amino acid

^b^Reference Salvador 1 strain

^c^characters in bold and underlined correspond to nucleotide substitution relative to Sal-1 strain

Overall, D384**G** (191/88%), L424**I** (152/70%), and R390**H** (113/52%) SNPs were the most frequent. In AF region, D384**G** was identified in all but one sample (99%; 78), L424**I** in 97% (77), and R390**H** in 75% (59) of the samples. In BA region, D384**G** was present in 82% (113), L424**I** in 55% (75), and R390**H** in 39% (54) of the samples. SNPs I322**S** (17/22%), K371**N** (10/13%), E385**Q** (21/27%), E385**T** (3/4%), K386**T** (22/28%), K411**N** (60/76%), I419**L** (20/25%), and I419**R** (6/8%) were exclusively detected in AF while N375**D** (6/4%) and I419**M** (13/9%) were exclusively found in BA (Fig. 2).

The nucleotide diversity of *pvdbp*-II from AF (π = 0.011) was higher than those from BA (π = 0.008) (Table 3). The non-synonymous substitutions exceeded the synonymous ones (Dn > Ds) with significant expressive values in all Brazilian isolates (2.40; < 0.009), as well as when segregated AF (2.02; < 0.02) and BA (3.10; < 0.001) regions (Table 3).

**Table 3:** *Pvdbp*-*II* polymorphisms: diversity and natural selection in isolates from AF and BA regions

Diversity indexes						Neutrality test		
Region	N	S	π (SD)	Nh	Hd (SD)	dN	dS	Z-test
AF	79	26	0.011 (± 0.0007)	38	0.94 (± 0.00024)	0.0129 (± 0.003)	0.0038 (± 0.003)	2.02*
BA	137	20	0.008 (± 0.0002)	61	0.97 (± 0.00004)	0.0104 (± 0.002)	0.0012 (± 0.001)	3.10**
Total	216	28	0.012 (± 0.0002)	95	0.98 (± 0.00001)	0.0142 (± 0.003)	0.0037 (± 0.003)	2.40**

*AF* Atlantic Forest of Rio de Janeiro, *BA* Brazilian Amazon, *N* number of sequences, *S* segregating sites, *π* nucleotide diversity, *Hd* Haplotype diversity, *SD* Standard Deviation

* < 0.01; ** < 0.001

Polymorphic sites and nucleotide diversity were also investigated in eight peptides previously identified in *pvdbp*-II B- and T-cells epitopes [16, 17]. The highest number of polymorphic sites in overall Brazilian samples were found in B cell epitopes: four nsSNPs in peptides 45 (384, 385, 386, and 390) and 48 (385, 386, 390, and 398), revealing a nucleotide diversity of π = 0.051 and π = 0.047, respectively, followed by T-cell epitope in peptide 66 (π = 0.020) with one nsSNP and peptide 5 (π = 0.011) with two nsSNPs. Peptides 16 (π = 0.009), 20 (π = 0.008), 78 (π = 0.005) and 13 (π = 0.003), had only one nsSNP each one of them (Table 4). The peptides 45, 48 and 66 concentrated the higher nucleotide diversity of the *pvdbp*-II fragment.

**Table 4:** nsSNPs in T- and B-cell epitopes of *pvdbp*-II

Peptide	Residues	Epitope	N (%)	B- and T-cell epitope scores π (SD)
**5**	299-VNNTDT(N/Y)FH(R/S)DITFR-313	T/B	101 (47)	0.011 (± 0.0010)
**13**	315-LYLKRKL(I/S)YDAAVEG-329	T	17 (22)	0.003 (± 0.0006)
**16**	321-LIYDAAVEGDLL(L/F)KL-335	T/B	60 (28)	0.009 (± 0.0005)
**20**	329-GDLL(L/F)KLNNYRYNKD-343	T/B	59 (27)	0.008 (± 0.0006)
**45**	379-SIFGT(D/G)(E/K)(K/N) AQQ(R/H)RKQ-393	B	210 (97)	*0.051* (± 0.0011)
**48**	385-(E/K)(K/N)AQQ(R/H)RKQWWNE(S/T)K-399	B	207 (96)	*0.047* (± 0.0012)
**66**	421-ICK(L/I)NVAVNIEPQIY-435	T	152 (70)	*0.020* (± 0.0007)
**78**	445-YVSELPTEV(Q/P)RLKEK-459	B	20 (9)	0.005 (± 0.0009)

Previously identified epitopes in peptides **5**, **13**, **16**, **20**, **45**, **48**, **66** and **78** [16, 17]

*N* number of isolates, *π* Nucleotide diversity, the values in italic represents those with median higher than the entire *pvdbp*-II fragment (π = 0.012) and *SD* standard deviation calculated for each epitope sequence

The simultaneous presence of four SNPs in peptide 45 coding region was detected in 72% of AF isolates and only 9% of BA, whereas in peptide 48, only 1% of AF and 4% of BA showed simultaneous presence of three SNPs.

Considering that N417**K**, L424**I**, W437**R,** or I503**K** mutations could affect *pvdbp*-II antigenicity, the presence of such polymorphisms had been sought. The N417**K**—L424**I**—W437**R** combination was found in 35% (81) of the samples: 20% (16) in AF and 42% (58) in BA. Among this combination, 19% (44) of the samples bear the fourth polymorphic allele I503**K**: 9% (seven) in AF and 23% (32) in BA.

### *pvdbp*-*II* haplotypes

Among the 216 Brazilian isolates, only 11 (5%) have sequences identical to Sal-1 type (NRILKNRDEKRSTKNILWQQI): one sample from AF and ten from BA. The allele’s pattern comprised 90 haplotypes, and 59 of them (66%) were detected in one single parasite isolate each. The most polymorphic haplotypes had 15 SNPs and were only detected in AF isolates (4/6%).

**Fig. 3:**
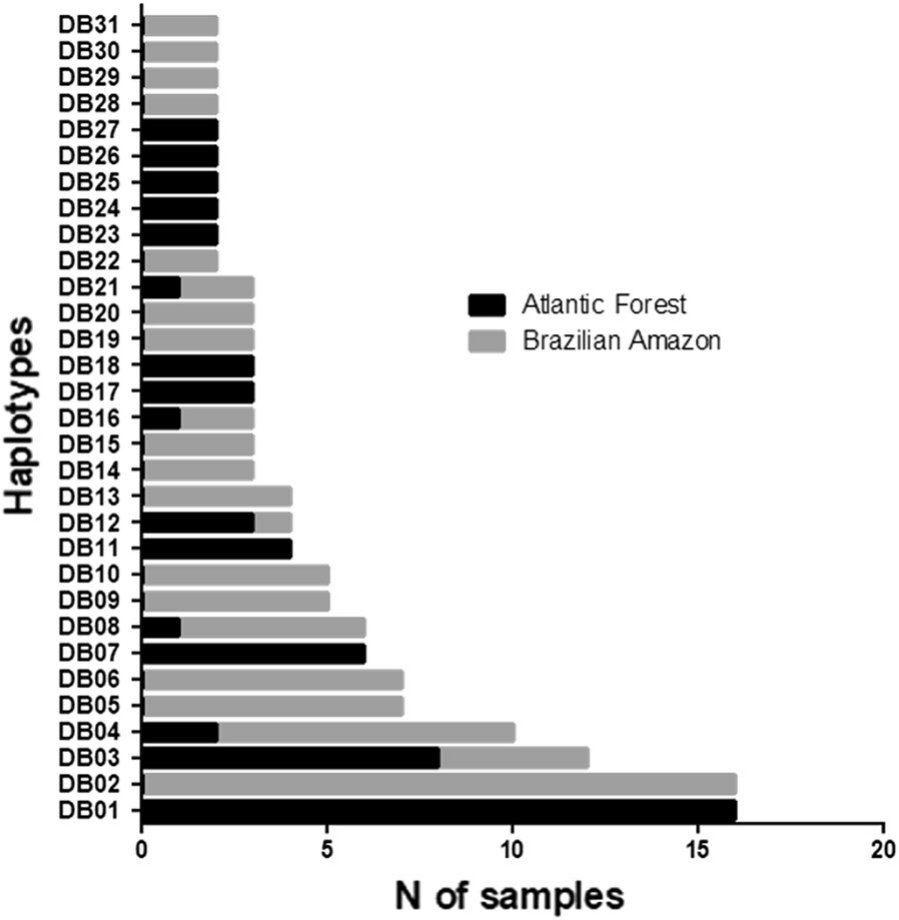
Haplotypes (DB01-DB32) presented in more than one sample from Brazilian Amazon and Atlantic Forest. *BA* Brazilian Amazon samples with 89 (65%) samples presenting 22 haplotypes (DB02, DB03, DB04, DB05, DB06, DB08, DB09, DB10, DB12, DB13, DB14, DB15, DB16, DB19, DB20, DB21, DB22, DB28, DB29, DB30, DB31 and DB32). *AF* Atlantic Forest from Rio de Janeiro isolates with 58 (73%) samples showing 16 haplotypes (DB01, DB03, DB04, DB07, DB08, DB11, DB12, DB16, DB17, DB18, DB21, DB23, DB24, DB25, DB26 and DB27). The graph was constructed using GraphPad Prism software 8.1.2

DB01 (nine SNPs) and DB02 (three SNPs) were the more frequent haplotypes: DB01 was found exclusively in AF (16/20%) and DB02 exclusively in BA (16/12%) (Fig. 3), (Additional file 3). In AF, 36 haplotypes were found, and in BA 60. Of the haplotypes found in AF samples, 30 were exclusive of this area, and of the haplotypes found in BA, 54 were exclusive of this region.

The levels of haplotype diversity (Hd) were quite similar in AF (0.94) and BA (0.97).

**Fig. 4:**
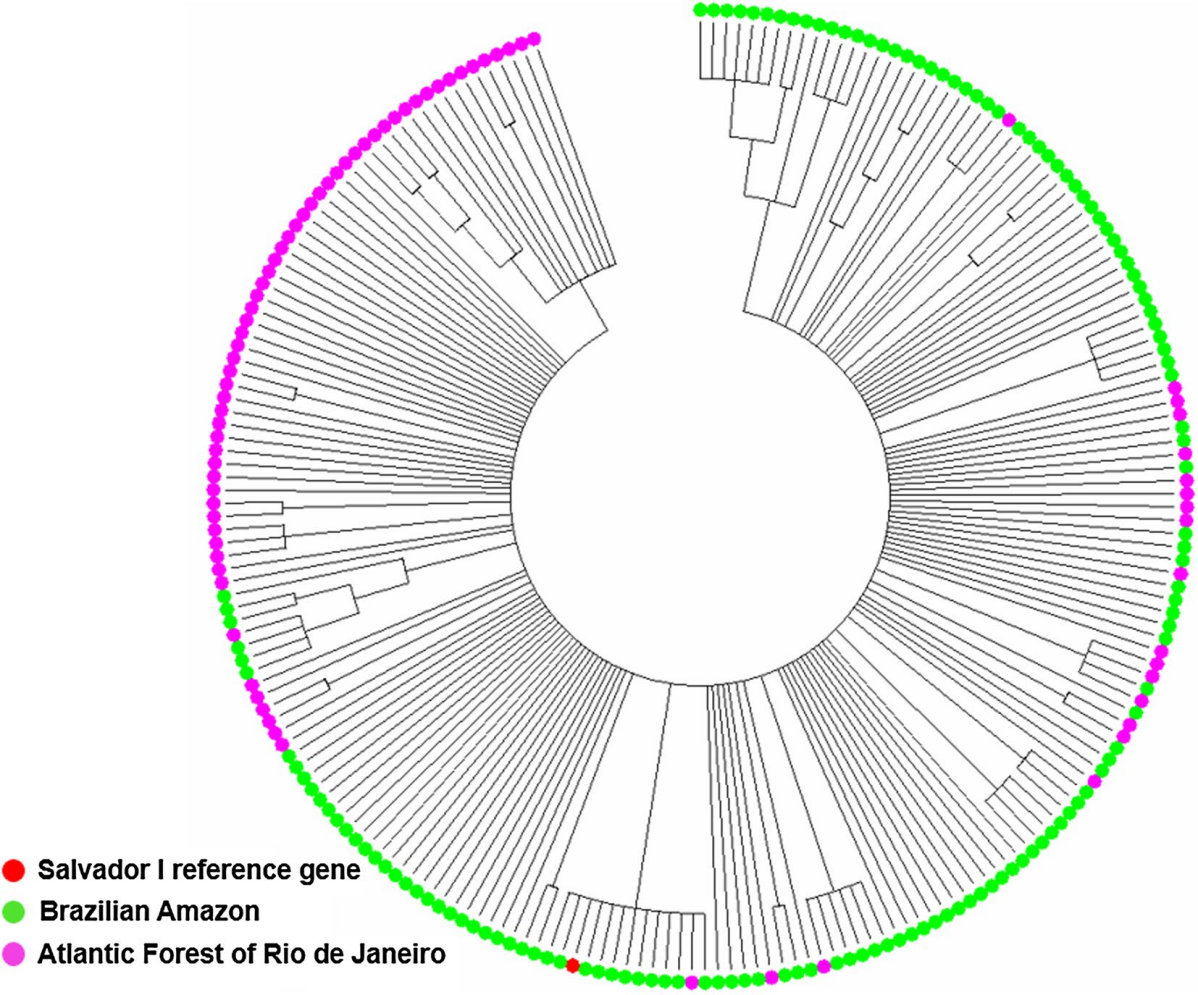
Neighbour-Joining phylogenetic tree based on *pvdbp*-II-gene SNPs of Brazilian Amazon (137) and Atlantic Forest (79) samples

The phylogenetic tree based on *pvdbp*-*II* SNPs revealed that parasites of the same geographic region shared similar profiles with few exceptions (Fig. 4). As expected, the AF samples due to its restrict geographic area were more clustering than BA. The AF samples were more distant to the Sal-1 reference strain than the BA samples. A significant degree of genetic differentiation (Fst = 0.36) was verified between AF and BA isolates.

## Discussion

This study was the first to investigate the genetic diversity of *pvdbp*-*II* outside the Amazon region, precisely in an unstable transmission area in the Atlantic Forest of Rio de Janeiro. Besides AF, *pvdbp*-II polymorphisms were also investigated in *P. vivax* isolates from the most significant Brazilian endemic region to expand the knowledge on parasite population diversity in Brazil and compare the data with those from other endemic countries. In this way, only 5% of field samples presented the *pvdbp*-II sequence utterly identical to the *pvdbp*-*II* of Salvador 1 type strain. The polymorphism degree comprised 28 polymorphic sites in 21 codons, with only one sSNP. The eighteen nsSNPs found in *P. vivax* isolates here studied, including those with higher frequencies—D384**G** and L424**I** were previously reported in *P. vivax* isolates from Africa, Oceania, Asia, and other South America countries [18–21]. The frequency of these SNPs was similar to this study, as in the case of Thailand [18], Myanmar [19], Sudan [21], and Papua New Guinea [17], or smaller as in the case of Sri-Lanka [22]. These data reinforce the idea that *pvdbp*-II diversity seems to occur independently of the malaria endemicity levels [23, 24].

Further, eight SNPs were exclusively detected in AF, and, interestingly, two of them (K371**N** and E385**Q**/**K**) had been previously found in AF areas of Santa Catarina state [25]. Likewise, two previously described SNPs (N375**D** and I419**M**) in the Brazilian Amazon region [26] and Asia [18, 19], arose exclusively in BA. These findings could suggest the potential of AF geographical clusters as population-specific-signatures that may be useful to track the origin of infections. However, more studies are required to confirm that these SNPs are confined to the determined geographic area.

Overall, the SNP-based phylogenetic tree of the *pvdbp*-*II* revealed two genetic subdivisions, one for AF and another for BA, showing that same geographic region isolates share similar evolutionary histories. BA samples are spread out in several clades, while AF samples show a small number of clades, possibly due to the tremendous difference in territorial size between these two geographic regions. The genetic subdivision of *P. vivax* Brazilian populations was also supported by a high Fst value [17], reflecting the limited gene flow between parasites of AF and BA. The high frequency of exclusive nsSNP K411**N** present in 76% of the isolates of AF could demonstrate the fixation of this SNP, probably, by positive natural selection on *pvdbp*-*II* mediated by host pressures. Inclusive, the hypothesis of positive selection could be sustained by the increased number of non-synonymous substitutions (Dn > Ds) assessed by the Z-test.

It is well known that nsSNPs could change B and/or T cell epitopes, therefore affecting the host immune response. In this study, the nucleotide diversity was higher in B and/or T cell epitopes than in the whole 675 bp amplified fragmented, highlighting a positive selection mediated by host immune system, that was supported by significant positive values in neutrality test. Except for I322**S** nsSNPs, exclusively detected in AF isolates, all other epitope polymorphisms were already reported in Brazil [8], Papua New Guinea [17], Thailand [20], Colombia and South Korea [17], demonstrating the global distribution of these epitope mutated alleles.

BA is a Brazilian region where malaria transmission effectively occurs, and much more meiotic recombination generating polymorphism is expected. Nevertheless, the SNP frequency in AF was higher than in BA, suggesting that besides recombination, the host immune response is an essential natural selection factor promoting fixation of mutated *pvdbp*-II parasite populations.

## Conclusion

The results highlight that the *pvdbp*-*II* polymorphisms are positively selected by host’s immune response pressure. The characterization of *pvdbp*-*II* polymorphisms might be useful for designing effective DBP-II-based vaccines.

## Supplementary information

**Additional file 1.***Pvdbp*-II-gene haplotypes in São Gabriel da Cachoeira (BA) and in Rio de Janeiro (AF).

**Additional file 2.** Multiple sequence alignment of *pvdbp-II* from AF and BA regions. Multiple alignment of 675 bp *pvdbp*-II fragment of field isolates compared to Salvador 1 reference sequence (PVX_110810).

**Additional file 3.***Pvdbp-II* haplotypes. Identification of 90 haplotypes and their respective nucleotide sequence compared to Salvador 1 reference sequence (PVX_110810).

## Data Availability

All data generated or analysed during this study are included in this article (and its Additional files). Sequence data that support the findings of this study have been deposited in GenBank with the accession codes MN223747 to MN223974.

## Abbreviations

WHO: World Health Organization
*Pvdbp*-II: *Plasmodium vivax* duffy binding protein-region II
DARC: Duffy antigen/receptor for chemokines
AF: Atlantic Forest
BA: Brazilian Amazon
π: Nucleotide diversity
S: Polymorphic sites
Hd: Haplotype diversity
Fst: Wright’s fixation statistics
SNP: Single nucleotide polymorphism
sSNP: Synonymous single nucleotide polymorphism
nsSNP: Non-synonymous single nucleotide polymorphism
