# Supplementary material for: Extensive genetic diversity of Plasmodium vivax dbp-II in Rio de Janeiro Atlantic Forest and Brazilian Amazon Basin: evidence of positive selection
Source: Malar J. 2020 Feb 19;19:81. doi: 10.1186/s12936-020-03159-y (PMC7031913; doi:10.1186/s12936-020-03159-y)
Supplement: Supplementary file 1 — Additional file 1.Pvdbp-II-gene haplotypes in São Gabriel da Cachoeira (BA) and in Rio de Janeiro (AF). [file 12936_2020_3159_MOESM1_ESM.pdf]

PvDBP-II-gene haplotypes in Sao Gabriel da Cachoeira (BA) and in Rio de Janeiro (AF).

**Amazonas**		**2011**	**2012**	**2013**	**2014**	**2015**	**2016**	**2017**	**2018**	**Total**
São Gabriel da Cachoeira	n of samples	0	0	0	5	1	4	4	1	15
	n of haplotypes	0	0	0	5	1	2	4	1	14
	haplotype types	0	0	0	DB03,DB20, DB40, B41, DB58	DB29	**DB06** (3 samples) DB08 (1 sample)	DB08, DB20, DB21, B72	DB09	0
	Sal 1 Type	0	0	0	0	0	0	0	0	0
**AF**		**2011**	**2012**	**2013**	**2014**	**2015**	**2016**	**2017**	**2018**	**Total**
	n of samples	3	3	5	0	28	14	19	7	79
	n of haplotypes	3	2	5	0	14	10	12	5	51
	haplotype types	DB12, DB55	DB12, DB23	DB01, DB11, DB18, DB23, DB53	0	DB01, DB03, DB07, DB11, DB17, DB24, **DB27**, DB34, DB43, DB44, DB45, DB50, DB60, DB86	DB01, DB03, DB07, DB17, DB24, **DB25**, DB38, DB46, DB52, DB59	DB01, DB03, DB07, DB08, DB21, **DB26**, DB33, DB35, DB36, DB39, DB47, DB61	DB01, DB04, DB16, DB18, DB80	
	Sal 1 Type	1	0	0	0	0	0	0	0	1

Haplotype in black bold appear in more than one sample at the same year
