# Supplementary material for: Extensive genetic diversity of Plasmodium vivax dbp-II in Rio de Janeiro Atlantic Forest and Brazilian Amazon Basin: evidence of positive selection
Source: Malar J. 2020 Feb 19;19:81. doi: 10.1186/s12936-020-03159-y (PMC7031913; doi:10.1186/s12936-020-03159-y)
Supplement: Supplementary file 2 — Additional file 2. Multiple sequence alignment of pvdbp-II from AF and BA regions. Multiple alignment of 675 bp pvdbp-II fragment of field isolates compared to Salvador 1 reference sequence (PVX_110810). [file 12936_2020_3159_MOESM2_ESM.pdf]

```
                            10         20         30         40         50         60         70         80         90        100
                    ....|....| ....|....| ....|....| ....|....| ....|....| ....|....| ....|....| ....|....| ....|....| ....|....|
PVX_110810_|_Salvador 1  ATGTATGAAG GAACTTACGA ATTTGGTAAA TAATACAGAC ACAAATTTTC ATAGGGATAT AACATTTCGA AAATTATATT TGAAAAGGAA ACTTATTTAT
DBP_07_Brazil-BA    .......... .......... .......... .......... .......... ....T..... .......... .......... .......... ..........
DBP_09_Brazil-BA    .......... .......... .......... .......... .......... .......... .......... .......... .......... ..........
DBP_10_Brazil-BA    .......... .......... .......... .......... .......... .......... .......... .......... .......... ..........
DBP_11_Brazil-BA    .......... .......... .......... .......... .......... .......... .......... .......... .......... ..........
DBP_12_Brazil-BA    .......... .......... .......... .......... .......... ....T..... .......... .......... .......... ..........
DBP_13_Brazil-BA    .......... .......... .......... .......... .......... .......... .......... .......... .......... ..........
DBP_14_Brazil-BA    .......... .......... .......... .......... .......... .......... .......... .......... .......... ..........
DBP_15_Brazil-BA    .......... .......... .......... .......... .......... .......... .......... .......... .......... ..........
DBP_16_Brazil-BA    .......... .......... .......... .......... .......... ....T..... .......... .......... .......... ..........
DBP_17_Brazil-BA    .......... .......... .......... .......... .......... ....T..... .......... .......... .......... ..........
DBP_18_Brazil-BA    .......... .......... .......... .......... .......... .......... .......... .......... .......... ..........
DBP_20_Brazil-BA    .......... .......... .......... .......... .......... .......... .......... .......... .......... ..........
DBP_21_Brazil-BA    .......... .......... .......... .......... .......... .......... .......... .......... .......... ..........
DBP_22_Brazil-BA    .......... .......... .......... .......... .......... .......... .......... .......... .......... ..........
DBP_24_Brazil-BA    .......... .......... .......... .......... .......... .......... .......... .......... .......... ..........
DBP_26_Brazil-BA    .......... .......... .......... .......... .......... .......... .......... .......... .......... ..........
DBP_27_Brazil-BA    .......... .......... .......... .......... .......... .......... .......... .......... .......... ..........
DBP_28_Brazil-BA    .......... .......... .......... .......... .......... .......... .......... .......... .......... ..........
DBP_29_Brazil-BA    .......... .......... .......... .......... .......... .......... .......... .......... .......... ..........
DBP_31_Brazil-BA    .......... .......... .......... .......... .......... .......... .......... .......... .......... ..........
DBP_32_Brazil-BA    .......... .......... .......... .......... .......... .......... .......... .......... .......... ..........
DBP_33_Brazil-BA    .......... .......... .......... .......... .......... .......... .......... .......... .......... ..........
DBP_35_Brazil-BA    .......... .......... .......... .......... .......... .......... .......... .......... .......... ..........
DBP_36_Brazil-BA    .......... .......... .......... .......... .......... .......... .......... .......... .......... ..........
DBP_37_Brazil-BA    .......... .......... .......... .......... .......... .......... .......... .......... .......... ..........
DBP_38_Brazil-BA    .......... .......... .......... .......... .......... .......... .......... .......... .......... ..........
DBP_39_Brazil-BA    .......... .......... .......... .......... .......... .......... .......... .......... .......... ..........
DBP_40_Brazil-BA    .......... .......... .......... .......... .......... .......... .......... .......... .......... ..........
DBP_41_Brazil-BA    .......... .......... .......... .......... .......... .......... .......... .......... .......... ..........
DBP_42_Brazil-BA    .......... .......... .......... .......... .......... .......... .......... .......... .......... ..........
DBP_43_Brazil-BA    .......... .......... .......... .......... .......... .......... .......... .......... .......... ..........
DBP_44_Brazil-BA    .......... .......... .......... .......... .......... .......... .......... .......... .......... ..........
DBP_45_Brazil-BA    .......... .......... .......... .......... .......... .......... .......... .......... .......... ..........
DBP_46_Brazil-BA    .......... .......... .......... .......... .......... .......... .......... .......... .......... ..........
DBP_47_Brazil-BA    .......... .......... .......... .......... .......... .......... .......... .......... .......... ..........
DBP_48_Brazil-BA    .......... .......... .......... .......... .......... .......... .......... .......... .......... ..........
DBP_49_Brazil-BA    .......... .......... .......... .......... .......... .......... .......... .......... .......... ..........
DBP_51_Brazil-BA    .......... .......... .......... .......... .......... .......... .......... .......... .......... ..........
DBP_53_Brazil-BA    .......... .......... .......... .......... .......... .......... .......... .......... .......... ..........
DBP_54_Brazil-BA    .......... .......... .......... .......... .......... .......... .......... .......... .......... ..........
DBP_55_Brazil-BA    .......... .......... .......... .......... .......... .......... .......... .......... .......... ..........
DBP_56_Brazil-BA    .......... .......... .......... .......... .......... .......... .......... .......... .......... ..........
DBP_57_Brazil-BA    .......... .......... .......... .......... .......... .......... .......... .......... .......... ..........
DBP_58_Brazil-BA    .......... .......... .......... .......... .......... .......... .......... .......... .......... ..........
DBP_59_Brazil-BA    .......... .......... .......... .......... .......... .......... .......... .......... .......... ..........
DBP_1161_Brazil-BA  .......... .......... .......... .......... ...T...... ....T..... .......... .......... .......... ..........
DBP_1182_Brazil-BA  .......... .......... .......... .......... .......... .......... .......... .......... .......... ..........
DBP_1190_Brazil-BA  .......... .......... .......... .......... .......... .......... .......... .......... .......... ..........
```

```
DBP_1200_Brazil-BA    .......... .......... .......... .......... .......... .......... .......... .......... .......... ..........
DBP_1209_Brazil-BA    .......... .......... .......... .......... .......... .......... .......... .......... .......... ..........
DBP_1272_Brazil-BA    .......... .......... .......... .......... .......... .......... .......... .......... .......... ..........
DBP_1295_Brazil-BA    .......... .......... .......... .......... .......... .......... .......... .......... .......... ..........
DBP_1320_Brazil-BA    .......... .......... .......... .......... .......... .......... .......... .......... .......... ..........
DBP_1327_Brazil-BA    .......... .......... .......... .......... .......... .......... .......... .......... .......... ..........
DBP_1335_Brazil-BA    .......... .......... .......... .......... ...T...... .......... .......... .......... .......... ..........
DBP_1340_Brazil-BA    .......... .......... .......... .......... .......... ....T..... .......... .......... .......... ..........
DBP_1346_Brazil-BA    .......... .......... .......... .......... .......... .......... .......... .......... .......... ..........
DBP_1382_Brazil-BA    .......... .......... .......... .......... .......... .......... .......... .......... .......... ..........
DBP_1428_Brazil-BA    .......... .......... .......... .......... .......... .......... .......... .......... .......... ..........
DBP_1447_Brazil-BA    .......... .......... .......... .......... ...T...... .......... .......... .......... .......... ..........
DBP_1469_Brazil-BA    .......... .......... .......... .......... ...T...... ....T..... .......... .......... .......... ..........
DBP_1473_Brazil-BA    .......... .......... .......... .......... .......... ....T..... .......... .......... .......... ..........
DBP_1475_Brazil-BA    .......... .......... .......... .......... ...T...... .......... .......... .......... .......... ..........
DBP_1493_Brazil-BA    .......... .......... .......... .......... .......... ....T..... .......... .......... .......... ..........
DBP_1532_Brazil-BA    .......... .......... .......... .......... .......... .......... .......... .......... .......... ..........
DBP_1540_Brazil-BA    .......... .......... .......... .......... .......... ....T..... .......... .......... .......... ..........
DBP_1621_Brazil-BA    .......... .......... .......... .......... ...T...... .......... .......... .......... .......... ..........
DBP_1627_Brazil-BA    .......... .......... .......... .......... ...T...... .......... .......... .......... .......... ..........
DBP_1641_Brazil-BA    .......... .......... .......... .......... .......... .......... .......... .......... .......... ..........
DBP_1664_Brazil-BA    .......... .......... .......... .......... .......... .......... .......... .......... .......... ..........
DBP_1741_Brazil-BA    .......... .......... .......... .......... ...T...... .......... .......... .......... .......... ..........
DBP_1747_Brazil-BA    .......... .......... .......... .......... .......... .......... .......... .......... .......... ..........
DBP_1748_Brazil-BA    .......... .......... .......... .......... .......... .......... .......... .......... .......... ..........
DBP_1777_Brazil-BA    .......... .......... .......... .......... .......... .......... .......... .......... .......... ..........
DBP_1789_Brazil-BA    .......... .......... .......... .......... .......... .......... .......... .......... .......... ..........
DBP_1812_Brazil-BA    .......... .......... .......... .......... ...T...... .......... .......... .......... .......... ..........
DBP_1824_Brazil-BA    .......... .......... .......... .......... .......... .......... .......... .......... .......... ..........
DBP_1830_Brazil-BA    .......... .......... .......... .......... .......... ....T..... .......... .......... .......... ..........
DBP_1837_Brazil-BA    .......... .......... .......... .......... .......... .......... .......... .......... .......... ..........
DBP_1853_Brazil-BA    .......... .......... .......... .......... .......... .......... .......... .......... .......... ..........
DBP_1867_Brazil-BA    .......... .......... .......... .......... .......... .......... .......... .......... .......... ..........
DBP_1914_Brazil-BA    .......... .......... .......... .......... .......... ....T..... .......... .......... .......... ..........
DBP_1874_Brazil-BA    .......... .......... .......... .......... .......... .......... .......... .......... .......... ..........
DBP_2036_Brazil-BA    .......... .......... .......... .......... .......... .......... .......... .......... .......... ..........
DBP_2159_Brazil-BA    .......... .......... .......... .......... .......... .......... .......... .......... .......... ..........
DBP_2212_Brazil-BA    .......... .......... .......... .......... .......... .......... .......... .......... .......... ..........
DBP_2272_Brazil-BA    .......... .......... .......... .......... .......... .......... .......... .......... .......... ..........
DBP_2327_Brazil-BA    .......... .......... .......... .......... ...T...... .......... .......... .......... .......... ..........
DBP_2339_Brazil-BA    .......... .......... .......... .......... .......... .......... .......... .......... .......... ..........
DBP_2452_Brazil-BA    .......... .......... .......... .......... .......... .......... .......... .......... .......... ..........
DBP_2462_Brazil-BA    .......... .......... .......... .......... .......... .......... .......... .......... .......... ..........
DBP_2517_Brazil-BA    .......... .......... .......... .......... .......... .......... .......... .......... .......... ..........
DBP_2592_Brazil-BA    .......... .......... .......... .......... .......... .......... .......... .......... .......... ..........
DBP_2600_Brazil-BA    .......... .......... .......... .......... .......... .......... .......... .......... .......... ..........
DBP_2650_Brazil-BA    .......... .......... .......... .......... .......... ....T..... .......... .......... .......... ..........
DBP_2688_Brazil-BA    .......... .......... .......... .......... .......... .......... .......... .......... .......... ..........
DBP_2695_Brazil-BA    .......... .......... .......... .......... .......... .......... .......... .......... .......... ..........
DBP_2692_Brazil-BA    .......... .......... .......... .......... ...T...... ....T..... .......... .......... .......... ..........
DBP_2693_Brazil-BA    .......... .......... .......... .......... ...T...... ....T..... .......... .......... .......... ..........
```

```
DBP_2705_Brazil-BA    .......... .......... .......... .......... .......... .......... .......... .......... .......... ..........
DBP_2737_Brazil-BA    .......... .......... .......... .......... .......... .......... .......... .......... .......... ..........
DBP_2751_Brazil-BA    .......... .......... .......... .......... .......... .......... .......... .......... .......... ..........
DBP_2773_Brazil-BA    .......... .......... .......... .......... .......... .......... .......... .......... .......... ..........
DBP_3124_Brazil-BA    .......... .......... .......... .......... .......... .......... .......... .......... .......... ..........
DBP_3164_Brazil-BA    .......... .......... .......... .......... .......... ....T..... .......... .......... .......... ..........
DBP_3260_Brazil-BA    .......... .......... .......... .......... .......... ....T..... .......... .......... .......... ..........
DBP_3280_Brazil-BA    .......... .......... .......... .......... .......... ....T..... .......... .......... .......... ..........
DBP_3307_Brazil-BA    .......... .......... .......... .......... .......... .......... .......... .......... .......... ..........
DBP_3311_Brazil-BA    .......... .......... .......... .......... .......... ....T..... .......... .......... .......... ..........
DBP_3314_Brazil-BA    .......... .......... .......... .......... .......... .......... .......... .......... .......... ..........
DBP_3340_Brazil-BA    .......... .......... .......... .......... .......... ....T..... .......... .......... .......... ..........
DBP_3404_Brazil-BA    .......... .......... .......... .......... .......... ....T..... .......... .......... .......... ..........
DBP_3507_Brazil-BA    .......... .......... .......... .......... .......... .......... .......... .......... .......... ..........
DBP_3516_Brazil-BA    .......... .......... .......... .......... .......... .......... .......... .......... .......... ..........
DBP_3530_Brazil-BA    .......... .......... .......... .......... .......... .......... .......... .......... .......... ..........
DBP_3556_Brazil-BA    .......... .......... .......... .......... .......... .......... .......... .......... .......... ..........
DBP_3560_Brazil-BA    .......... .......... .......... .......... .......... .......... .......... .......... .......... ..........
DBP_3569_Brazil-BA    .......... .......... .......... .......... .......... .......... .......... .......... .......... ..........
DBP_3601_Brazil-BA    .......... .......... .......... .......... .......... .......... .......... .......... .......... ..........
DBP_3617_Brazil-BA    .......... .......... .......... .......... ...T...... .......... .......... .......... .......... ..........
DBP_3661_Brazil-BA    .......... .......... .......... .......... .......... .......... .......... .......... .......... ..........
DBP_3707_Brazil-BA    .......... .......... .......... .......... .......... .......... .......... .......... .......... ..........
DBP_3814_Brazil-BA    .......... .......... .......... .......... .......... .......... .......... .......... .......... ..........
DBP_3842_Brazil-BA    .......... .......... .......... .......... .......... .......... .......... .......... .......... ..........
DBP_3876_Brazil-BA    .......... .......... .......... .......... .......... .......... .......... .......... .......... ..........
DBP_3880_Brazil-BA    .......... .......... .......... .......... .......... .......... .......... .......... .......... ..........
DBP_3908_Brazil-BA    .......... .......... .......... .......... .......... .......... .......... .......... .......... ..........
DBP_3913_Brazil-BA    .......... .......... .......... .......... .......... ....T..... .......... .......... .......... ..........
DBP_3914_Brazil-BA    .......... .......... .......... .......... .......... .......... .......... .......... .......... ..........
DBP_3937_Brazil-BA    .......... .......... .......... .......... .......... ....T..... .......... .......... .......... ..........
DBP_3974_Brazil-BA    .......... .......... .......... .......... .......... ....T..... .......... .......... .......... ..........
DBP_3976_Brazil-BA    .......... .......... .......... .......... .......... ....T..... .......... .......... .......... ..........
DBP_3998_Brazil-BA    .......... .......... .......... .......... .......... .......... .......... .......... .......... ..........
DBP_4030_Brazil-BA    .......... .......... .......... .......... .......... .......... .......... .......... .......... ..........
DBP_4071_Brazil-BA    .......... .......... .......... .......... .......... .......... .......... .......... .......... ..........
DBP_4109_Brazil-BA    .......... .......... .......... .......... .......... .......... .......... .......... .......... ..........
DBP_4135_Brazil-BA    .......... .......... .......... .......... .......... ....T..... .......... .......... .......... ..........
DBP_1274_Brazil-AF    .......... .......... .......... .......... ...T...... .......... .......... .......... .......... ..........
DBP_1347_Brazil-AF    .......... .......... .......... .......... .......... .......... .......... .......... .......... ..........
DBP_1436_Brazil-AF    .......... .......... .......... .......... .......... ....T..... .......... .......... .......... ..........
DBP_1737_Brazil-AF    .......... .......... .......... .......... ...T...... .......... .......... .......... .......... ..........
DBP_1652_Brazil-AF    .......... .......... .......... .......... ...T...... ....T..... .......... .......... .......... ......G...
DBP_1745_Brazil-AF    .......... .......... .......... .......... ...T...... .......... .......... .......... .......... ..........
DBP_1856_Brazil-AF    .......... .......... .......... .......... ...T...... ....T..... .......... .......... .......... ..........
DBP_1906_Brazil-AF    .......... .......... .......... .......... .......... ....T..... .......... .......... .......... ..........
DBP_1948_Brazil-AF    .......... .......... .......... .......... ...T...... ....T..... .......... .......... .......... ......G...
DBP_1960_Brazil-AF    .......... .......... .......... .......... ...T...... ....T..... .......... .......... .......... ..........
DBP_1961_Brazil-AF    .......... .......... .......... .......... ...T...... ....T..... .......... .......... .......... ......G...
DBP_2728_Brazil-AF    .......... .......... .......... .......... ...T...... ....T..... .......... .......... .......... ..........
DBP_2734_Brazil-AF    .......... .......... .......... .......... .......... .......... .......... .......... .......... ..........
```

```
DBP_2736_Brazil-AF    .......... .......... .......... .......... .......... .......... .......... .......... .......... ..........
DBP_2747_Brazil-AF    .......... .......... .......... .......... ...T...... .......... .......... .......... .......... ..........
DBP_2752_Brazil-AF    .......... .......... .......... .......... ...T...... ....T..... .......... .......... .......... ..........
DBP_2762_Brazil-AF    .......... .......... .......... .......... ...T...... ....T..... .......... .......... .......... ..........
DBP_2765_Brazil-AF    .......... .......... .......... .......... ...T...... ....T..... .......... .......... .......... ..........
DBP_2769_Brazil-AF    .......... .......... .......... .......... ...T...... ....T..... .......... .......... .......... ..........
DBP_2770_Brazil-AF    .......... .......... .......... .......... ...T...... ....T..... .......... .......... .......... ..........
DBP_2775_Brazil-AF    .......... .......... .......... .......... ...T...... ....T..... .......... .......... .......... ......G...
DBP_2779_Brazil-AF    .......... .......... .......... .......... ...T...... ....T..... .......... .......... .......... ..........
DBP_2782_Brazil-AF    .......... .......... .......... .......... ...T...... ....T..... .......... .......... .......... ..........
DBP_2792_Brazil-AF    .......... .......... .......... .......... ...T...... ....T..... .......... .......... .......... ......G...
DBP_2793_Brazil-AF    .......... .......... .......... .......... ...T...... ....T..... .......... .......... .......... ......G...
DBP_2816_Brazil-AF    .......... .......... .......... .......... ...T...... ....T..... .......... .......... .......... ......G...
DBP_2840_Brazil-AF    .......... .......... .......... .......... ...T...... ....T..... .......... .......... .......... ..........
DBP_2859_Brazil-AF    .......... .......... .......... .......... ...T...... ....T..... .......... .......... .......... ..........
DBP_2900_Brazil-AF    .......... .......... .......... .......... ...T...... ....T..... .......... .......... .......... ..........
DBP_2916_Brazil-AF    .......... .......... .......... .......... ...T...... ....T..... .......... .......... .......... ......G...
DBP_2923_Brazil-AF    .......... .......... .......... .......... ...T...... ....T..... .......... .......... .......... ..........
DBP_2980_Brazil-AF    .......... .......... .......... .......... .......... .......... .......... .......... .......... ..........
DBP_2982_Brazil-AF    .......... .......... .......... .......... ...T...... ....T..... .......... .......... .......... ......G...
DBP_2983_Brazil-AF    .......... .......... .......... .......... .......... .......... .......... .......... .......... ......G...
DBP_3071_Brazil-AF    .......... .......... .......... .......... ...T...... ....T..... .......... .......... .......... ..........
DBP_3079_Brazil-AF    .......... .......... .......... .......... ...T...... ....T..... .......... .......... .......... ......G...
DBP_3084_Brazil-AF    .......... .......... .......... .......... ...T...... ....T..... .......... .......... .......... ..........
DBP_3089_Brazil-AF    .......... .......... .......... .......... ...T...... ....T..... .......... .......... .......... ..........
DBP_3157_Brazil-AF    .......... .......... .......... .......... .......... .......... .......... .......... .......... ..........
DBP_3276_Brazil-AF    .......... .......... .......... .......... .......... .......... .......... .......... .......... ..........
DBP_3277_Brazil-AF    .......... .......... .......... .......... ...T...... ....T..... .......... .......... .......... ..........
DBP_3278_Brazil-AF    .......... .......... .......... .......... .......... ....T..... .......... .......... .......... ......G...
DBP_3284_Brazil-AF    .......... .......... .......... .......... ...T...... ....T..... .......... .......... .......... ..........
DBP_3296_Brazil-AF    .......... .......... .......... .......... ...T...... ....T..... .......... .......... .......... ..........
DBP_3300_Brazil-AF    .......... .......... .......... .......... ...T...... ....T..... .......... .......... .......... ..........
DBP_3310_Brazil-AF    .......... .......... .......... .......... ...T...... ....T..... .......... .......... .......... ..........
DBP_3315_Brazil-AF    .......... .......... .......... .......... ...T...... ....T..... .......... .......... .......... ..........
DBP_3325_Brazil-AF    .......... .......... .......... .......... .......... .......... .......... .......... .......... ..........
DBP_3334_Brazil-AF    .......... .......... .......... .......... ...T...... ....T..... .......... .......... .......... ......G...
DBP_3371_Brazil-AF    .......... .......... .......... .......... ...T...... ....T..... .......... .......... .......... ......G...
DBP_3376_Brazil-AF    .......... .......... .......... .......... ...T...... ....T..... .......... .......... .......... ..........
DBP_3379_Brazil-AF    .......... .......... .......... .......... ...T...... ....T..... .......... .......... .......... ..........
DBP_3406_Brazil-AF    .......... .......... .......... .......... .......... ....T..... .......... .......... .......... ..........
DBP_3612_Brazil-AF    .......... .......... .......... .......... ...T...... ....T..... .......... .......... .......... ..........
DBP_3613_Brazil-AF    .......... .......... .......... .......... ...T...... ....T..... .......... .......... .......... ..........
DBP_3614_Brazil-AF    .......... .......... .......... .......... ...T...... ....T..... .......... .......... .......... ......G...
DBP_3616_Brazil-AF    .......... .......... .......... .......... ...T...... ....T..... .......... .......... .......... ......G...
DBP_3630_Brazil-AF    .......... .......... .......... .......... ...T...... ....T..... .......... .......... .......... ..........
DBP_3633_Brazil-AF    .......... .......... .......... .......... ...T...... ....T..... .......... .......... .......... ......G...
DBP_3643_Brazil-AF    .......... .......... .......... .......... ...T...... ....T..... .......... .......... .......... ..........
DBP_3659_Brazil-AF    .......... .......... .......... .......... ...T...... ....T..... .......... .......... .......... ..........
DBP_3698_Brazil-AF    .......... .......... .......... .......... ...T...... ....T..... .......... .......... .......... ..........
DBP_3705_Brazil-AF    .......... .......... .......... .......... .......... .......... .......... .......... .......... ..........
DBP_3722_Brazil-AF    .......... .......... .......... .......... ...T...... ....T..... .......... .......... .......... ..........
```

```
DBP_3735_Brazil-AF        .......... .......... .......... .......... ...T...... ....T..... .......... .......... .......... ..........
DBP_3736_Brazil-AF        .......... .......... .......... .......... .......... .......... .......... .......... .......... ..........
DBP_3739_Brazil-AF        .......... .......... .......... .......... ...T...... ....T..... .......... .......... .......... ..........
DBP_3746_Brazil-AF        .......... .......... .......... .......... .......... .......... .......... .......... .......... ..........
DBP_3750_Brazil-AF        .......... .......... .......... .......... .......... .......... .......... .......... .......... ..........
DBP_3916_Brazil-AF        .......... .......... .......... .......... .......... .......... .......... .......... .......... ..........
DBP_3931_Brazil-AF        .......... .......... .......... .......... ...T...... ....T..... .......... .......... .......... ..........
DBP_3962_Brazil-AF        .......... .......... .......... .......... .......... .......... .......... .......... .......... ..........
DBP_4083_Brazil-AF        .......... .......... .......... .......... ...T...... ....T..... .......... .......... .......... ..........
DBP_4096_Brazil-AF        .......... .......... .......... .......... .......... .......... .......... .......... .......... ..........
DBP_4097_Brazil-AF        .......... .......... .......... .......... ...T...... ....T..... .......... .......... .......... ..........
DBP_4112_Brazil-AF        .......... .......... .......... .......... .......... .......... .......... .......... .......... ..........
DBP_4118_Brazil-AF        .......... .......... .......... .......... ...T...... ....T..... .......... .......... .......... ..........
DBP_4131_Brazil-AF        .......... .......... .......... .......... .......... .......... .......... .......... .......... ..........
DBP_4161_Brazil-AF        .......... .......... .......... .......... .......... .......... .......... .......... .......... ..........

                                 110        120        130        140        150        160        170        180        190        200
                          ....|....| ....|....| ....|....| ....|....| ....|....| ....|....| ....|....| ....|....| ....|....| ....|....|
PVX_110810_|_Salvador 1   GATGCTGCAG TAGAGGGCGA TTTATTACTT AAGTTGAATA ACTACAGATA TAACAAAGAC TTTTGCAAGG ATATAAGATG GAGTTTGGGA GATTTTGGAG
DBP_07_Brazil-BA          .......... .......... .......... .......... .......... .......... .......... .......... .......... ..........
DBP_09_Brazil-BA          .......... .......... .......... .......... .......... .......... .......... .......... .......... ..........
DBP_10_Brazil-BA          .......... .......... .......... .......... .......... .......... .......... .......... .......... ..........
DBP_11_Brazil-BA          .......... .......... .......... .......... .......... .......... .......... .......... .......... ..........
DBP_12_Brazil-BA          .......... .......... .......... .......... .......... .......... .......... .......... .......... ..........
DBP_13_Brazil-BA          .......... .......... .......... .......... .......... .......... .......... .......... .......... ..........
DBP_14_Brazil-BA          .......... .......... .......... .......... .......... .......... .......... .......... .......... ..........
DBP_15_Brazil-BA          .......... .......... .......... .......... .......... .......... .......... .......... .......... ..........
DBP_16_Brazil-BA          .......... .......... .......... .......... .......... .......... .......... .......... .......... ..........
DBP_17_Brazil-BA          .......... .......... .......... .......... .......... .......... .......... .......... .......... ..........
DBP_18_Brazil-BA          .......... .......... .......... .......... .......... .......... .......... .......... .......... ..........
DBP_20_Brazil-BA          .......... .......... .......... .......... .......... .......... .......... .......... .......... ..........
DBP_21_Brazil-BA          .......... .......... .......... .......... .......... .......... .......... .......... .......... ..........
DBP_22_Brazil-BA          .......... .......... .......... .......... .......... .......... .......... .......... .......... ..........
DBP_24_Brazil-BA          .......... .......... .......... .......... .......... .......... .......... .......... .......... ..........
DBP_26_Brazil-BA          .......... .......... .......... .......... .......... .......... .......... .......... .......... ..........
DBP_27_Brazil-BA          .......... .......... .......... .......... .......... .......... .......... .......... .......... ..........
DBP_28_Brazil-BA          .......... .......... .......... .......... .......... .......... .......... .......... .......... ..........
DBP_29_Brazil-BA          .......... .......... .......... .......... .......... .......... .......... .......... .......... ..........
DBP_31_Brazil-BA          .......... .......... .......... .......... .......... .......... .......... .......... .......... ..........
DBP_32_Brazil-BA          .......... .......... .......... .......... .......... .......... .......... .......... .......... ..........
DBP_33_Brazil-BA          .......... .......... .......... .......... .......... .......... .......... .......... .......... ..........
DBP_35_Brazil-BA          .......... .......... .......... .......... .......... .......... .......... .......... .......... ..........
DBP_36_Brazil-BA          .......... .......... .......... .......... .......... .......... .......... .......... .......... ..........
DBP_37_Brazil-BA          .......... .......... .......... .......... .......... .......... .......... .......... .......... ..........
DBP_38_Brazil-BA          .......... .......... .......... .......... .......... .......... .......... .......... .......... ..........
DBP_39_Brazil-BA          .......... .......... .......... .......... .......... .......... .......... .......... .......... ..........
DBP_40_Brazil-BA          .......... .......... .......... .......... .......... .......... .......... .......... .......... ..........
DBP_41_Brazil-BA          .......... .......... .......... .......... .......... .......... .......... .......... .......... ..........
DBP_42_Brazil-BA          .......... .......... .......... .......... .......... .......... .......... .......... .......... ..........
DBP_43_Brazil-BA          .......... .......... .......... .......... .......... .......... .......... .......... .......... ..........
DBP_44_Brazil-BA          .......... .......... .......... .......... .......... .......... .......... .......... .......... ..........
```

DBP_45_Brazil-BA	....
DBP_46_Brazil-BA	....
DBP_47_Brazil-BA	....
DBP_48_Brazil-BA	....
DBP_49_Brazil-BA	....
DBP_51_Brazil-BA	....
DBP_53_Brazil-BA	....
DBP_54_Brazil-BA	....
DBP_55_Brazil-BA	....
DBP_56_Brazil-BA	....
DBP_57_Brazil-BA	....
DBP_58_Brazil-BA	....
DBP_59_Brazil-BA	....
DBP_1161_Brazil-BA	....
DBP_1182_Brazil-BA	....
DBP_1190_Brazil-BA	....
DBP_1200_Brazil-BA	....
DBP_1209_Brazil-BA	....T.. ....
DBP_1272_Brazil-BA	....
DBP_1295_Brazil-BA	....
DBP_1320_Brazil-BA	....
DBP_1327_Brazil-BA	....
DBP_1335_Brazil-BA	....
DBP_1340_Brazil-BA	....
DBP_1346_Brazil-BA	....
DBP_1382_Brazil-BA	....
DBP_1428_Brazil-BA	....T.. ....
DBP_1447_Brazil-BA	....
DBP_1469_Brazil-BA	....
DBP_1473_Brazil-BA	....
DBP_1475_Brazil-BA	....
DBP_1493_Brazil-BA	....
DBP_1532_Brazil-BA	....
DBP_1540_Brazil-BA	....
DBP_1621_Brazil-BA	....
DBP_1627_Brazil-BA	....
DBP_1641_Brazil-BA	....
DBP_1664_Brazil-BA	....
DBP_1741_Brazil-BA	....
DBP_1747_Brazil-BA	....
DBP_1748_Brazil-BA	....
DBP_1777_Brazil-BA	....
DBP_1789_Brazil-BA	....
DBP_1812_Brazil-BA	....
DBP_1824_Brazil-BA	....
DBP_1830_Brazil-BA	....
DBP_1837_Brazil-BA	....
DBP_1853_Brazil-BA	....
DBP_1867_Brazil-BA	....T.. ....
DBP_1914_Brazil-BA	....
DBP_1874_Brazil-BA	....

```
DBP_2036_Brazil-BA    .......... .......... .......... .......... .......... .......... .......... .......... .......... ..........
DBP_2159_Brazil-BA    .......... .......... .......... .......... .......... .......... .......... .......... .......... ..........
DBP_2212_Brazil-BA    .......... .......... .......... .......... .......... .......... .......... .......... .......... ..........
DBP_2272_Brazil-BA    .......... .......... .......... .......... .......... .......... .......... .......... .......... ..........
DBP_2327_Brazil-BA    .......... .......... .......T.. .......... .......... .......... .......... .......... .......... ..........
DBP_2339_Brazil-BA    .......... .......... .......... .......... .......... .......... .......... .......... .......... ..........
DBP_2452_Brazil-BA    .......... .......... .......... .......... .......... .......... .......... .......... .......... ..........
DBP_2462_Brazil-BA    .......... .......... .......... .......... .......... .......... .......... .......... .......... ..........
DBP_2517_Brazil-BA    .......... .......... .......... .......... .......... .......... .......... .......... .......... ..........
DBP_2592_Brazil-BA    .......... .......... .......... .......... .......... .......... .......... .......... .......... ..........
DBP_2600_Brazil-BA    .......... .......... .......... .......... .......... .......... .......... .......... .......... ..........
DBP_2650_Brazil-BA    .......... .......... .......... .......... .......... .......... .......... .......... .......... ..........
DBP_2688_Brazil-BA    .......... .......... .......... .......... .......... .......... .......... .......... .......... ..........
DBP_2695_Brazil-BA    .......... .......... .......... .......... .......... .......... .......... .......... .......... ..........
DBP_2692_Brazil-BA    .......... .......... .......... .......... .......... .......... .......... .......... .......... ..........
DBP_2693_Brazil-BA    .......... .......... .......... .......... .......... .......... .......... .......... .......... ..........
DBP_2705_Brazil-BA    .......... .......... .......... .......... .......... .......... .......... .......... .......... ..........
DBP_2737_Brazil-BA    .......... .......... .......... .......... .......... .......... .......... .......... .......... ..........
DBP_2751_Brazil-BA    .......... .......... .......... .......... .......... .......... .......... .......... .......... ..........
DBP_2773_Brazil-BA    .......... .......... .......... .......... .......... .......... .......... .......... .......... ..........
DBP_3124_Brazil-BA    .......... .......... .......... .......... .......... .......... .......... .......... .......... ..........
DBP_3164_Brazil-BA    .......... .......... .......... .......... .......... .......... .......... .......... .......... ..........
DBP_3260_Brazil-BA    .......... .......... .......... .......... .......... .......... .......... .......... .......... ..........
DBP_3280_Brazil-BA    .......... .......... .......... .......... .......... .......... .......... .......... .......... ..........
DBP_3307_Brazil-BA    .......... .......... .......... .......... .......... .......... .......... .......... .......... ..........
DBP_3311_Brazil-BA    .......... .......... .......... .......... .......... .......... .......... .......... .......... ..........
DBP_3314_Brazil-BA    .......... .......... .......... .......... .......... .......... .......... .......... .......... ..........
DBP_3340_Brazil-BA    .......... .......... .......... .......... .......... .......... .......... .......... .......... ..........
DBP_3404_Brazil-BA    .......... .......... .......... .......... .......... .......... .......... .......... .......... ..........
DBP_3507_Brazil-BA    .......... .......... .......... .......... .......... .......... .......... .......... .......... ..........
DBP_3516_Brazil-BA    .......... .......... .......... .......... .......... .......... .......... .......... .......... ..........
DBP_3530_Brazil-BA    .......... .......... .......T.. .......... .......... .......... .......... .......... .......... ..........
DBP_3556_Brazil-BA    .......... .......... .......... .......... .......... .......... .......... .......... .......... ..........
DBP_3560_Brazil-BA    .......... .......... .......... .......... .......... .......... .......... .......... .......... ..........
DBP_3569_Brazil-BA    .......... .......... .......... .......... .......... .......... .......... .......... .......... ..........
DBP_3601_Brazil-BA    .......... .......... .......... .......... .......... .......... .......... .......... .......... ..........
DBP_3617_Brazil-BA    .......... .......... .......... .......... .......... .......... .......... .......... .......... ..........
DBP_3661_Brazil-BA    .......... .......... .......T.. .......... .......... .......... .......... .......... .......... ..........
DBP_3707_Brazil-BA    .......... .......... .......... .......... .......... .......... .......... .......... .......... ..........
DBP_3814_Brazil-BA    .......... .......... .......... .......... .......... .......... .......... .......... .......... ..........
DBP_3842_Brazil-BA    .......... .......... .......T.. .......... .......... .......... .......... .......... .......... ..........
DBP_3876_Brazil-BA    .......... .......... .......... .......... .......... .......... .......... .......... .......... ..........
DBP_3880_Brazil-BA    .......... .......... .......... .......... .......... .......... .......... .......... .......... ..........
DBP_3908_Brazil-BA    .......... .......... .......... .......... .......... .......... .......... .......... .......... ..........
DBP_3913_Brazil-BA    .......... .......... .......... .......... .......... .......... .......... .......... .......... ..........
DBP_3914_Brazil-BA    .......... .......... .......... .......... .......... .......... .......... .......... .......... ..........
DBP_3937_Brazil-BA    .......... .......... .......... .......... .......... .......... .......... .......... .......... ..........
DBP_3974_Brazil-BA    .......... .......... .......... .......... .......... .......... .......... .......... .......... ..........
DBP_3976_Brazil-BA    .......... .......... .......... .......... .......... .......... .......... .......... .......... ..........
DBP_3998_Brazil-BA    .......... .......... .......... .......... .......... .......... .......... .......... .......... ..........
DBP_4030_Brazil-BA    .......... .......... .......... .......... .......... .......... .......... .......... .......... ..........
```

```
DBP_4071_Brazil-BA   .......... .......... .......... .......... .......... .......... .......... .......... .......... ..........
DBP_4109_Brazil-BA   .......... .......... .......... .......... .......... .......... .......... .......... .......... ..........
DBP_4135_Brazil-BA   .......... .......... .......... .......... .......... .......... .......... .......... .......... ..........
DBP_1274_Brazil-AF   .......... .......... .......... .......... .......... .......... .......... .......... .......... ..........
DBP_1347_Brazil-AF   .......... .......... .......... .......... .......... .......... .......... .......... .......... ..........
DBP_1436_Brazil-AF   .......... .......... .......... .......... .......... .......... .......... .......... .......... ..........
DBP_1737_Brazil-AF   .......... .......... .......... .......... .......... .......... .......... .......... .......... ..........
DBP_1652_Brazil-AF   .......... .......... .......T.. .......... .......... .......... .......... .......... .......... ..........
DBP_1745_Brazil-AF   .......... .......... .......... .......... .......... .......... .......... .......... .......... ..........
DBP_1856_Brazil-AF   .......... .......... .......T.. .......... .......... .......... .......... .......... .......... ..........
DBP_1906_Brazil-AF   .......... .......... .......... .......... .......... .......... .......... .......... .......... ..........
DBP_1948_Brazil-AF   .......... .......... .......T.. .......... .......... .......... .......... .......... .......... ..........
DBP_1960_Brazil-AF   .......... .......... .......... .......... .......... .......... .......... .......... .......... ..........
DBP_1961_Brazil-AF   .......... .......... .......T.. .......... .......... .......... .......... .......... .......... ..........
DBP_2728_Brazil-AF   .......... .......... .......T.. .......... .......... .......... .......... .......... .......... ..........
DBP_2734_Brazil-AF   .......... .......... .......... .......... .......... .......... .......... .......... .......... ..........
DBP_2736_Brazil-AF   .......... .......... .......T.. .......... .......... .......... .......... .......... .......... ..........
DBP_2747_Brazil-AF   .......... .......... .......T.. .......... .......... .......... .......... .......... .......... ..........
DBP_2752_Brazil-AF   .......... .......... .......T.. .......... .......... .......... .......... .......... .......... ..........
DBP_2762_Brazil-AF   .......... .......... .......T.. .......... .......... .......... .......... .......... .......... ..........
DBP_2765_Brazil-AF   .......... .......... .......T.. .......... .......... .......... .......... .......... .......... ..........
DBP_2769_Brazil-AF   .......... .......... .......T.. .......... .......... .......... .......... .......... .......... ..........
DBP_2770_Brazil-AF   .......... .......... .......T.. .......... .......... .......... .......... .......... .......... ..........
DBP_2775_Brazil-AF   .......... .......... .......T.. .......... .......... .......... .......... .......... .......... ..........
DBP_2779_Brazil-AF   .......... .......... .......T.. .......... .......... .......... .......... .......... .......... ..........
DBP_2782_Brazil-AF   .......... .......... .......T.. .......... .......... .......... .......... .......... .......... ..........
DBP_2792_Brazil-AF   .......... .......... .......T.. .......... .......... .......... .......... .......... .......... ..........
DBP_2793_Brazil-AF   .......... .......... .......T.. .......... .......... .......... .......... .......... .......... ..........
DBP_2816_Brazil-AF   .......... .......... .......T.. .......... .......... .......... .......... .......... .......... ..........
DBP_2840_Brazil-AF   .......... .......... .......T.. .......... .......... .......... .......... .......... .......... ..........
DBP_2859_Brazil-AF   .......... .......... .......T.. .......... .......... .......... .......... .......... .......... ..........
DBP_2900_Brazil-AF   .......... .......... .......T.. .......... .......... .......... .......... .......... .......... ..........
DBP_2916_Brazil-AF   .......... .......... .......T.. .......... .......... .......... .......... .......... .......... ..........
DBP_2923_Brazil-AF   .......... .......... .......T.. .......... .......... .......... .......... .......... .......... ..........
DBP_2980_Brazil-AF   .......... .......... .......... .......... .......... .......... .......... .......... .......... ..........
DBP_2982_Brazil-AF   .......... .......... .......T.. .......... .......... .......... .......... .......... .......... ..........
DBP_2983_Brazil-AF   .......... .......... .......... .......... .......... .......... .......... .......... .......... ..........
DBP_3071_Brazil-AF   .......... .......... .......T.. .......... .......... .......... .......... .......... .......... ..........
DBP_3079_Brazil-AF   .......... .......... .......T.. .......... .......... .......... .......... .......... .......... ..........
DBP_3084_Brazil-AF   .......... .......... .......T.. .......... .......... .......... .......... .......... .......... ..........
DBP_3089_Brazil-AF   .......... .......... .......T.. .......... .......... .......... .......... .......... .......... ..........
DBP_3157_Brazil-AF   .......... .......... .......... .......... .......... .......... .......... .......... .......... ..........
DBP_3276_Brazil-AF   .......... .......... .......... .......... .......... .......... .......... .......... .......... ..........
DBP_3277_Brazil-AF   .......... .......... .......T.. .......... .......... .......... .......... .......... .......... ..........
DBP_3278_Brazil-AF   .......... .......... .......T.. .......... .......... .......... .......... .......... .......... ..........
DBP_3284_Brazil-AF   .......... .......... .......... .......... .......... .......... .......... .......... .......... ..........
DBP_3296_Brazil-AF   .......... .......... .......T.. .......... .......... .......... .......... .......... .......... ..........
DBP_3300_Brazil-AF   .......... .......... .......T.. .......... .......... .......... .......... .......... .......... ..........
DBP_3310_Brazil-AF   .......... .......... .......T.. .......... .......... .......... .......... .......... .......... ..........
DBP_3315_Brazil-AF   .......... .......... .......T.. .......... .......... .......... .......... .......... .......... ..........
DBP_3325_Brazil-AF   .......... .......... .......... .......... .......... .......... .......... .......... .......... ..........
```

```
DBP_3334_Brazil-AF        .......... .......... .......T.. .......... .......... .......... .......... .......... .......... ..........
DBP_3371_Brazil-AF        .......... .......... .......T.. .......... .......... .......... .......... .......... .......... ..........
DBP_3376_Brazil-AF        .......... .......... .......T.. .......... .......... .......... .......... .......... .......... ..........
DBP_3379_Brazil-AF        .......... .......... .......T.. .......... .......... .......... .......... .......... .......... ..........
DBP_3406_Brazil-AF        .......... .......... .......T.. .......... .......... .......... .......... .......... .......... ..........
DBP_3612_Brazil-AF        .......... .......... .......T.. .......... .......... .......... .......... .......... .......... ..........
DBP_3613_Brazil-AF        .......... .......... .......T.. .......... .......... .......... .......... .......... .......... ..........
DBP_3614_Brazil-AF        .......... .......... .......T.. .......... .......... .......... .......... .......... .......... ..........
DBP_3616_Brazil-AF        .......... .......... .......T.. .......... .......... .......... .......... .......... .......... ..........
DBP_3630_Brazil-AF        .......... .......... .......T.. .......... .......... .......... .......... .......... .......... ..........
DBP_3633_Brazil-AF        .......... .......... .......T.. .......... .......... .......... .......... .......... .......... ..........
DBP_3643_Brazil-AF        .......... .......... .......T.. .......... .......... .......... .......... .......... .......... ..........
DBP_3659_Brazil-AF        .......... .......... .......... .......... .......... .......... .......... .......... .......... ..........
DBP_3698_Brazil-AF        .......... .......... .......T.. .......... .......... .......... .......... .......... .......... ..........
DBP_3705_Brazil-AF        .......... .......... .......... .......... .......... .......... .......... .......... .......... ..........
DBP_3722_Brazil-AF        .......... .......... .......T.. .......... .......... .......... .......... .......... .......... ..........
DBP_3735_Brazil-AF        .......... .......... .......T.. .......... .......... .......... .......... .......... .......... ..........
DBP_3736_Brazil-AF        .......... .......... .......... .......... .......... .......... .......... .......... .......... ..........
DBP_3739_Brazil-AF        .......... .......... .......T.. .......... .......... .......... .......... .......... .......... ..........
DBP_3746_Brazil-AF        .......... .......... .......... .......... .......... .......... .......... .......... .......... ..........
DBP_3750_Brazil-AF        .......... .......... .......... .......... .......... .......... .......... .......... .......... ..........
DBP_3916_Brazil-AF        .......... .......... .......... .......... .......... .......... .......... .......... .......... ..........
DBP_3931_Brazil-AF        .......... .......... .......T.. .......... .......... .......... .......... .......... .......... ..........
DBP_3962_Brazil-AF        .......... .......... .......... .......... .......... .......... .......... .......... .......... ..........
DBP_4083_Brazil-AF        .......... .......... .......... .......... .......... .......... .......... .......... .......... ..........
DBP_4096_Brazil-AF        .......... .......... .......... .......... .......... .......... .......... .......... .......... ..........
DBP_4097_Brazil-AF        .......... .......... .......T.. .......... .......... .......... .......... .......... .......... ..........
DBP_4112_Brazil-AF        .......... .......... .......... .......... .......... .......... .......... .......... .......... ..........
DBP_4118_Brazil-AF        .......... .......... .......... .......... .......... .......... .......... .......... .......... ..........
DBP_4131_Brazil-AF        .......... .......... .......... .......... .......... .......... .......... .......... .......... ..........
DBP_4161_Brazil-AF        .......... .......... .......... .......... .......... .......... .......... .......... .......... ..........

                                 210        220        230        240        250        260        270        280        290        300
                          ....|....| ....|....| ....|....| ....|....| ....|....| ....|....| ....|....| ....|....| ....|....| ....|....|
PVX_110810_|_Salvador 1   ATATAATTAT GGGAACGGAT ATGGAAGGCA TCGGATATTC CAAAGTAGTG GAAAATAATT TGCGCAGCAT CTTTGGAACT GATGAAAAGG CCCAACAGCG
DBP_07_Brazil-BA          .......... .......... .......... .......... .......... .......... ....T..... .......... .G.A...T.. .........A
DBP_09_Brazil-BA          .......... .......... .......... .......... .G........ .......... .......... .......... .G...C.... ..........
DBP_10_Brazil-BA          .......... .......... .......... .......... .......... .......... .......... .......... .......... .........A
DBP_11_Brazil-BA          .......... .......... .......... .......... .......... .......... .......... .......... .G........ .........A
DBP_12_Brazil-BA          .......... .......... .......... .......... .......... .......... ....T..... .......... .G.A...T.. .........A
DBP_13_Brazil-BA          .......... .......... .......... .......... .......... .......... .......... .......... .G........ .........A
DBP_14_Brazil-BA          .......... .......... .......... .......... .......... .......... .......... .......... .G........ .........A
DBP_15_Brazil-BA          .......... .......... .......... .......... .......... .......... .......... .......... .G........ ..........
DBP_16_Brazil-BA          .......... .......... .......... .......... .......... .......... ....T..... .......... .G.A...T.. .........A
DBP_17_Brazil-BA          .......... .......... .......... .......... .G........ .......... .......... .......... .G.A...T.. ..........
DBP_18_Brazil-BA          .......... .......... .......... .......... .......... .......... .......... .......... .G........ .........A
DBP_20_Brazil-BA          .......... .......... .......... .......... .......... .......... .......... .......... .G........ .........A
DBP_21_Brazil-BA          .......... .......... .......... .......... .......... .......... .......... .......... .......... ..........
DBP_22_Brazil-BA          .......... .......... .......... .......... .......... .......... .......... .......... .G........ .........A
DBP_24_Brazil-BA          .......... .......... .......... .......... .......... .......... .......... .......... .G...C.... ..........
DBP_26_Brazil-BA          .......... .......... .......... .......... .......... .......... .......... .......... .......... ..........
```

```
DBP_27_Brazil-BA      .......... .......... .......... .......... .......... .......... ....T..... .......... .G.A....T. .........A
DBP_28_Brazil-BA      .......... .......... .......... .......... .......... .......... .......... .......... .G........ .........A
DBP_29_Brazil-BA      .......... .......... .......... .......... .......... .......... .......... .......... .G........ .........A
DBP_31_Brazil-BA      .......... .......... .......... .......... .G........ .......... .......... .......... .G........ ..........
DBP_32_Brazil-BA      .......... .......... .......... .......... .G........ .......... .......... .......... .G....C... ..........
DBP_33_Brazil-BA      .......... .......... .......... .......... .......... .......... .......... .......... .G........ .........A
DBP_35_Brazil-BA      .......... .......... .......... .......... .......... .......... .......... .......... .G........ .........A
DBP_36_Brazil-BA      .......... .......... .......... .......... .......... .......... .......... .......... .G........ ..........
DBP_37_Brazil-BA      .......... .......... .......... .......... .......... .......... .......... .......... .......... ..........
DBP_38_Brazil-BA      .......... .......... .......... .......... .......... .......... .......... .......... .G........ ..........
DBP_39_Brazil-BA      .......... .......... .......... .......... .......... .......... .......... .......... .G........ .........A
DBP_40_Brazil-BA      .......... .......... .......... .......... .G........ .......... .......... .......... .G....C... ..........
DBP_41_Brazil-BA      .......... .......... .......... .......... .......... .......... .......... .......... .G........ .........A
DBP_42_Brazil-BA      .......... .......... .......... .......... .......... .......... .......... .......... .G........ .........A
DBP_43_Brazil-BA      .......... .......... .......... .......... .......... .......... .......... .......... .......... ..........
DBP_44_Brazil-BA      .......... .......... .......... .......... .......... .......... .......... .......... .G........ .........A
DBP_45_Brazil-BA      .......... .......... .......... .......... .......... .......... .......... .......... .G........ .........A
DBP_46_Brazil-BA      .......... .......... .......... .......... .......... .......... .......... .......... .G........ .........A
DBP_47_Brazil-BA      .......... .......... .......... .......... .......... .......... .......... .......... ......C... ..........
DBP_48_Brazil-BA      .......... .......... .......... .......... .......... .......... .......... .......... .G........ .........A
DBP_49_Brazil-BA      .......... .......... .......... .......... .G........ .......... .......... .......... .G....C... ..........
DBP_51_Brazil-BA      .......... .......... .......... .......... .......... .......... .......... .......... .......... ..........
DBP_53_Brazil-BA      .......... .......... .......... .......... .......... .......... .......... .......... .......... ..........
DBP_54_Brazil-BA      .......... .......... .......... .......... .......... .......... .......... .......... .G........ .........A
DBP_55_Brazil-BA      .......... .......... .......... .......... .......... .......... .......... .......... .G........ ..........
DBP_56_Brazil-BA      .......... .......... .......... .......... .......... .......... .......... .......... .G....C... ..........
DBP_57_Brazil-BA      .......... .......... .......... .......... .G........ .......... .......... .......... .G....C... ..........
DBP_58_Brazil-BA      .......... .......... .......... .......... .G........ .......... .......... .......... .G....C... ..........
DBP_59_Brazil-BA      .......... .......... .......... .......... .G........ .......... .......... .......... .G....C... ..........
DBP_1161_Brazil-BA    .......... .......... .......... .......... .G........ .......... .......... .......... .G....C... ..........
DBP_1182_Brazil-BA    .......... .......... .......... .......... .G........ .......... .......... .......... .G........ .........A
DBP_1190_Brazil-BA    .......... .......... .......... .......... .G........ .......... .......... .......... .G........ ..........
DBP_1200_Brazil-BA    .......... .......... .......... .......... .G........ .......... .......... .......... .G....C... ..........
DBP_1209_Brazil-BA    .......... .......... .......... .......... .......... ...G...... ....T..... .......... ...A..C... .........A
DBP_1272_Brazil-BA    .......... .......... .......... .......... .G........ .......... .......... .......... .G........ .........A
DBP_1295_Brazil-BA    .......... .......... .......... .......... .G........ .......... .......... .......... .G........ ..........
DBP_1320_Brazil-BA    .......... .......... .......... .......... .......... .......... .......... .......... .......... ..........
DBP_1327_Brazil-BA    .......... .......... .......... .......... .......... .......... .......... .......... .G........ .........A
DBP_1335_Brazil-BA    .......... .......... .......... .......... .......... .......... .......... .......... .G........ .........A
DBP_1340_Brazil-BA    .......... .......... .......... .......... .G........ .......... .......... .......... .G.A....T. ..........
DBP_1346_Brazil-BA    .......... .......... .......... .......... .G........ .......... .......... .......... .G....C... ..........
DBP_1382_Brazil-BA    .......... .......... .......... .......... .G........ .......... .......... .......... .G........ ..........
DBP_1428_Brazil-BA    .......... .......... .......... .......... .......... ...G...... .......... .......... .G.A....T. .........A
DBP_1447_Brazil-BA    .......... .......... .......... .......... .G........ .......... .......... .......... .G....C... ..........
DBP_1469_Brazil-BA    .......... .......... .......... .......... .......... .......... .......... .......... .G.A....T. ..........
DBP_1473_Brazil-BA    .......... .......... .......... .......... .......... .......... .......... .......... .G.A....T. .........A
DBP_1475_Brazil-BA    .......... .......... .......... .......... .G........ .......... .......... .......... .G....C... ..........
DBP_1493_Brazil-BA    .......... .......... .......... .......... .......... .......... ....T..... .......... .G.A....T. .........A
DBP_1532_Brazil-BA    .......... .......... .......... .......... .G........ .......... .......... .......... .G....C... ..........
DBP_1540_Brazil-BA    .......... .......... .......... .......... .......... .......... .......... .......... .G........ .........A
DBP_1621_Brazil-BA    .......... .......... .......... .......... .G........ .......... .......... .......... .G....C... ..........
```

```
DBP_1627_Brazil-BA      .......... .......... .......... .......... .G........ .......... .......... .......... .G....C... ..........
DBP_1641_Brazil-BA      .......... .......... .......... .......... .......... .......... .......... .......... .......... .........A
DBP_1664_Brazil-BA      .......... .......... .......... .......... .......... .......... .......... .......... .G........ .........A
DBP_1741_Brazil-BA      .......... .......... .......... .......... .G........ .......... .......... .......... .G........ ..........
DBP_1747_Brazil-BA      .......... .......... .......... .......... .......... .......... .......... .......... .......... ..........
DBP_1748_Brazil-BA      .......... .......... .......... .......... .......... .......... .......... .......... .G........ .........A
DBP_1777_Brazil-BA      .......... .......... .......... .......... .......... .......... .......... .......... .......... ..........
DBP_1789_Brazil-BA      .......... .......... .......... .......... .......... .......... .......... .......... .G........ .........A
DBP_1812_Brazil-BA      .......... .......... .......... .......... .G........ .......... .......... .......... .G........ ..........
DBP_1824_Brazil-BA      .......... .......... .......... .......... .......... .......... .......... .......... .......... ..........
DBP_1830_Brazil-BA      .......... .......... .......... .......... .G........ .......... .......... .......... .G.A....T. ..........
DBP_1837_Brazil-BA      .......... .......... .......... .......... .G........ .......... .......... .......... .G....C... ..........
DBP_1853_Brazil-BA      .......... .......... .......... .......... .G........ .......... .......... .......... .G....C... ..........
DBP_1867_Brazil-BA      .......... .......... .......... .......... .G........ .......... .......... .......... .G....C... ..........
DBP_1914_Brazil-BA      .......... .......... .......... .......... .......... .......... ....T..... .......... .G.A....T. .........A
DBP_1874_Brazil-BA      .......... .......... .......... .......... .......... .......... .......... .......... .G........ ..........
DBP_2036_Brazil-BA      .......... .......... .......... .......... .......... .......... .......... .......... .G........ ..........
DBP_2159_Brazil-BA      .......... .......... .......... .......... .......... .......... .......... .......... .......... ..........
DBP_2212_Brazil-BA      .......... .......... .......... .......... .......... ...G...... .......... .......... .......... ..........
DBP_2272_Brazil-BA      .......... .......... .......... .......... .......... .......... .......... .......... .......... ..........
DBP_2327_Brazil-BA      .......... .......... .......... .......... .......... .......... .......... .......... .G.A..C... .........A
DBP_2339_Brazil-BA      .......... .......... .......... .......... .......... .......... .......... .......... .G........ ..........
DBP_2452_Brazil-BA      .......... .......... .......... .......... .G........ .......... .......... .......... .G.C....T. ..........
DBP_2462_Brazil-BA      .......... .......... .......... .......... .......... .......... .......... .......... .......... ..........
DBP_2517_Brazil-BA      .......... .......... .......... .......... .G........ .......... ....T..... .......... .G........ .........A
DBP_2592_Brazil-BA      .......... .......... .......... .......... .G........ .......... .......... .......... .G........ .........A
DBP_2600_Brazil-BA      .......... .......... .......... .......... .G........ .......... .......... .......... .G........ .........A
DBP_2650_Brazil-BA      .......... .......... .......... .......... .G........ .......... .......... .......... .G........ .........A
DBP_2688_Brazil-BA      .......... .......... .......... .......... .G........ .......... .......... .......... .G....C... ..........
DBP_2695_Brazil-BA      .......... .......... .......... .......... .......... .......... .......... .......... .G........ ..........
DBP_2692_Brazil-BA      .......... .......... .......... .......... .......... .......... .......... .......... .G........ ..........
DBP_2693_Brazil-BA      .......... .......... .......... .......... .G........ .......... .......... .......... .G.A....T. ..........
DBP_2705_Brazil-BA      .......... .......... .......... .......... .......... .......... .......... .......... .G........ .........A
DBP_2737_Brazil-BA      .......... .......... .......... .......... .......... .......... .......... .......... .......... ..........
DBP_2751_Brazil-BA      .......... .......... .......... .......... .......... .......... .......... .......... .G........ .........A
DBP_2773_Brazil-BA      .......... .......... .......... .......... .G........ .......... .......... .......... .G....C... ..........
DBP_3124_Brazil-BA      .......... .......... .......... .......... .......... .......... .......... .......... .G........ .........A
DBP_3164_Brazil-BA      .......... .......... .......... .......... .G........ .......... .......... .......... .G.A....T. ..........
DBP_3260_Brazil-BA      .......... .......... .......... .......... .......... .......... .......... .......... .G.A....T. ..........
DBP_3280_Brazil-BA      .......... .......... .......... .......... .G........ .......... .......... .......... .G.A....T. ..........
DBP_3307_Brazil-BA      .......... .......... .......... .......... .G........ .......... .......... .......... .G....C... ..........
DBP_3311_Brazil-BA      .......... .......... .......... .......... .G........ .......... .......... .......... .G.A....T. ..........
DBP_3314_Brazil-BA      .......... .......... .......... .......... .G........ .......... .......... .......... .G....C... ..........
DBP_3340_Brazil-BA      .......... .......... .......... .......... .G........ .......... .......... .......... .G.A....T. ..........
DBP_3404_Brazil-BA      .......... .......... .......... .......... .G........ .......... .......... .......... .G.A....T. ..........
DBP_3507_Brazil-BA      .......... .......... .......... .......... .G........ .......... .......... .......... .G........ ..........
DBP_3516_Brazil-BA      .......... .......... .......... .......... .......... .......... .......... .......... .G........ .........A
DBP_3530_Brazil-BA      .......... .......... .......... .......... .......... ...G...... ....T..... .......... .G.A....T. .........A
DBP_3556_Brazil-BA      .......... .......... .......... .......... .......... .......... .......... .......... .G........ .........A
DBP_3560_Brazil-BA      .......... .......... .......... .......... .G........ .......... .......... .......... .G........ ..........
DBP_3569_Brazil-BA      .......... .......... .......... .......... .......... .......... .......... .......... .G........ .........A
```

```
DBP_3601_Brazil-BA   .......... .......... .......... .......... .......... .......... .......... .......... .......... ..........
DBP_3617_Brazil-BA   .......... .......... .......... .......... .G........ .......... .......... .......... .G........ ..........
DBP_3661_Brazil-BA   .......... .......... .......... .......... .......... ...G...... ....T..... .......... .G.A....T. .........A
DBP_3707_Brazil-BA   .......... .......... .......... .......... .......... .......... .......... .......... .G........ .........A
DBP_3814_Brazil-BA   .......... .......... .......... .......... .......... .......... .......... .......... ........T. ..........
DBP_3842_Brazil-BA   .......... .......... .......... .......... .G........ .......... ....T..... .......... .G.A....T. .........A
DBP_3876_Brazil-BA   .......... .......... .......... .......... .G........ .......... .......... .......... .G........ ..........
DBP_3880_Brazil-BA   .......... .......... .......... .......... .......... ...G...... .......... .......... ......C... .........A
DBP_3908_Brazil-BA   .......... .......... .......... .......... .G........ .......... .......... .......... .G....C... ..........
DBP_3913_Brazil-BA   .......... .......... .......... .......... .G........ .......... .......... .......... .G.A....T. ..........
DBP_3914_Brazil-BA   .......... .......... .......... .......... .......... .......... .......... .......... .......... ..........
DBP_3937_Brazil-BA   .......... .......... .......... .......... .G........ .......... .......... .......... .G.A....T. ..........
DBP_3974_Brazil-BA   .......... .......... .......... .......... .G........ .......... .......... .......... .G.A....T. ..........
DBP_3976_Brazil-BA   .......... .......... .......... .......... .G........ .......... .......... .......... .G.A....T. ..........
DBP_3998_Brazil-BA   .......... .......... .......... .......... .G........ .......... .......... .......... .G....C... ..........
DBP_4030_Brazil-BA   .......... .......... .......... .......... .......... .......... .......... .......... .G.A...... ..........
DBP_4071_Brazil-BA   .......... .......... .......... .......... .......... .......... .......... .......... .......... ..........
DBP_4109_Brazil-BA   .......... .......... .......... .......... .......... .......... .......... .......... .......... ..........
DBP_4135_Brazil-BA   .......... .......... .......... .......... .......... .......... ....T..... .......... .G.A....T. .........A
DBP_1274_Brazil-AF   .......... .......... .......... .......... .G........ .......... .......... .......... .G....C... ..........
DBP_1347_Brazil-AF   .......... .......... .......... .......... .......... .......... .......... .......... .......... ..........
DBP_1436_Brazil-AF   .......... .......... .......... .......... .......... .......... ....T..... .......... .G.A....T. .........A
DBP_1737_Brazil-AF   .......... .......... .......... .......... .G........ .......... .......... .......... .G....C... ..........
DBP_1652_Brazil-AF   .......... .......... .......... .......... ...T...... .......... ....T..... .......... .G.C....T. .........A
DBP_1745_Brazil-AF   .......... .......... .......... .......... .G........ .......... .......... .......... .G....C... ..........
DBP_1856_Brazil-AF   .......... .......... .......... .......... .......... .......... .......... .......... .G.A...CT. .........A
DBP_1906_Brazil-AF   .......... .......... .......... .......... .......... .......... .......... .......... .G.A...CT. .........A
DBP_1948_Brazil-AF   .......... .......... .......... .......... .......... .......... ....T..... .......... .G.C....T. .........A
DBP_1960_Brazil-AF   .......... .......... .......... .......... .......... .......... ....T..... .......... .G.AC...T. .........A
DBP_1961_Brazil-AF   .......... .......... .......... .......... ...T...... .......... ....T..... .......... .G.C....T. .........A
DBP_2728_Brazil-AF   .......... .......... .......... .......... .......... .......... ....T..... .......... .G.A...CT. .........A
DBP_2734_Brazil-AF   .......... .......... .......... .......... .G........ .......... .......... .......... .G....C... ..........
DBP_2736_Brazil-AF   .......... .......... .......... .......... .......... .......... ....T..... .......... .G.A...... .........A
DBP_2747_Brazil-AF   .......... .......... .......... .......... .......... .......... ....T..... .......... .G.A...CT. .........A
DBP_2752_Brazil-AF   .......... .......... .......... .......... .......... .......... ....T..... .......... .G.A...CT. .........A
DBP_2762_Brazil-AF   .......... .......... .......... .......... .......... .......... ....T..... .......... .G.A...CT. .........A
DBP_2765_Brazil-AF   .......... .......... .......... .......... ...T...... .......... ....T..... .......... .G.C....T. .........A
DBP_2769_Brazil-AF   .......... .......... .......... .......... .......... .......... ....T..... .......... .G.A....T. .........A
DBP_2770_Brazil-AF   .......... .......... .......... .......... .......... .......... ....T..... .......... .G.A...CT. .........A
DBP_2775_Brazil-AF   .......... .......... .......... .......... .......... .......... ....T..... .......... .G.C....T. .........A
DBP_2779_Brazil-AF   .......... .......... .......... .......... .......... .......... ....T..... .......... .G.A...CT. .........A
DBP_2782_Brazil-AF   .......... .......... .......... .......... .......... .......... ....T..... .......... .G.A....T. .........A
DBP_2792_Brazil-AF   .......... .......... .......... .......... ...T...... .......... ....T..... .......... .G.C....T. .........A
DBP_2793_Brazil-AF   .......... .......... .......... .......... ...T...... .......... ....T..... .......... .G.C....T. .........A
DBP_2816_Brazil-AF   .......... .......... .......... .......... .......... .......... ....T..... .......... .G.C....T. .........A
DBP_2840_Brazil-AF   .......... .......... .......... .......... .......... .......... ....T..... .......... .G.A....T. .........A
DBP_2859_Brazil-AF   .......... .......... .......... .......... .......... .......... ....T..... .......... .G.A...CT. .........A
DBP_2900_Brazil-AF   .......... .......... .......... .......... .......... .......... ....T..... .......... .G.A....T. .........A
DBP_2916_Brazil-AF   .......... .......... .......... .......... .......... .......... ....T..... .......... .G.C....T. .........A
DBP_2923_Brazil-AF   .......... .......... .......... .......... .......... .......... ....T..... .......... .G.A....T. .........A
DBP_2980_Brazil-AF   .......... .......... .......... .......... .G........ .......... .......... .......... .G....C... ..........
```

```
DBP_2982_Brazil-AF    .......... .......... .......... .......... ...T...... .......... ....T..... .......... .G.C....T. .........A
DBP_2983_Brazil-AF    .......... .......... .......... .......... .G........ .......... .......... .......... .G....C... ..........
DBP_3071_Brazil-AF    .......... .......... .......... .......... .......... .......... ....T..... .......... .G.C....T. .........A
DBP_3079_Brazil-AF    .......... .......... .......... .......... .......... .......... ....T..... .......... .G.C....T. .........A
DBP_3084_Brazil-AF    .......... .......... .......... .......... .......... .......... ....T..... .......... .G.C....T. .........A
DBP_3089_Brazil-AF    .......... .......... .......... .......... .......... .......... ....T..... .......... .G.A...CT. .........A
DBP_3157_Brazil-AF    .......... .......... .......... .......... .G........ .......... .......... .......... .G....C... ..........
DBP_3276_Brazil-AF    .......... .......... .......... .......... .G........ .......... .......... .......... .G....C... ..........
DBP_3277_Brazil-AF    .......... .......... .......... .......... .......... .......... ....T..... .......... .G.A....T. .........A
DBP_3278_Brazil-AF    .......... .......... .......... .......... ...T...... .......... ....T..... .......... .G.C....T. .........A
DBP_3284_Brazil-AF    .......... .......... .......... .......... .......... .......... ....T..... .......... .G.A....T. .........A
DBP_3296_Brazil-AF    .......... .......... .......... .......... .......... .......... ....T..... .......... .G.A...CT. .........A
DBP_3300_Brazil-AF    .......... .......... .......... .......... .......... .......... ....T..... .......... .G.A...CT. .........A
DBP_3310_Brazil-AF    .......... .......... .......... .......... .......... .......... ....T..... .......... .G.A...CT. .........A
DBP_3315_Brazil-AF    .......... .......... .......... .......... .......... .......... ....T..... .......... .G.A...CT. .........A
DBP_3325_Brazil-AF    .......... .......... .......... .......... .G........ .......... .......... .......... .G....C... ..........
DBP_3334_Brazil-AF    .......... .......... .......... .......... ...T...... .......... ....T..... .......... .G.C....T. .........A
DBP_3371_Brazil-AF    .......... .......... .......... .......... ...T...... .......... ....T..... .......... .G.C....T. .........A
DBP_3376_Brazil-AF    .......... .......... .......... .......... .......... .......... ....T..... .......... .G.C....T. .........A
DBP_3379_Brazil-AF    .......... .......... .......... .......... .......... .......... ....T..... .......... .G.C....T. .........A
DBP_3406_Brazil-AF    .......... .......... .......... .......... .......... .......... .......... .......... .G.A...CT. .........A
DBP_3612_Brazil-AF    .......... .......... .......... .......... .......... .......... ....T..... .......... .G.A...CT. .........A
DBP_3613_Brazil-AF    .......... .......... .......... .......... .......... .......... ....T..... .......... .G.A...CT. .........A
DBP_3614_Brazil-AF    .......... .......... .......... .......... .......... .......... ....T..... .......... .G.C....T. .........A
DBP_3616_Brazil-AF    .......... .......... .......... .......... ...T...... .......... ....T..... .......... .G.C....T. .........A
DBP_3630_Brazil-AF    .......... .......... .......... .......... .......... .......... ....T..... .......... .G.A...CT. .........A
DBP_3633_Brazil-AF    .......... .......... .......... .......... .......... .......... ....T..... .......... .G.C....T. .........A
DBP_3643_Brazil-AF    .......... .......... .......... .......... .......... .......... ....T..... .......... .G.A....T. .........A
DBP_3659_Brazil-AF    .......... .......... .......... .......... .......... .......... ....T..... .......... .G.A....T. .........A
DBP_3698_Brazil-AF    .......... .......... .......... .......... .......... .......... .......... .......... .G.A....T. ..........
DBP_3705_Brazil-AF    .......... .......... .......... .......... .......... .......... .......... .......... .G........ .........A
DBP_3722_Brazil-AF    .......... .......... .......... .......... .......... .......... ....T..... .......... .G.A...CT. .........A
DBP_3735_Brazil-AF    .......... .......... .......... .......... .......... .......... ....T..... .......... .G.A...CT. .........A
DBP_3736_Brazil-AF    .......... .......... .......... .......... .G........ .......... .......... .......... .G....C... ..........
DBP_3739_Brazil-AF    .......... .......... .......... .......... .......... .......... ....T..... .......... .G.A...CT. .........A
DBP_3746_Brazil-AF    .......... .......... .......... .......... .G........ .......... .......... .......... .G....C... ..........
DBP_3750_Brazil-AF    .......... .......... .......... .......... .G........ .......... .......... .......... .G....C... ..........
DBP_3916_Brazil-AF    .......... .......... .......... .......... .G........ .......... .......... .......... .G....C... ..........
DBP_3931_Brazil-AF    .......... .......... .......... .......... .......... .......... ....T..... .......... .G.A....T. .........A
DBP_3962_Brazil-AF    .......... .......... .......... .......... .......... .......... .......... .......... .G.A....T. ..........
DBP_4083_Brazil-AF    .......... .......... .......... .......... .......... .......... ....T..... .......... .G.AC...T. .........A
DBP_4096_Brazil-AF    .......... .......... .......... .......... .G........ .......... .......... .......... .G....C... ..........
DBP_4097_Brazil-AF    .......... .......... .......... .......... .......... .......... ....T..... .......... .G.A...CT. .........A
DBP_4112_Brazil-AF    .......... .......... .......... .......... .G........ .......... .......... .......... .G....C... ..........
DBP_4118_Brazil-AF    .......... .......... .......... .......... .......... .......... ....T..... .......... .G.AC...T. .........A
DBP_4131_Brazil-AF    .......... .......... .......... .......... .G........ .......... .......... .......... .G....C... ..........
DBP_4161_Brazil-AF    .......... .......... .......... .......... .G........ .......... .......... .......... .G....C... ..........
```

```
                                     310        320        330        340        350        360        370        380        390        400
                             ....|....| ....|....| ....|....| ....|....| ....|....| ....|....| ....|....| ....|....| ....|....| ....|....|
PVX_110810_|_Salvador 1      TCGTAAACAG TGGTGGAATG AATCTAAAGC ACAAATTTGG ACAGCAATGA TGTACTCAGT TAAAAAAAGA TTAAAGGGGA ATTTTATATG GATTTGTAAA
DBP_07_Brazil-BA             .......... .......... ..A....... .......... .G........ .......... .......... .......... .......... ..........
DBP_09_Brazil-BA             .......... .......... .......... .......... .......... .......... .......... .......... .A........ ..........
DBP_10_Brazil-BA             .......... .......... .......... .......... .......... .......... .......... .......... .......... ..........
DBP_11_Brazil-BA             .......... .......... .......... .......... .......... .......... .......... .......... .......... ..........
DBP_12_Brazil-BA             .......... .......... ..A....... .......... .G........ .......... .......... .......... .......... ..........
DBP_13_Brazil-BA             .......... .......... .......... .......... .......... .......... .......... .......... .......... ..........
DBP_14_Brazil-BA             .......... .......... .......... .......... .......... .......... .......... .......... .......... ..........
DBP_15_Brazil-BA             .......... .......... .......... .......... .......... .......... .......... .......... .A........ ..........
DBP_16_Brazil-BA             .......... .......... ..A....... .......... .G........ .......... .......... .......... .......... ..........
DBP_17_Brazil-BA             .......... .......... .......... .......... .......... .......... .......... .......... .A........ ..........
DBP_18_Brazil-BA             .......... .......... .......... .......... .......... .......... .......... .......... .......... ..........
DBP_20_Brazil-BA             .......... .......... .......... .......... .......... .......... .......... .......... .......... ..........
DBP_21_Brazil-BA             .......... .......... .......... .......... .......... .......... .......... .......... .......... ..........
DBP_22_Brazil-BA             .......... .......... .......... .......... .......... .......... .......... .......... .......... ..........
DBP_24_Brazil-BA             .......... .......... .......... .......... .......... .......... .......... .......... .A........ ..........
DBP_26_Brazil-BA             .......... .......... .......... .......... .......... .......... .......... .......... .A........ ..........
DBP_27_Brazil-BA             .......... .......... ..A....... .......... .G........ .......... .......... .......... .......... ..........
DBP_28_Brazil-BA             .......... .......... .......... .......... .......... .......... .......... .......... .......... ..........
DBP_29_Brazil-BA             .......... .......... .......... .......... .......... .......... .......... .......... .......... ..........
DBP_31_Brazil-BA             .......... .......... .......... .......... .......... .......... .......... .......... .A........ ..........
DBP_32_Brazil-BA             .......... .......... .......... .......... .......... .......... .......... .......... .A........ ..........
DBP_33_Brazil-BA             .......... .......... .......... .......... .......... .......... .......... .......... .......... ..........
DBP_35_Brazil-BA             .......... .......... .......... .......... .......... .......... .......... .......... .......... ..........
DBP_36_Brazil-BA             .......... .......... .......... .......... .......... .......... .......... .......... .......... ..........
DBP_37_Brazil-BA             .......... .......... .......... .......... .......... .......... .......... .......... .......... ..........
DBP_38_Brazil-BA             .......... .......... .......... .......... .......... .......... .......... .......... .......... ..........
DBP_39_Brazil-BA             .......... .......... .......... .......... .......... .......... .......... .......... .......... ..........
DBP_40_Brazil-BA             .......... .......... .......... .......... .......... .......... .......... .......... .A........ ..........
DBP_41_Brazil-BA             .......... .......... .......... .......... .......... .......... .......... .......... .......... ..........
DBP_42_Brazil-BA             .......... .......... .......... .......... .......... .......... .......... .......... .......... ..........
DBP_43_Brazil-BA             .......... .......... .......... .......... .......... .......... .......... .......... .......... ..........
DBP_44_Brazil-BA             .......... .......... .......... .......... .......... .......... .......... .......... .......... ..........
DBP_45_Brazil-BA             .......... .......... .......... .......... .......... .......... .......... .......... .......... ..........
DBP_46_Brazil-BA             .......... .......... .......... .......... .......... .......... .......... .......... .......... ..........
DBP_47_Brazil-BA             .......... .......... .......... .......... .......... .......... .......... .......... .A........ ..........
DBP_48_Brazil-BA             .......... .......... .......... .......... .......... .......... .......... .......... .......... ..........
DBP_49_Brazil-BA             .......... .......... .......... .......... .......... .......... .......... .......... .A........ ..........
DBP_51_Brazil-BA             .......... .......... .......... .......... .......... .......... .......... .......... .A........ ..........
DBP_53_Brazil-BA             .......... .......... .......... .......... .......... .......... .......... .......... .A........ ..........
DBP_54_Brazil-BA             .......... .......... .......... .......... .......... .......... .......... .......... .......... ..........
DBP_55_Brazil-BA             .......... .......... .......... .......... .......... .......... .......... .......... .......... ..........
DBP_56_Brazil-BA             .......... .......... .......... .......... .......... .......... .......... .......... .......... ..........
DBP_57_Brazil-BA             .......... .......... .......... .......... .......... .......... .......... .......... .A........ ..........
DBP_58_Brazil-BA             .......... .......... .......... .......... .......... .......... .......... .......... .A........ ..........
DBP_59_Brazil-BA             .......... .......... .......... .......... .......... .......... .......... .......... .A........ ..........
DBP_1161_Brazil-BA           .......... .......... .......... .......... .......... .......... .......... .......... .A........ ..........
DBP_1182_Brazil-BA           .......... .......... .......... .......... .......... .......... .......... .......... .......... ..........
DBP_1190_Brazil-BA           .......... .......... .......... .......... .......... .......... .......... .......... .A.....G.. ..........
```

```
DBP_1200_Brazil-BA   .......... .......... .......... .......... .......... .......... .......... .......... .......... ..........
DBP_1209_Brazil-BA   .......... .......... .......... .......... .......... .......... .......... .......... .A........ ..........
DBP_1272_Brazil-BA   .......... .......... .......... .......... .......... .......... .......... .......... .......... ..........
DBP_1295_Brazil-BA   .......... .......... .......... .......... .......... .......... .......... .......... .A.....G.. ..........
DBP_1320_Brazil-BA   .......... .......... .......... .......... .......... .......... .......... .......... .......... ..........
DBP_1327_Brazil-BA   .......... .......... .......... .......... .......... .......... .......... .......... .......... ..........
DBP_1335_Brazil-BA   .......... .......... .......... .......... .......... .......... .......... .......... .......... ..........
DBP_1340_Brazil-BA   .......... .......... .......... .......... .......... .......... .......... .......... .A........ ..........
DBP_1346_Brazil-BA   .......... .......... .......... .......... .......... .......... .......... .......... .A........ ..........
DBP_1382_Brazil-BA   .......... .......... .......... .......... .......... .......... .......... .......... .A.....G.. ..........
DBP_1428_Brazil-BA   .......... .......... .......... .......... .......... .......... .......... .......... .A........ ..........
DBP_1447_Brazil-BA   .......... .......... .......... .......... .......... .......... .......... .......... .A........ ..........
DBP_1469_Brazil-BA   .......... .......... .......... .......... .......... .......... .......... .......... .......... ..........
DBP_1473_Brazil-BA   .......... .......... .......... .......... .......... .......... .......... .......... .......G.. ..........
DBP_1475_Brazil-BA   .......... .......... .......... .......... .......... .......... .......... .......... .A........ ..........
DBP_1493_Brazil-BA   .......... .......... .......... .......... .......... .......... .......... .......... .......... ..........
DBP_1532_Brazil-BA   .......... .......... .......... .......... .......... .......... .......... .......... .A........ ..........
DBP_1540_Brazil-BA   .......... .......... .......... .......... .......... .......... .......... .......... .......... ..........
DBP_1621_Brazil-BA   .......... .......... .......... .......... .......... .......... .......... .......... .A........ ..........
DBP_1627_Brazil-BA   .......... .......... .......... .......... .......... .......... .......... .......... .A........ ..........
DBP_1641_Brazil-BA   .......... .......... .......... .......... .......... .......... .......... .......... .......... ..........
DBP_1664_Brazil-BA   .......... .......... .......... .......... .......... .......... .......... .......... .......... ..........
DBP_1741_Brazil-BA   .......... .......... .......... .......... .......... .......... .......... .......... .A.....G.. ..........
DBP_1747_Brazil-BA   .......... .......... .......... .......... .......... .......... .......... .......... .......... ..........
DBP_1748_Brazil-BA   .......... .......... .......... .......... .......... .......... .......... .......... .......... ..........
DBP_1777_Brazil-BA   .......... .......... .......... .......... .......... .......... .......... .......... .......... ..........
DBP_1789_Brazil-BA   .......... .......... .......... .......... .......... .......... .......... .......... .......... ..........
DBP_1812_Brazil-BA   .......... .......... .......... .......... .......... .......... .......... .......... .A.....G.. ..........
DBP_1824_Brazil-BA   .......... .......... .......... .......... .......... .......... .......... .......... .A........ ..........
DBP_1830_Brazil-BA   .......... .......... .......... .......... .......... .......... .......... .......... .A........ ..........
DBP_1837_Brazil-BA   .......... .......... .......... .......... .......... .......... .......... .......... .A........ ..........
DBP_1853_Brazil-BA   .......... .......... .......... .......... .......... .......... .......... .......... .A........ ..........
DBP_1867_Brazil-BA   .......... .......... .......... .......... .......... .......... .......... .......... .A........ ..........
DBP_1914_Brazil-BA   .......... .......... ..A....... .......... .G........ .......... .......... .......... .......... ..........
DBP_1874_Brazil-BA   .......... .......... .......... .......... .......... .......... .......... .......... .A.....G.. ..........
DBP_2036_Brazil-BA   .......... .......... .......... .......... .......... .......... .......... .......... .A.....G.. ..........
DBP_2159_Brazil-BA   .......... .......... .......... .......... .......... .......... .......... .......... .......... ..........
DBP_2212_Brazil-BA   .......... .......... .......... .......... .......... .......... .......... .......... .A........ ..........
DBP_2272_Brazil-BA   .......... .......... .......... .......... .......... .......... .......... .......... .......... ..........
DBP_2327_Brazil-BA   .......... .......... .......... .......... .G........ .......... .......... .......... .......... ..........
DBP_2339_Brazil-BA   .......... .......... .......... .......... .......... .......... .......... .......... .......... ..........
DBP_2452_Brazil-BA   .......... .......... .......... .......... .......... .......... .......... .......... .......... ..........
DBP_2462_Brazil-BA   .......... .......... .......... .......... .......... .......... .......... .......... .......... ..........
DBP_2517_Brazil-BA   .......... .......... .......... .......... .......... .......... .......... .......... .A.....G.. ..........
DBP_2592_Brazil-BA   .......... .......... .......... .......... .......... .......... .......... .......... .......... ..........
DBP_2600_Brazil-BA   .......... .......... .......... .......... .......... .......... .......... .......... .......... ..........
DBP_2650_Brazil-BA   .......... .......... .......... .......... .......... .......... .......... .......... .......... ..........
DBP_2688_Brazil-BA   .......... .......... .......... .......... .......... .......... .......... .......... .A........ ..........
DBP_2695_Brazil-BA   .......... .......... .......... .......... .......... .......... .......... .......... .......... ..........
DBP_2692_Brazil-BA   .......... .......... .......... .......... .......... .......... .......... .......... .......... ..........
DBP_2693_Brazil-BA   .......... .......... .......... .......... .......... .......... .......... .......... .A........ ..........
```

```
DBP_2705_Brazil-BA     .......... .......... .......... .......... .......... .......... .......... .......... .......... ..........
DBP_2737_Brazil-BA     .......... .......... .......... .......... .......... .......... .......... .......... .......... ..........
DBP_2751_Brazil-BA     .......... .......... .......... .......... .......... .......... .......... .......... .......... ..........
DBP_2773_Brazil-BA     .......... .......... .......... .......... .......... .......... .......... .......... .A........ ..........
DBP_3124_Brazil-BA     .......... .......... .......... .......... .......... .......... .......... .......... .......... ..........
DBP_3164_Brazil-BA     .......... .......... .......... .......... .......... .......... .......... .......... .A........ ..........
DBP_3260_Brazil-BA     .......... .......... .......... .......... .......... .......... .......... .......... .A........ ..........
DBP_3280_Brazil-BA     .......... .......... .......... .......... .......... .......... .......... .......... .A........ ..........
DBP_3307_Brazil-BA     .......... .......... .......... .......... .......... .......... .......... .......... .A........ ..........
DBP_3311_Brazil-BA     .......... .......... .......... .......... .......... .......... .......... .......... .A........ ..........
DBP_3314_Brazil-BA     .......... .......... .......... .......... .......... .......... .......... .......... .A........ ..........
DBP_3340_Brazil-BA     .......... .......... .......... .......... .......... .......... .......... .......... .A........ ..........
DBP_3404_Brazil-BA     .......... .......... .......... .......... .......... .......... .......... .......... .A........ ..........
DBP_3507_Brazil-BA     .......... .......... .......... .......... .......... .......... .......... .......... .A.....G.. ..........
DBP_3516_Brazil-BA     .......... .......... .......... .......... .......... .......... .......... .......... .......... ..........
DBP_3530_Brazil-BA     .......... .......... .......... .......... .......... .......... .......... .......... .A........ ..........
DBP_3556_Brazil-BA     .......... .......... .......... .......... .......... .......... .......... .......... .......... ..........
DBP_3560_Brazil-BA     .......... .......... .......... .......... .......... .......... .......... .......... .A.....G.. ..........
DBP_3569_Brazil-BA     .......... .......... .......... .......... .......... .......... .......... .......... .......... ..........
DBP_3601_Brazil-BA     .......... .......... .......... .......... .......... .......... .......... .......... .......... ..........
DBP_3617_Brazil-BA     .......... .......... .......... .......... .......... .......... .......... .......... .A.....G.. ..........
DBP_3661_Brazil-BA     .......... .......... .......... .......... .......... .......... .......... .......... .A........ ..........
DBP_3707_Brazil-BA     .......... .......... .......... .......... .......... .......... .......... .......... .......... ..........
DBP_3814_Brazil-BA     .......... .......... .......... .......... .......... .......... .......... .......... .......... ..........
DBP_3842_Brazil-BA     .......... .......... .......... .......... .......... .......... .......... .......... .A........ ..........
DBP_3876_Brazil-BA     .......... .......... .......... .......... .......... .......... .......... .......... .A.....G.. ..........
DBP_3880_Brazil-BA     .......... .......... .......... .......... .......... .......... .......... .......... .......... ..........
DBP_3908_Brazil-BA     .......... .......... .......... .......... .......... .......... .......... .......... .A........ ..........
DBP_3913_Brazil-BA     .......... .......... .......... .......... .......... .......... .......... .......... .A........ ..........
DBP_3914_Brazil-BA     .......... .......... .......... .......... .......... .......... .......... .......... .......... ..........
DBP_3937_Brazil-BA     .......... .......... .......... .......... .......... .......... .......... .......... .A........ ..........
DBP_3974_Brazil-BA     .......... .......... .......... .......... .......... .......... .......... .......... .A........ ..........
DBP_3976_Brazil-BA     .......... .......... .......... .......... .......... .......... .......... .......... .A........ ..........
DBP_3998_Brazil-BA     .......... .......... .......... .......... .......... .......... .......... .......... .......... ..........
DBP_4030_Brazil-BA     .......... .......... .......... .......... .......... .......... .......... .......... .......... ..........
DBP_4071_Brazil-BA     .......... .......... .......... .......... .......... .......... .......... .......... .A........ ..........
DBP_4109_Brazil-BA     .......... .......... .......... .......... .......... .......... .......... .......... .A........ ..........
DBP_4135_Brazil-BA     .......... .......... ..A....... .......... .G........ .......... .......... .......... .......... ..........
DBP_1274_Brazil-AF     .......... .......... .......... .......... .......... .......... .......... .......... .A........ ..........
DBP_1347_Brazil-AF     .......... .......... .......... .......... .......... .......... .......... .......... .......... ..........
DBP_1436_Brazil-AF     .......... .......... ..A....... .......... .G........ .......... .......... .......... .......... ..........
DBP_1737_Brazil-AF     .......... .......... .......... .......... .......... .......... .......... .......... .A........ ..........
DBP_1652_Brazil-AF     .......... .......... .......... .......... .G........ .......... ...C...... .......... ......G... ..........
DBP_1745_Brazil-AF     .......... .......... .......... .......... .......... .......... .......... .......... .A........ ..........
DBP_1856_Brazil-AF     .......... .......... .......... .......... .G........ .......... ...C...... .......... .......... ..........
DBP_1906_Brazil-AF     .......... .......... .......... .......... .G........ .......... ...C...... .......... .......... ..........
DBP_1948_Brazil-AF     .......... .......... .......... .......... .G........ .......... ...C...... .......... .....C.... ..........
DBP_1960_Brazil-AF     .......... .......... .......... .......... .G........ .......... ...C...... .......... .......... ..........
DBP_1961_Brazil-AF     .......... .......... .......... .......... .G........ .......... ...C...... .......... ......G... ..........
DBP_2728_Brazil-AF     .......... .......... .......... .......... .G........ .......... ...C...... .......... .......... ..........
DBP_2734_Brazil-AF     .......... .......... .......... .......... .......... .......... .......... .......... .A........ ..........
```

```
DBP_2736_Brazil-AF   .......... .......... .......... .......... .G........ .......... ...C...... .......... .....C.... ..........
DBP_2747_Brazil-AF   .......... .......... .......... .......... .G........ .......... ...C...... .......... .......... ..........
DBP_2752_Brazil-AF   .......... .......... .......... .......... .G........ .......... ...C...... .......... .......... ..........
DBP_2762_Brazil-AF   .......... .......... .......... .......... .G........ .......... ...C...... .......... .......... ..........
DBP_2765_Brazil-AF   .......... .......... .......... .......... .G........ .......... ...C...... .......... .......... ..........
DBP_2769_Brazil-AF   .......... .......... .......... .......... .G........ .......... ...C...... .......... .....C.... ..........
DBP_2770_Brazil-AF   .......... .......... .......... .......... .G........ .......... ...C...... .......... .......... ..........
DBP_2775_Brazil-AF   .......... .......... .......... .......... .G........ .......... ...C...... .......... .....C.... ..........
DBP_2779_Brazil-AF   .......... .......... .......... .......... .G........ .......... ...C...... .......... .......... ..........
DBP_2782_Brazil-AF   .......... .......... .......... .......... .G........ .......... ...C...... .......... .....C.... ..........
DBP_2792_Brazil-AF   .......... .......... .......... .......... .G........ .......... ...C...... .......... ......G... ..........
DBP_2793_Brazil-AF   .......... .......... .......... .......... .G........ .......... ...C...... .......... .....C.... ..........
DBP_2816_Brazil-AF   .......... .......... .......... .......... .G........ .......... ...C...... .......... .....C.... ..........
DBP_2840_Brazil-AF   .......... .......... .......... .......... .G........ .......... ...C...... .......... .......... ..........
DBP_2859_Brazil-AF   .......... .......... .......... .......... .G........ .......... ...C...... .......... .......... ..........
DBP_2900_Brazil-AF   .......... .......... .......... .......... .G........ .......... ...C...... .......... .......... ..........
DBP_2916_Brazil-AF   .......... .......... .......... .......... .G........ .......... ...C...... .......... ......G... ..........
DBP_2923_Brazil-AF   .......... .......... .......... .......... .G........ .......... ...C...... .......... .....C.... ..........
DBP_2980_Brazil-AF   .......... .......... .......... .......... .......... .......... .......... .......... .A........ ..........
DBP_2982_Brazil-AF   .......... .......... .......... .......... .G........ .......... ...C...... .......... .....C.... ..........
DBP_2983_Brazil-AF   .......... .......... .......... .......... .......... .......... ...C...... .......... .A........ ..........
DBP_3071_Brazil-AF   .......... .......... .......... .......... .G........ .......... ...C...... .......... .....C.... ..........
DBP_3079_Brazil-AF   .......... .......... .......... .......... .G........ .......... ...C...... .......... .....C.... ..........
DBP_3084_Brazil-AF   .......... .......... .......... .......... .G........ .......... ...C...... .......... .......... ..........
DBP_3089_Brazil-AF   .......... .......... .......... .......... .G........ .......... ...C...... .......... .......... ..........
DBP_3157_Brazil-AF   .......... .......... .......... .......... .......... .......... .......... .......... .A........ ..........
DBP_3276_Brazil-AF   .......... .......... .......... .......... .......... .......... .......... .......... .A........ ..........
DBP_3277_Brazil-AF   .......... .......... .......... .......... .G........ .......... ...C...... .......... .....C.... ..........
DBP_3278_Brazil-AF   .......... .......... .......... .......... .G........ .......... ...C...... .......... .....C.... ..........
DBP_3284_Brazil-AF   .......... .......... .......... .......... .G........ .......... ...C...... .......... .....C.... ..........
DBP_3296_Brazil-AF   .......... .......... .......... .......... .G........ .......... ...C...... .......... .......... ..........
DBP_3300_Brazil-AF   .......... .......... .......... .......... .G........ .......... ...C...... .......... .......... ..........
DBP_3310_Brazil-AF   .......... .......... .......... .......... .G........ .......... ...C...... .......... .......... ..........
DBP_3315_Brazil-AF   .......... .......... .......... .......... .G........ .......... ...C...... .......... .......... ..........
DBP_3325_Brazil-AF   .......... .......... .......... .......... .......... .......... .......... .......... .A........ ..........
DBP_3334_Brazil-AF   .......... .......... .......... .......... .G........ .......... ...C...... .......... ......G... ..........
DBP_3371_Brazil-AF   .......... .......... .......... .......... .G........ .......... ...C...... .......... .....C.... ..........
DBP_3376_Brazil-AF   .......... .......... .......... .......... .G........ .......... ...C...... .......... .......... ..........
DBP_3379_Brazil-AF   .......... .......... .......... .......... .G........ .......... ...C...... .......... .......... ..........
DBP_3406_Brazil-AF   .......... .......... .......... .......... .G........ .......... ...C...... .......... .......... ..........
DBP_3612_Brazil-AF   .......... .......... .......... .......... .G........ .......... ...C...... .......... .......... ..........
DBP_3613_Brazil-AF   .......... .......... .......... .......... .G........ .......... ...C...... .......... .......... ..........
DBP_3614_Brazil-AF   .......... .......... .......... .......... .G........ .......... ...C...... .......... ......G... ..........
DBP_3616_Brazil-AF   .......... .......... .......... .......... .G........ .......... ...C...... .......... .....C.... ..........
DBP_3630_Brazil-AF   .......... .......... .......... .......... .G........ .......... ...C...... .......... .......... ..........
DBP_3633_Brazil-AF   .......... .......... .......... .......... .G........ .......... ...C...... .......... .....C.... ..........
DBP_3643_Brazil-AF   .......... .......... .......... .......... .G........ .......... ...C...... .......... .....C.... ..........
DBP_3659_Brazil-AF   .......... .......... .......... .......... .G........ .......... ...C...... .......... .......... ..........
DBP_3698_Brazil-AF   .......... .......... .......... .......... .G........ .......... ...C...... .......... .....C.... ..........
DBP_3705_Brazil-AF   .......... .......... .......... .......... .......... .......... .......... .......... .......... ..........
DBP_3722_Brazil-AF   .......... .......... .......... .......... .G........ .......... ...C...... .......... .......... ..........
```

```
DBP_3735_Brazil-AF        .......... .......... .......... .......... .G........ .......... ...C...... .......... .......... ..........
DBP_3736_Brazil-AF        .......... .......... .......... .......... .......... .......... .......... .......... .A........ ..........
DBP_3739_Brazil-AF        .......... .......... .......... .......... .G........ .......... ...C...... .......... .......... ..........
DBP_3746_Brazil-AF        .......... .......... .......... .......... .......... .......... .......... .......... .A........ ..........
DBP_3750_Brazil-AF        .......... .......... .......... .......... .......... .......... .......... .......... .A........ ..........
DBP_3916_Brazil-AF        .......... .......... .......... .......... .......... .......... .......... .......... .......... ..........
DBP_3931_Brazil-AF        .......... .......... .......... .......... .G........ .......... ...C...... .......... .....C.... ..........
DBP_3962_Brazil-AF        .......... .......... .......... .......... .......... .......... .......... .......... .......... ..........
DBP_4083_Brazil-AF        .......... .......... .......... .......... .G........ .......... ...C...... .......... .......... ..........
DBP_4096_Brazil-AF        .......... .......... .......... .......... .......... .......... .......... .......... .A........ ..........
DBP_4097_Brazil-AF        .......... .......... .......... .......... .G........ .......... ...C...... .......... .......... ..........
DBP_4112_Brazil-AF        .......... .......... .......... .......... .......... .......... .......... .......... .A........ ..........
DBP_4118_Brazil-AF        .......... .......... .......... .......... .G........ .......... ...C...... .......... .......... ..........
DBP_4131_Brazil-AF        .......... .......... .......... .......... .......... .......... .......... .......... .A........ ..........
DBP_4161_Brazil-AF        .......... .......... .......... .......... .......... .......... ...C...... .......... .A........ ..........

                                 410        420        430        440        450        460        470        480        490        500
                          ....|....| ....|....| ....|....| ....|....| ....|....| ....|....| ....|....| ....|....| ....|....| ....|....|
PVX_110810_|_Salvador 1   TTAAATGTTG CGGTAAATAT AGAACCGCAG ATATATAGAT GGATTCGAGA ATGGGGAAGG GATTACGTGT CAGAATTGCC CACAGAAGTG CAAAAACTGA
DBP_07_Brazil-BA          A......... .......... .......... .......... .......... .......... .......... .......... .......... ..........
DBP_09_Brazil-BA          A......... .......... .......... .........C .......... .......... .......... .......... .......... ..........
DBP_10_Brazil-BA          A......... .......... .......... .........C .......... .......... .......... .......... .......... ..........
DBP_11_Brazil-BA          .......... .......... .......... .......... .......... .......... .......... .......... .......... ..........
DBP_12_Brazil-BA          A......... .......... .......... .......... .......... .......... .......... .......... .......... ..........
DBP_13_Brazil-BA          .......... .......... .......... .......... .......... .......... .......... .......... .......... ..........
DBP_14_Brazil-BA          .......... .......... .......... .......... .......... .......... .......... .......... .......... ..........
DBP_15_Brazil-BA          A......... .......... .......... .........C .......... .......... .......... .......... .......... ..........
DBP_16_Brazil-BA          A......... .......... .......... .......... .......... .......... .......... .......... .......... ..........
DBP_17_Brazil-BA          A......... .......... .......... .........C .......... .......... .......... .......... .......... ..........
DBP_18_Brazil-BA          .......... .......... .......... .......... .......... .......... .......... .......... .......... ..........
DBP_20_Brazil-BA          .......... .......... .......... .......... .......... .......... .......... .......... .......... ..........
DBP_21_Brazil-BA          .......... .......... .......... .......... .......... .......... .......... .......... .......... ..........
DBP_22_Brazil-BA          .......... .......... .......... .......... .......... .......... .......... .......... .......... ..........
DBP_24_Brazil-BA          A......... .......... .......... .........C .......... .......... .......... .......... .......... .C........
DBP_26_Brazil-BA          A......... .......... .......... .........C .......... .......... .......... .......... .......... ..........
DBP_27_Brazil-BA          A......... .......... .......... .......... .......... .......... .......... .......... .......... ..........
DBP_28_Brazil-BA          .......... .......... .......... .......... .......... .......... .......... .......... .......... ..........
DBP_29_Brazil-BA          .......... .......... .......... .......... .......... .......... .......... .......... .......... ..........
DBP_31_Brazil-BA          A......... .......... .......... .........C .......... .......... .......... .......... .......... ..........
DBP_32_Brazil-BA          A......... .......... .......... .........C .......... .......... .......... .......... .......... ..........
DBP_33_Brazil-BA          .......... .......... .......... .......... .......... .......... .......... .......... .......... ..........
DBP_35_Brazil-BA          .......... .......... .......... .......... .......... .......... .......... .......... .......... ..........
DBP_36_Brazil-BA          .......... .......... .......... .........C .......... .......... .......... .......... .......... ..........
DBP_37_Brazil-BA          .......... .......... .......... .......... .......... .......... .......... .......... .......... ..........
DBP_38_Brazil-BA          .......... .......... .......... .........C .......... .......... .......... .......... .......... ..........
DBP_39_Brazil-BA          .......... .......... .......... .......... .......... .......... .......... .......... .......... ..........
DBP_40_Brazil-BA          A......... .......... .......... .........C .......... .......... .......... .......... .......... ..........
DBP_41_Brazil-BA          .......... .......... .......... .......... .......... .......... .......... .......... .......... ..........
DBP_42_Brazil-BA          .......... .......... .......... .......... .......... .......... .......... .......... .......... ..........
DBP_43_Brazil-BA          .......... .......... .......... .......... .......... .......... .......... .......... .......... ..........
DBP_44_Brazil-BA          .......... .......... .......... .......... .......... .......... .......... .......... .......... ..........
```

```
DBP_45_Brazil-BA      .......... .......... .......... .......... .......... .......... .......... .......... .......... ..........
DBP_46_Brazil-BA      .......... .......... .......... .......... .......... .......... .......... .......... .......... ..........
DBP_47_Brazil-BA      A......... .......... .......... .........C .......... .......... .......... .......... .......... ..........
DBP_48_Brazil-BA      .......... .......... .......... .......... .......... .......... .......... .......... .......... ..........
DBP_49_Brazil-BA      A......... .......... .......... .........C .......... .......... .......... .......... .......... ..........
DBP_51_Brazil-BA      A......... .......... .......... .........C .......... .......... .......... .......... .......... ..........
DBP_53_Brazil-BA      A......... .......... .......... .........C .......... .......... .......... .......... .......... ..........
DBP_54_Brazil-BA      .......... .......... .......... .......... .......... .......... .......... .......... .......... ..........
DBP_55_Brazil-BA      A......... .......... .......... .......... .......... .......... .......... .......... .......... ..........
DBP_56_Brazil-BA      .......... .......... .......... .......... .......... .......... .......... .......... .......... ..........
DBP_57_Brazil-BA      .......... .......... .......... .........C .......... .......... .......... .......... .......... ..........
DBP_58_Brazil-BA      .......... .......... .......... .......... .......... .......... .......... .......... .......... ..........
DBP_59_Brazil-BA      A......... .......... .......... .........C .......... .......... .......... .......... .......... .C........
DBP_1161_Brazil-BA    A......... .......... .......... .......... .......... .......... .......... .......... .......... ..........
DBP_1182_Brazil-BA    .......... .......... .......... .......... .......... .......... .......... .......... .......... ..........
DBP_1190_Brazil-BA    A......... .......... .......... .........C .......... .......... .......... .......... .......... ..........
DBP_1200_Brazil-BA    A......... .......... .......... .........C .......... .......... .......... .......... .......... ..........
DBP_1209_Brazil-BA    A......... .......... .......... .......... .......... .......... .......... .......... .......... ..........
DBP_1272_Brazil-BA    .......... .......... .......... .......... .......... .......... .......... .......... .......... ..........
DBP_1295_Brazil-BA    A......... .......... .......... .........C .......... .......... .......... .......... .......... ..........
DBP_1320_Brazil-BA    .......... .......... .......... .......... .......... .......... .......... .......... .......... ..........
DBP_1327_Brazil-BA    .......... .......... .......... .......... .......... .......... .......... .......... .......... ..........
DBP_1335_Brazil-BA    .......... .......... .......... .......... .......... .......... .......... .......... .......... ..........
DBP_1340_Brazil-BA    A......... .......... .......... .........C .......... .......... .......... .......... .......... ..........
DBP_1346_Brazil-BA    A......... .......... .......... .........C .......... .......... .......... .......... .......... ..........
DBP_1382_Brazil-BA    A......... .......... .......... .........C .......... .......... .......... .......... .......... ..........
DBP_1428_Brazil-BA    A......... .......... .......... .........C .......... .......... .......... .......... .......... ..........
DBP_1447_Brazil-BA    A......... .......... .......... .........C .......... .......... .......... .......... .......... .C........
DBP_1469_Brazil-BA    .......... .......... .......... .......... .......... .......... .......... .......... .......... .C........
DBP_1473_Brazil-BA    .......... .......... .......... .......... .......... .......... .......... .......... .......... ..........
DBP_1475_Brazil-BA    A......... .......... .......... .........C .......... .......... .......... .......... .......... .C........
DBP_1493_Brazil-BA    .......... .......... .......... .......... .......... .......... .......... .......... .......... ..........
DBP_1532_Brazil-BA    A......... .......... .......... .........C .......... .......... .......... .......... .......... ..........
DBP_1540_Brazil-BA    .......... .......... .......... .......... .......... .......... .......... .......... .......... ..........
DBP_1621_Brazil-BA    A......... .......... .......... .........C .......... .......... .......... .......... .......... .C........
DBP_1627_Brazil-BA    A......... .......... .......... .........C .......... .......... .......... .......... .......... ..........
DBP_1641_Brazil-BA    .......... .......... .......... .......... .......... .......... .......... .......... .......... ..........
DBP_1664_Brazil-BA    .......... .......... .......... .......... .......... .......... .......... .......... .......... ..........
DBP_1741_Brazil-BA    A......... .......... .......... .........C .......... .......... .......... .......... .......... ..........
DBP_1747_Brazil-BA    .......... .......... .......... .......... .......... .......... .......... .......... .......... ..........
DBP_1748_Brazil-BA    .......... .......... .......... .......... .......... .......... .......... .......... .......... ..........
DBP_1777_Brazil-BA    .......... .......... .......... .......... .......... .......... .......... .......... .......... ..........
DBP_1789_Brazil-BA    .......... .......... .......... .......... .......... .......... .......... .......... .......... ..........
DBP_1812_Brazil-BA    A......... .......... .......... .........C .......... .......... .......... .......... .......... ..........
DBP_1824_Brazil-BA    A......... .......... .......... .........C .......... .......... .......... .......... .......... ..........
DBP_1830_Brazil-BA    A......... .......... .......... .........C .......... .......... .......... .......... .......... .C........
DBP_1837_Brazil-BA    A......... .......... .......... .........C .......... .......... .......... .......... .......... ..........
DBP_1853_Brazil-BA    A......... .......... .......... .........C .......... .......... .......... .......... .......... ..........
DBP_1867_Brazil-BA    A......... .......... .......... .........C .......... .......... .......... .......... .......... ..........
DBP_1914_Brazil-BA    A......... .......... .......... .......... .......... .......... .......... .......... .......... ..........
DBP_1874_Brazil-BA    A......... .......... .......... .........C .......... .......... .......... .......... .......... ..........
```

```
DBP_2036_Brazil-BA    A......... .......... .......... .......... .......... .......... .......... .......... .......... ..........
DBP_2159_Brazil-BA    .......... .......... .......... .......... .......... .......... .......... .......... .......... ..........
DBP_2212_Brazil-BA    A......... .......... .......... .........C .......... .......... .......... .......... .......... ..........
DBP_2272_Brazil-BA    .......... .......... .......... .......... .......... .......... .......... .......... .......... ..........
DBP_2327_Brazil-BA    A......... .......... .......... .......... .......... .......... .......... .......... .......... ..........
DBP_2339_Brazil-BA    .......... .......... .......... .......... .......... .......... .......... .......... .......... ..........
DBP_2452_Brazil-BA    A......... .......... .......... .........C .......... .......... .......... .......... .......... ..........
DBP_2462_Brazil-BA    .......... .......... .......... .......... .......... .......... .......... .......... .......... ..........
DBP_2517_Brazil-BA    A......... .......... .......... .........C .......... .......... .......... .......... .......... ..........
DBP_2592_Brazil-BA    .......... .......... .......... .......... .......... .......... .......... .......... .......... ..........
DBP_2600_Brazil-BA    .......... .......... .......... .......... .......... .......... .......... .......... .......... ..........
DBP_2650_Brazil-BA    A......... .......... .......... .........C .......... .......... .......... .......... .......... ..........
DBP_2688_Brazil-BA    A......... .......... .......... .........C .......... .......... .......... .......... .......... ..........
DBP_2695_Brazil-BA    .......... .......... .......... .......... .......... .......... .......... .......... .......... ..........
DBP_2692_Brazil-BA    .......... .......... .......... .........C .......... .......... .......... .......... .......... .C........
DBP_2693_Brazil-BA    A......... .......... .......... .........C .......... .......... .......... .......... .......... ..........
DBP_2705_Brazil-BA    .......... .......... .......... .......... .......... .......... .......... .......... .......... ..........
DBP_2737_Brazil-BA    .......... .......... .......... .......... .......... .......... .......... .......... .......... ..........
DBP_2751_Brazil-BA    .......... .......... .......... .......... .......... .......... .......... .......... .......... ..........
DBP_2773_Brazil-BA    A......... .......... .......... .........C .......... .......... .......... .......... .......... ..........
DBP_3124_Brazil-BA    .......... .......... .......... .......... .......... .......... .......... .......... .......... .C........
DBP_3164_Brazil-BA    A......... .......... .......... .........C .......... .......... .......... .......... .......... .C........
DBP_3260_Brazil-BA    A......... .......... .......... .........C .......... .......... .......... .......... .......... ..........
DBP_3280_Brazil-BA    A......... .......... .......... .........C .......... .......... .......... .......... .......... ..........
DBP_3307_Brazil-BA    A......... .......... .......... .........C .......... .......... .......... .......... .......... ..........
DBP_3311_Brazil-BA    A......... .......... .......... .........C .......... .......... .......... .......... .......... ..........
DBP_3314_Brazil-BA    A......... .......... .......... .........C .......... .......... .......... .......... .......... ..........
DBP_3340_Brazil-BA    A......... .......... .......... .........C .......... .......... .......... .......... .......... ..........
DBP_3404_Brazil-BA    A......... .......... .......... .........C .......... .......... .......... .......... .......... ..........
DBP_3507_Brazil-BA    A......... .......... .......... .........C .......... .......... .......... .......... .......... ..........
DBP_3516_Brazil-BA    .......... .......... .......... .......... .......... .......... .......... .......... .......... ..........
DBP_3530_Brazil-BA    A......... .......... .......... .........C .......... .......... .......... .......... .......... ..........
DBP_3556_Brazil-BA    .......... .......... .......... .......... .......... .......... .......... .......... .......... ..........
DBP_3560_Brazil-BA    A......... .......... .......... .........C .......... .......... .......... .......... .......... ..........
DBP_3569_Brazil-BA    .......... .......... .......... .......... .......... .......... .......... .......... .......... ..........
DBP_3601_Brazil-BA    .......... .......... .......... .......... .......... .......... .......... .......... .......... ..........
DBP_3617_Brazil-BA    A......... .......... .......... .........C .......... .......... .......... .......... .......... ..........
DBP_3661_Brazil-BA    A......... .......... .......... .........C .......... .......... .......... .......... .......... ..........
DBP_3707_Brazil-BA    .......... .......... .......... .......... .......... .......... .......... .......... .......... ..........
DBP_3814_Brazil-BA    A......... .......... .......... .........C .......... .......... .......... .......... .......... ..........
DBP_3842_Brazil-BA    A......... .......... .......... .........C .......... .......... .......... .......... .......... ..........
DBP_3876_Brazil-BA    .......... .......... .......... .........C .......... .......... .......... .......... .......... ..........
DBP_3880_Brazil-BA    .......... .......... .......... .........C .......... .......... .......... .......... .......... ..........
DBP_3908_Brazil-BA    A......... .......... .......... .........C .......... .......... .......... .......... .......... .C........
DBP_3913_Brazil-BA    A......... .......... .......... .........C .......... .......... .......... .......... .......... ..........
DBP_3914_Brazil-BA    .......... .......... .......... .......... .......... .......... .......... .......... .......... ..........
DBP_3937_Brazil-BA    A......... .......... .......... .........C .......... .......... .......... .......... .......... ..........
DBP_3974_Brazil-BA    A......... .......... .......... .........C .......... .......... .......... .......... .......... ..........
DBP_3976_Brazil-BA    A......... .......... .......... .........C .......... .......... .......... .......... .......... ..........
DBP_3998_Brazil-BA    A......... .......... .......... .........C .......... .......... .......... .......... .......... ..........
DBP_4030_Brazil-BA    .......... .......... .......... .......... .......... .......... .......... .......... .......... ..........
```

```
DBP_4071_Brazil-BA    A......... .......... .......... .........C .......... .......... .......... .......... .......... ..........
DBP_4109_Brazil-BA    A......... .......... .......... .........C .......... .......... .......... .......... .......... .C........
DBP_4135_Brazil-BA    A......... .......... .......... .......... .......... .......... .......... .......... .......... ..........
DBP_1274_Brazil-AF    A......... .......... .......... .........C .......... .......... .......... .......... .......... ..........
DBP_1347_Brazil-AF    .......... .......... .......... .......... .......... .......... .......... .......... .......... ..........
DBP_1436_Brazil-AF    A......... .......... .......... .......... .......... .......... .......... .......... .......... ..........
DBP_1737_Brazil-AF    A......... .......... .......... .........C .......... .......... .......... .......... .......... ..........
DBP_1652_Brazil-AF    A......... .......... .......... .......... .......... .......... .......... .......... .......... ..........
DBP_1745_Brazil-AF    A......... .......... .......... .........C .......... .......... .......... .......... .......... ..........
DBP_1856_Brazil-AF    A......... .......... .......... .......... .......... .......... .......... .......... .......... ..........
DBP_1906_Brazil-AF    A......... .......... .......... .......... .......... .......... .......... .......... .......... ..........
DBP_1948_Brazil-AF    A......... .......... .......... .......... .......... .......... .......... .......... .......... ..........
DBP_1960_Brazil-AF    A......... .......... .......... .......... .......... .......... .......... .......... .......... ..........
DBP_1961_Brazil-AF    A......... .......... .......... .......... .......... .......... .......... .......... .......... ..........
DBP_2728_Brazil-AF    A......... .......... .......... .......... .......... .......... .......... .......... .......... ..........
DBP_2734_Brazil-AF    A......... .......... .......... .........C .......... .......... .......... .......... .......... ..........
DBP_2736_Brazil-AF    A......... .......... .......... .......... .......... .......... .......... .......... .......... ..........
DBP_2747_Brazil-AF    A......... .......... .......... .......... .......... .......... .......... .......... .......... ..........
DBP_2752_Brazil-AF    A......... .......... .......... .......... .......... .......... .......... .......... .......... ..........
DBP_2762_Brazil-AF    A......... .......... .......... .......... .......... .......... .......... .......... .......... ..........
DBP_2765_Brazil-AF    A......... .......... .......... .......... .......... .......... .......... .......... .......... ..........
DBP_2769_Brazil-AF    A......... .......... .......... .......... .......... .......... .......... .......... .......... ..........
DBP_2770_Brazil-AF    A......... .......... .......... .......... .......... .......... .......... .......... .......... ..........
DBP_2775_Brazil-AF    A......... .......... .......... .......... .......... .......... .......... .......... .......... ..........
DBP_2779_Brazil-AF    A......... .......... .......... .......... .......... .......... .......... .......... .......... ..........
DBP_2782_Brazil-AF    A......... .......... .......... .......... .......... .......... .......... .......... .......... ..........
DBP_2792_Brazil-AF    A......... .......... .......... .......... .......... .......... .......... .......... .......... ..........
DBP_2793_Brazil-AF    A......... .......... .......... .......... .......... .......... .......... .......... .......... ..........
DBP_2816_Brazil-AF    A......... .......... .......... .......... .......... .......... .......... .......... .......... ..........
DBP_2840_Brazil-AF    A......... .......... .......... .......... .......... .......... .......... .......... .......... ..........
DBP_2859_Brazil-AF    A......... .......... .......... .......... .......... .......... .......... .......... .......... ..........
DBP_2900_Brazil-AF    A......... .......... .......... .......... .......... .......... .......... .......... .......... ..........
DBP_2916_Brazil-AF    A......... .......... .......... .......... .......... .......... .......... .......... .......... ..........
DBP_2923_Brazil-AF    A......... .......... .......... .......... .......... .......... .......... .......... .......... ..........
DBP_2980_Brazil-AF    A......... .......... .......... .........C .......... .......... .......... .......... .......... ..........
DBP_2982_Brazil-AF    A......... .......... .......... .......... .......... .......... .......... .......... .......... ..........
DBP_2983_Brazil-AF    A......... .......... .......... .........C .......... .......... .......... .......... .......... ..........
DBP_3071_Brazil-AF    A......... .......... .......... .......... .......... .......... .......... .......... .......... ..........
DBP_3079_Brazil-AF    A......... .......... .......... .......... .......... .......... .......... .......... .......... ..........
DBP_3084_Brazil-AF    A......... .......... .......... .......... .......... .......... .......... .......... .......... ..........
DBP_3089_Brazil-AF    A......... .......... .......... .......... .......... .......... .......... .......... .......... ..........
DBP_3157_Brazil-AF    A......... .......... .......... .........C .......... .......... .......... .......... .......... ..........
DBP_3276_Brazil-AF    A......... .......... .......... .........C .......... .......... .......... .......... .......... ..........
DBP_3277_Brazil-AF    A......... .......... .......... .......... .......... .......... .......... .......... .......... ..........
DBP_3278_Brazil-AF    A......... .......... .......... .......... .......... .......... .......... .......... .......... ..........
DBP_3284_Brazil-AF    A......... .......... .......... .......... .......... .......... .......... .......... .......... ..........
DBP_3296_Brazil-AF    A......... .......... .......... .......... .......... .......... .......... .......... .......... ..........
DBP_3300_Brazil-AF    A......... .......... .......... .......... .......... .......... .......... .......... .......... ..........
DBP_3310_Brazil-AF    A......... .......... .......... .......... .......... .......... .......... .......... .......... ..........
DBP_3315_Brazil-AF    A......... .......... .......... .......... .......... .......... .......... .......... .......... ..........
DBP_3325_Brazil-AF    A......... .......... .......... .........C .......... .......... .......... .......... .......... ..........
```

```
DBP_3334_Brazil-AF      A......... .......... .......... .......... .......... .......... .......... .......... .......... ..........
DBP_3371_Brazil-AF      A......... .......... .......... .......... .......... .......... .......... .......... .......... ..........
DBP_3376_Brazil-AF      A......... .......... .......... .......... .......... .......... .......... .......... .......... ..........
DBP_3379_Brazil-AF      A......... .......... .......... .......... .......... .......... .......... .......... .......... ..........
DBP_3406_Brazil-AF      A......... .......... .......... .......... .......... .......... .......... .......... .......... ..........
DBP_3612_Brazil-AF      A......... .......... .......... .......... .......... .......... .......... .......... .......... ..........
DBP_3613_Brazil-AF      A......... .......... .......... .......... .......... .......... .......... .......... .......... .C........
DBP_3614_Brazil-AF      A......... .......... .......... .......... .......... .......... .......... .......... .......... .C........
DBP_3616_Brazil-AF      A......... .......... .......... .......... .......... .......... .......... .......... .......... .C........
DBP_3630_Brazil-AF      A......... .......... .......... .......... .......... .......... .......... .......... .......... .C........
DBP_3633_Brazil-AF      A......... .......... .......... .......... .......... .......... .......... .......... .......... .C........
DBP_3643_Brazil-AF      A......... .......... .......... .......... .......... .......... .......... .......... .......... ..........
DBP_3659_Brazil-AF      A......... .......... .......... .......... .......... .......... .......... .......... .......... .C........
DBP_3698_Brazil-AF      A......... .......... .......... .......... .......... .......... .......... .......... .......... ..........
DBP_3705_Brazil-AF      .......... .......... .......... .......... .......... .......... .......... .......... .......... ..........
DBP_3722_Brazil-AF      A......... .......... .......... .......... .......... .......... .......... .......... .......... ..........
DBP_3735_Brazil-AF      A......... .......... .......... .......... .......... .......... .......... .......... .......... ..........
DBP_3736_Brazil-AF      A......... .......... .......... .........C .......... .......... .......... .......... .......... ..........
DBP_3739_Brazil-AF      A......... .......... .......... .......... .......... .......... .......... .......... .......... ..........
DBP_3746_Brazil-AF      A......... .......... .......... .........C .......... .......... .......... .......... .......... ..........
DBP_3750_Brazil-AF      A......... .......... .......... .........C .......... .......... .......... .......... .......... ..........
DBP_3916_Brazil-AF      A......... .......... .......... .........C .......... .......... .......... .......... .......... ..........
DBP_3931_Brazil-AF      A......... .......... .......... .......... .......... .......... .......... .......... .......... ..........
DBP_3962_Brazil-AF      A......... .......... .......... .......... .......... .......... .......... .......... .......... ..........
DBP_4083_Brazil-AF      A......... .......... .......... .......... .......... .......... .......... .......... .......... ..........
DBP_4096_Brazil-AF      A......... .......... .......... .........C .......... .......... .......... .......... .......... ..........
DBP_4097_Brazil-AF      A......... .......... .......... .......... .......... .......... .......... .......... .......... ..........
DBP_4112_Brazil-AF      A......... .......... .......... .........C .......... .......... .......... .......... .......... ..........
DBP_4118_Brazil-AF      A......... .......... .......... .......... .......... .......... .......... .......... .......... ..........
DBP_4131_Brazil-AF      A......... .......... .......... .........C .......... .......... .......... .......... .......... .C........
DBP_4161_Brazil-AF      A......... .......... .......... .........C .......... .......... .......... .......... .......... .C........
```

```
                               510        520        530        540        550        560        570        580        590        600
                         ....|....| ....|....| ....|....| ....|....| ....|....| ....|....| ....|....| ....|....| ....|....| ....|....|
PVX_110810_|_Salvador 1  AAGAAAAATG TGATGGAAAA ATCAATTATA CTGATAAAAA AGTATGTAAG GTACCACCAT GTCAAAATGC GTGTAAATCA TATGATCAAT GGATAACCAG
DBP_07_Brazil-BA         .......... .......... .......... .......... .......... .......... .......... .......... .......... ..........
DBP_09_Brazil-BA         .......... .......... .......... .......... .......... .......... .......... .......... .......... ..........
DBP_10_Brazil-BA         .......... .......... .......... .......... .......... .......... .......... .......... .......... ..........
DBP_11_Brazil-BA         .......... .......... .......... .......... .......... .......... .......... .......... .......... ..........
DBP_12_Brazil-BA         .......... .......... .......... .......... .......... .......... .......... .......... .......... ..........
DBP_13_Brazil-BA         .......... .......... .......... .......... .......... .......... .......... .......... .......... ..........
DBP_14_Brazil-BA         .......... .......... .......... .......... .......... .......... .......... .......... .......... ..........
DBP_15_Brazil-BA         .......... .......... .......... .......... .......... .......... .......... .......... .......... ..........
DBP_16_Brazil-BA         .......... .......... .......... .......... .......... .......... .......... .......... .......... ..........
DBP_17_Brazil-BA         .......... .......... .......... .......... .......... .......... .......... .......... .......... ..........
DBP_18_Brazil-BA         .......... .......... .......... .......... .......... .......... .......... .......... .......... ..........
DBP_20_Brazil-BA         .......... .......... .......... .......... .......... .......... .......... .......... .......... ..........
DBP_21_Brazil-BA         .......... .......... .......... .......... .......... .......... .......... .......... .......... ..........
DBP_22_Brazil-BA         .......... .......... .......... .......... .......... .......... .......... .......... .......... ..........
DBP_24_Brazil-BA         .......... .......... .......... .......... .......... .......... .......... .......... .......... ..........
DBP_26_Brazil-BA         .......... .......... .......... .......... .......... .......... .......... .......... .......... ..........
DBP_27_Brazil-BA         .......... .......... .......... .......... .......... .......... .......... .......... .......... ..........
DBP_28_Brazil-BA         .......... .......... .......... .......... .......... .......... .......... .......... .......... ..........
DBP_29_Brazil-BA         .......... .......... .......... .......... .......... .......... .......... .......... .......... ..........
DBP_31_Brazil-BA         .......... .......... .......... .......... .......... .......... .......... .......... .......... ..........
DBP_32_Brazil-BA         .......... .......... .......... .......... .......... .......... .......... .......... .......... ..........
DBP_33_Brazil-BA         .......... .......... .......... .......... .......... .......... .......... .......... .......... ..........
DBP_35_Brazil-BA         .......... .......... .......... .......... .......... .......... .......... .......... .......... ..........
DBP_36_Brazil-BA         .......... .......... .......... .......... .......... .......... .......... .......... .......... ..........
DBP_37_Brazil-BA         .......... .......... .......... .......... .......... .......... .......... .......... .......... ..........
DBP_38_Brazil-BA         .......... .......... .......... .......... .......... .......... .......... .......... .......... ..........
DBP_39_Brazil-BA         .......... .......... .......... .......... .......... .......... .......... .......... .......... ..........
DBP_40_Brazil-BA         .......... .......... .......... .......... .......... .......... .......... .......... .......... ..........
DBP_41_Brazil-BA         .......... .......... .......... .......... .......... .......... .......... .......... .......... ..........
DBP_42_Brazil-BA         .......... .......... .......... .......... .......... .......... .......... .......... .......... ..........
DBP_43_Brazil-BA         .......... .......... .......... .......... .......... .......... .......... .......... .......... ..........
DBP_44_Brazil-BA         .......... .......... .......... .......... .......... .......... .......... .......... .......... ..........
DBP_45_Brazil-BA         .......... .......... .......... .......... .......... .......... .......... .......... .......... ..........
DBP_46_Brazil-BA         .......... .......... .......... .......... .......... .......... .......... .......... .......... ..........
DBP_47_Brazil-BA         .......... .......... .......... .......... .......... .......... .......... .......... .......... ..........
DBP_48_Brazil-BA         .......... .......... .......... .......... .......... .......... .......... .......... .......... ..........
DBP_49_Brazil-BA         .......... .......... .......... .......... .......... .......... .......... .......... .......... ..........
DBP_51_Brazil-BA         .......... .......... .......... .......... .......... .......... .......... .......... .......... ..........
DBP_53_Brazil-BA         .......... .......... .......... .......... .......... .......... .......... .......... .......... ..........
DBP_54_Brazil-BA         .......... .......... .......... .......... .......... .......... .......... .......... .......... ..........
DBP_55_Brazil-BA         .......... .......... .......... .......... .......... .......... .......... .......... .......... ..........
DBP_56_Brazil-BA         .......... .......... .......... .......... .......... .......... .......... .......... .......... ..........
DBP_57_Brazil-BA         .......... .......... .......... .......... .......... .......... .......... .......... .......... ..........
DBP_58_Brazil-BA         .......... .......... .......... .......... .......... .......... .......... .......... .......... ..........
DBP_59_Brazil-BA         .......... .......... .......... .......... .......... .......... .......... .......... .......... ..........
DBP_1161_Brazil-BA       .......... .......... .......... .......... .......... .......... .......... .......... .......... ..........
DBP_1182_Brazil-BA       .......... .......... .......... .......... .......... .......... .......... .......... .......... ..........
DBP_1190_Brazil-BA       .......... .......... .......... .......... .......... .......... .......... .......... .......... ..........
```

```
DBP_1200_Brazil-BA        .......... .......... .......... .......... .......... .......... .......... .......... .......... ..........
DBP_1209_Brazil-BA        .......... .......... .......... .......... .......... .......... .......... .......... .......... ..........
DBP_1272_Brazil-BA        .......... .......... .......... .......... .......... .......... .......... .......... .......... ..........
DBP_1295_Brazil-BA        .......... .......... .......... .......... .......... .......... .......... .......... .......... ..........
DBP_1320_Brazil-BA        .......... .......... .......... .......... .......... .......... .......... .......... .......... ..........
DBP_1327_Brazil-BA        .......... .......... .......... .......... .......... .......... .......... .......... ......G... ..........
DBP_1335_Brazil-BA        .......... .......... .......... .......... .......... .......... .......... .......... .......... ..........
DBP_1340_Brazil-BA        .......... .......... .......... .......... .......... .......... .......... .......... .......... ..........
DBP_1346_Brazil-BA        .......... .......... .......... .......... .......... .......... .......... .......... .......... ..........
DBP_1382_Brazil-BA        .......... .......... .......... .......... .......... .......... .......... .......... .......... ..........
DBP_1428_Brazil-BA        .......... .......... .......... .......... .......... .......... .......... .......... .......... ..........
DBP_1447_Brazil-BA        .......... .......... .......... .......... .......... .......... .......... .......... .......... ..........
DBP_1469_Brazil-BA        .......... .......... .......... .......... .......... .......... .......... .......... .......... ..........
DBP_1473_Brazil-BA        .......... .......... .......... .......... .......... .......... .......... .......... .......... ..........
DBP_1475_Brazil-BA        .......... .......... .......... .......... .......... .......... .......... .......... .......... ..........
DBP_1493_Brazil-BA        .......... .......... .......... .......... .......... .......... .......... .......... .......... ..........
DBP_1532_Brazil-BA        .......... .......... .......... .......... .......... .......... .......... .......... .......... ..........
DBP_1540_Brazil-BA        .......... .......... .......... .......... .......... .......... .......... .......... .......... ..........
DBP_1621_Brazil-BA        .......... .......... .......... .......... .......... .......... .......... .......... .......... ..........
DBP_1627_Brazil-BA        .......... .......... .......... .......... .......... .......... .......... .......... .......... ..........
DBP_1641_Brazil-BA        .......... .......... .......... .......... .......... .......... .......... .......... .......... ..........
DBP_1664_Brazil-BA        .......... .......... .......... .......... .......... .......... .......... .......... ......G... ..........
DBP_1741_Brazil-BA        .......... .......... .......... .......... .......... .......... .......... .......... .......... ..........
DBP_1747_Brazil-BA        .......... .......... .......... .......... .......... .......... .......... .......... .......... ..........
DBP_1748_Brazil-BA        .......... .......... .......... .......... .......... .......... .......... .......... .......... ..........
DBP_1777_Brazil-BA        .......... .......... .......... .......... .......... .......... .......... .......... .......... ..........
DBP_1789_Brazil-BA        .......... .......... .......... .......... .......... .......... .......... .......... ......G... ..........
DBP_1812_Brazil-BA        .......... .......... .......... .......... .......... .......... .......... .......... .......... ..........
DBP_1824_Brazil-BA        .......... .......... .......... .......... .......... .......... .......... .......... .......... ..........
DBP_1830_Brazil-BA        .......... .......... .......... .......... .......... .......... .......... .......... .......... ..........
DBP_1837_Brazil-BA        .......... .......... .......... .......... .......... .......... .......... .......... .......... ..........
DBP_1853_Brazil-BA        .......... .......... .......... .......... .......... .......... .......... .......... .......... ..........
DBP_1867_Brazil-BA        .......... .......... .......... .......... .......... .......... .......... .......... .......... ..........
DBP_1914_Brazil-BA        .......... .......... .......... .......... .......... .......... .......... .......... .......... ..........
DBP_1874_Brazil-BA        .......... .......... .......... .......... .......... .......... .......... .......... .......... ..........
DBP_2036_Brazil-BA        .......... .......... .......... .......... .......... .......... .......... .......... .......... ..........
DBP_2159_Brazil-BA        .......... .......... .......... .......... .......... .......... .......... .......... .......... ..........
DBP_2212_Brazil-BA        .......... .......... .......... .......... .......... .......... .......... .......... .......... ..........
DBP_2272_Brazil-BA        .......... .......... .......... .......... .......... .......... .......... .......... .......... ..........
DBP_2327_Brazil-BA        .......... .......... .......... .......... .......... .......... .......... .......... .......... ..........
DBP_2339_Brazil-BA        .......... .......... .......... .......... .......... .......... .......... .......... .......... ..........
DBP_2452_Brazil-BA        .......... .......... .......... .......... .......... .......... .......... .......... .......... ..........
DBP_2462_Brazil-BA        .......... .......... .......... .......... .......... .......... .......... .......... .......... ..........
DBP_2517_Brazil-BA        .......... .......... .......... .......... .......... .......... .......... .......... .......... ..........
DBP_2592_Brazil-BA        .......... .......... .......... .......... .......... .......... .......... .......... .......... ..........
DBP_2600_Brazil-BA        .......... .......... .......... .......... .......... .......... .......... .......... .......... ..........
DBP_2650_Brazil-BA        .......... .......... .......... .......... .......... .......... .......... .......... .......... ..........
DBP_2688_Brazil-BA        .......... .......... .......... .......... .......... .......... .......... .......... .......... ..........
DBP_2695_Brazil-BA        .......... .......... .......... .......... .......... .......... .......... .......... .......... ..........
DBP_2692_Brazil-BA        .......... .......... .......... .......... .......... .......... .......... .......... .......... ..........
DBP_2693_Brazil-BA        .......... .......... .......... .......... .......... .......... .......... .......... .......... ..........
```

```
DBP_2705_Brazil-BA    .......... .......... .......... .......... .......... .......... .......... .......... .......... ..........
DBP_2737_Brazil-BA    .......... .......... .......... .......... .......... .......... .......... .......... .......... ..........
DBP_2751_Brazil-BA    .......... .......... .......... .......... .......... .......... .......... .......... .......... ..........
DBP_2773_Brazil-BA    .......... .......... .......... .......... .......... .......... .......... .......... .......... ..........
DBP_3124_Brazil-BA    .......... .......... .......... .......... .......... .......... .......... .......... .......... ..........
DBP_3164_Brazil-BA    .......... .......... .......... .......... .......... .......... .......... .......... .......... ..........
DBP_3260_Brazil-BA    .......... .......... .......... .......... .......... .......... .......... .......... .......... ..........
DBP_3280_Brazil-BA    .......... .......... .......... .......... .......... .......... .......... .......... .......... ..........
DBP_3307_Brazil-BA    .......... .......... .......... .......... .......... .......... .......... .......... .......... ..........
DBP_3311_Brazil-BA    .......... .......... .......... .......... .......... .......... .......... .......... .......... ..........
DBP_3314_Brazil-BA    .......... .......... .......... .......... .......... .......... .......... .......... .......... ..........
DBP_3340_Brazil-BA    .......... .......... .......... .......... .......... .......... .......... .......... .......... ..........
DBP_3404_Brazil-BA    .......... .......... .......... .......... .......... .......... .......... .......... .......... ..........
DBP_3507_Brazil-BA    .......... .......... .......... .......... .......... .......... .......... .......... .......... ..........
DBP_3516_Brazil-BA    .......... .......... .......... .......... .......... .......... .......... .......... .......... ..........
DBP_3530_Brazil-BA    .......... .......... .......... .......... .......... .......... .......... .......... .......... ..........
DBP_3556_Brazil-BA    .......... .......... .......... .......... .......... .......... .......... .......... ......G... ..........
DBP_3560_Brazil-BA    .......... .......... .......... .......... .......... .......... .......... .......... .......... ..........
DBP_3569_Brazil-BA    .......... .......... .......... .......... .......... .......... .......... .......... .......... ..........
DBP_3601_Brazil-BA    .......... .......... .......... .......... .......... .......... .......... .......... .......... ..........
DBP_3617_Brazil-BA    .......... .......... .......... .......... .......... .......... .......... .......... .......... ..........
DBP_3661_Brazil-BA    .......... .......... .......... .......... .......... .......... .......... .......... .......... ..........
DBP_3707_Brazil-BA    .......... .......... .......... .......... .......... .......... .......... .......... ......G... ..........
DBP_3814_Brazil-BA    .......... .......... .......... .......... .......... .......... .......... .......... .......... ..........
DBP_3842_Brazil-BA    .......... .......... .......... .......... .......... .......... .......... .......... .......... ..........
DBP_3876_Brazil-BA    .......... .......... .......... .......... .......... .......... .......... .......... .......... ..........
DBP_3880_Brazil-BA    .......... .......... .......... .......... .......... .......... .......... .......... .......... ..........
DBP_3908_Brazil-BA    .......... .......... .......... .......... .......... .......... .......... .......... .......... ..........
DBP_3913_Brazil-BA    .......... .......... .......... .......... .......... .......... .......... .......... .......... ..........
DBP_3914_Brazil-BA    .......... .......... .......... .......... .......... .......... .......... .......... .......... ..........
DBP_3937_Brazil-BA    .......... .......... .......... .......... .......... .......... .......... .......... .......... ..........
DBP_3974_Brazil-BA    .......... .......... .......... .......... .......... .......... .......... .......... .......... ..........
DBP_3976_Brazil-BA    .......... .......... .......... .......... .......... .......... .......... .......... .......... ..........
DBP_3998_Brazil-BA    .......... .......... .......... .......... .......... .......... .......... .......... .......... ..........
DBP_4030_Brazil-BA    .......... .......... .......... .......... .......... .......... .......... .......... .......... ..........
DBP_4071_Brazil-BA    .......... .......... .......... .......... .......... .......... .......... .......... .......... ..........
DBP_4109_Brazil-BA    .......... .......... .......... .......... .......... .......... .......... .......... .......... ..........
DBP_4135_Brazil-BA    .......... .......... .......... .......... .......... .......... .......... .......... .......... ..........
DBP_1274_Brazil-AF    .......... .......... .......... .......... .......... .......... .......... .......... .......... ..........
DBP_1347_Brazil-AF    .......... .......... .......... .......... .......... .......... .......... .......... .......... ..........
DBP_1436_Brazil-AF    .......... .......... .......... .......... .......... .......... .......... .......... ......G... ..........
DBP_1737_Brazil-AF    .......... .......... .......... .......... .......... .......... .......... .......... .......... ..........
DBP_1652_Brazil-AF    .......... .......... .......... .......... .......... .......... .......... .......... .......... ..........
DBP_1745_Brazil-AF    .......... .......... .......... .......... .......... .......... .......... .......... .......... ..........
DBP_1856_Brazil-AF    .......... .......... .......... .......... .......... .......... .......... .......... .......... ..........
DBP_1906_Brazil-AF    .......... .......... .......... .......... .......... .......... .......... .......... .......... ..........
DBP_1948_Brazil-AF    .......... .......... .......... .......... .......... .......... .......... .......... .......... ..........
DBP_1960_Brazil-AF    .......... .......... .......... .......... .......... .......... .......... .......... .......... ..........
DBP_1961_Brazil-AF    .......... .......... .......... .......... .......... .......... .......... .......... .......... ..........
DBP_2728_Brazil-AF    .......... .......... .......... .......... .......... .......... .......... .......... .......... ..........
DBP_2734_Brazil-AF    .......... .......... .......... .......... .......... .......... .......... .......... .......... ..........
```

```
DBP_2736_Brazil-AF .......... .......... .......... .......... .......... .......... .......... .......... .......... ..........
DBP_2747_Brazil-AF .......... .......... .......... .......... .......... .......... .......... .......... .......... ..........
DBP_2752_Brazil-AF .......... .......... .......... .......... .......... .......... .......... .......... .......... ..........
DBP_2762_Brazil-AF .......... .......... .......... .......... .......... .......... .......... .......... .......... ..........
DBP_2765_Brazil-AF .......... .......... .......... .......... .......... .......... .......... .......... .......... ..........
DBP_2769_Brazil-AF .......... .......... .......... .......... .......... .......... .......... .......... .......... ..........
DBP_2770_Brazil-AF .......... .......... .......... .......... .......... .......... .......... .......... .......... ..........
DBP_2775_Brazil-AF .......... .......... .......... .......... .......... .......... .......... .......... .......... ..........
DBP_2779_Brazil-AF .......... .......... .......... .......... .......... .......... .......... .......... .......... ..........
DBP_2782_Brazil-AF .......... .......... .......... .......... .......... .......... .......... .......... .......... ..........
DBP_2792_Brazil-AF .......... .......... .......... .......... .......... .......... .......... .......... .......... ..........
DBP_2793_Brazil-AF .......... .......... .......... .......... .......... .......... .......... .......... .......... ..........
DBP_2816_Brazil-AF .......... .......... .......... .......... .......... .......... .......... .......... .......... ..........
DBP_2840_Brazil-AF .......... .......... .......... .......... .......... .......... .......... .......... .......... ..........
DBP_2859_Brazil-AF .......... .......... .......... .......... .......... .......... .......... .......... .......... ..........
DBP_2900_Brazil-AF .......... .......... .......... .......... .......... .......... .......... .......... .......... ..........
DBP_2916_Brazil-AF .......... .......... .......... .......... .......... .......... .......... .......... .......... ..........
DBP_2923_Brazil-AF .......... .......... .......... .......... .......... .......... .......... .......... .......... ..........
DBP_2980_Brazil-AF .......... .......... .......... .......... .......... .......... .......... .......... .......... ..........
DBP_2982_Brazil-AF .......... .......... .......... .......... .......... .......... .......... .......... .......... ..........
DBP_2983_Brazil-AF .......... .......... .......... .......... .......... .......... .......... .......... .......... ..........
DBP_3071_Brazil-AF .......... .......... .......... .......... .......... .......... .......... .......... .......... ..........
DBP_3079_Brazil-AF .......... .......... .......... .......... .......... .......... .......... .......... .......... ..........
DBP_3084_Brazil-AF .......... .......... .......... .......... .......... .......... .......... .......... .......... ..........
DBP_3089_Brazil-AF .......... .......... .......... .......... .......... .......... .......... .......... .......... ..........
DBP_3157_Brazil-AF .......... .......... .......... .......... .......... .......... .......... .......... .......... ..........
DBP_3276_Brazil-AF .......... .......... .......... .......... .......... .......... .......... .......... .......... ..........
DBP_3277_Brazil-AF .......... .......... .......... .......... .......... .......... .......... .......... .......... ..........
DBP_3278_Brazil-AF .......... .......... .......... .......... .......... .......... .......... .......... .......... ..........
DBP_3284_Brazil-AF .......... .......... .......... .......... .......... .......... .......... .......... .......... ..........
DBP_3296_Brazil-AF .......... .......... .......... .......... .......... .......... .......... .......... .......... ..........
DBP_3300_Brazil-AF .......... .......... .......... .......... .......... .......... .......... .......... .......... ..........
DBP_3310_Brazil-AF .......... .......... .......... .......... .......... .......... .......... .......... .......... ..........
DBP_3315_Brazil-AF .......... .......... .......... .......... .......... .......... .......... .......... .......... ..........
DBP_3325_Brazil-AF .......... .......... .......... .......... .......... .......... .......... .......... .......... ..........
DBP_3334_Brazil-AF .......... .......... .......... .......... .......... .......... .......... .......... .......... ..........
DBP_3371_Brazil-AF .......... .......... .......... .......... .......... .......... .......... .......... .......... ..........
DBP_3376_Brazil-AF .......... .......... .......... .......... .......... .......... .......... .......... .......... ..........
DBP_3379_Brazil-AF .......... .......... .......... .......... .......... .......... .......... .......... .......... ..........
DBP_3406_Brazil-AF .......... .......... .......... .......... .......... .......... .......... .......... .......... ..........
DBP_3612_Brazil-AF .......... .......... .......... .......... .......... .......... .......... .......... .......... ..........
DBP_3613_Brazil-AF .......... .......... .......... .......... .......... .......... .......... .......... .......... ..........
DBP_3614_Brazil-AF .......... .......... .......... .......... .......... .......... .......... .......... .......... ..........
DBP_3616_Brazil-AF .......... .......... .......... .......... .......... .......... .......... .......... .......... ..........
DBP_3630_Brazil-AF .......... .......... .......... .......... .......... .......... .......... .......... .......... ..........
DBP_3633_Brazil-AF .......... .......... .......... .......... .......... .......... .......... .......... .......... ..........
DBP_3643_Brazil-AF .......... .......... .......... .......... .......... .......... .......... .......... .......... ..........
DBP_3659_Brazil-AF .......... .......... .......... .......... .......... .......... .......... .......... .......... ..........
DBP_3698_Brazil-AF .......... .......... .......... .......... .......... .......... .......... .......... .......... ..........
DBP_3705_Brazil-AF .......... .......... .......... .......... .......... .......... .......... .......... ......G... ..........
DBP_3722_Brazil-AF .......... .......... .......... .......... .......... .......... .......... .......... .......... ..........
```

```
DBP_3735_Brazil-AF       .......... .......... .......... .......... .......... .......... .......... .......... .......... ..........
DBP_3736_Brazil-AF       .......... .......... .......... .......... .......... .......... .......... .......... .......... ..........
DBP_3739_Brazil-AF       .......... .......... .......... .......... .......... .......... .......... .......... .......... ..........
DBP_3746_Brazil-AF       .......... .......... .......... .......... .......... .......... .......... .......... .......... ..........
DBP_3750_Brazil-AF       .......... .......... .......... .......... .......... .......... .......... .......... .......... ..........
DBP_3916_Brazil-AF       .......... .......... .......... .......... .......... .......... .......... .......... .......... ..........
DBP_3931_Brazil-AF       .......... .......... .......... .......... .......... .......... .......... .......... .......... ..........
DBP_3962_Brazil-AF       .......... .......... .......... .......... .......... .......... .......... .......... .......... ..........
DBP_4083_Brazil-AF       .......... .......... .......... .......... .......... .......... .......... .......... .......... ..........
DBP_4096_Brazil-AF       .......... .......... .......... .......... .......... .......... .......... .......... .......... ..........
DBP_4097_Brazil-AF       .......... .......... .......... .......... .......... .......... .......... .......... .......... ..........
DBP_4112_Brazil-AF       .......... .......... .......... .......... .......... .......... .......... .......... .......... ..........
DBP_4118_Brazil-AF       .......... .......... .......... .......... .......... .......... .......... .......... .......... ..........
DBP_4131_Brazil-AF       .......... .......... .......... .......... .......... .......... .......... .......... .......... ..........
DBP_4161_Brazil-AF       .......... .......... .......... .......... .......... .......... .......... .......... .......... ..........

                                610        620        630        640        650        660        670
                         ....|....| ....|....| ....|....| ....|....| ....|....| ....|....| ....|....| ....|.
PVX_110810_|_Salvador 1  AAAAAAAAAT CAATGGGATG TTCTGTCAAA TAAATTCATA AGTGTAAAAA ACGCAGAAAA GGTTCAGACG GCAGGT
DBP_07_Brazil-BA         .......... .......... .......... ........A. .......... .......... .......... ......
DBP_09_Brazil-BA         .......... .......... .......... ........A. .......... .......... .......... ......
DBP_10_Brazil-BA         .......... .......... .......... .......... .......... .......... .......... ......
DBP_11_Brazil-BA         .......... .......... .......... .......... .......... .......... .......... ......
DBP_12_Brazil-BA         .......... .......... .......... .......... .......... .......... .......... ......
DBP_13_Brazil-BA         .......... .......... .......... ........A. .......... .......... .......... ......
DBP_14_Brazil-BA         .......... .......... .......... .......... .......... .......... .......... ......
DBP_15_Brazil-BA         .......... .......... .......... .......... .......... .......... .......... ......
DBP_16_Brazil-BA         .......... .......... .......... ........A. .......... .......... .......... ......
DBP_17_Brazil-BA         .......... .......... .......... .......... .......... .......... .......... ......
DBP_18_Brazil-BA         .......... .......... .......... ........A. .......... .......... .......... ......
DBP_20_Brazil-BA         .......... .......... .......... ........A. .......... .......... .......... ......
DBP_21_Brazil-BA         .......... .......... .......... .......... .......... .......... .......... ......
DBP_22_Brazil-BA         .......... .......... .......... .......... .......... .......... .......... ......
DBP_24_Brazil-BA         .......... .......... .......... ........A. .......... .......... .......... ......
DBP_26_Brazil-BA         .......... .......... .......... ........A. .......... .......... .......... ......
DBP_27_Brazil-BA         .......... .......... .......... .......... .......... .......... .......... ......
DBP_28_Brazil-BA         .......... .......... .......... .......... .......... .......... .......... ......
DBP_29_Brazil-BA         .......... .......... .......... ........A. .......... .......... .......... ......
DBP_31_Brazil-BA         .......... .......... .......... .......... .......... .......... .......... ......
DBP_32_Brazil-BA         .......... .......... .......... ........A. .......... .......... .......... ......
DBP_33_Brazil-BA         .......... .......... .......... ........A. .......... .......... .......... ......
DBP_35_Brazil-BA         .......... .......... .......... ........A. .......... .......... .......... ......
DBP_36_Brazil-BA         .......... .......... .......... ........A. .......... .......... .......... ......
DBP_37_Brazil-BA         .......... .......... .......... .......... .......... .......... .......... ......
DBP_38_Brazil-BA         .......... .......... .......... ........A. .......... .......... .......... ......
DBP_39_Brazil-BA         .......... .......... .......... ........A. .......... .......... .......... ......
DBP_40_Brazil-BA         .......... .......... .......... ........A. .......... .......... .......... ......
DBP_41_Brazil-BA         .......... .......... .......... ........A. .......... .......... .......... ......
DBP_42_Brazil-BA         .......... .......... .......... ........A. .......... .......... .......... ......
DBP_43_Brazil-BA         .......... .......... .......... .......... .......... .......... .......... ......
DBP_44_Brazil-BA         .......... .......... .......... .......... .......... .......... .......... ......
```

```
DBP_45_Brazil-BA      .......... .......... .......... ........A. .......... .......... .......... ......
DBP_46_Brazil-BA      .......... .......... .......... ........A. .......... .......... .......... ......
DBP_47_Brazil-BA      .......... .......... .......... .......... .......... .......... .......... ......
DBP_48_Brazil-BA      .......... .......... .......... ........A. .......... .......... .......... ......
DBP_49_Brazil-BA      .......... .......... .......... ........A. .......... .......... .......... ......
DBP_51_Brazil-BA      .......... .......... .......... ........A. .......... .......... .......... ......
DBP_53_Brazil-BA      .......... .......... .......... ........A. .......... .......... .......... ......
DBP_54_Brazil-BA      .......... .......... .......... ........A. .......... .......... .......... ......
DBP_55_Brazil-BA      .......... .......... .......... .......... .......... .......... .......... ......
DBP_56_Brazil-BA      .......... .......... .......... .......... .......... .......... .......... ......
DBP_57_Brazil-BA      .......... .......... .......... ........A. .......... .......... .......... ......
DBP_58_Brazil-BA      .......... .......... .......... .......... .......... .......... .......... ......
DBP_59_Brazil-BA      .......... .......... .......... ........A. .......... .......... .......... ......
DBP_1161_Brazil-BA    .......... .......... .......... ........A. .......... .......... .......... ......
DBP_1182_Brazil-BA    .......... .......... .......... .......... .......... .......... .......... ......
DBP_1190_Brazil-BA    .......... .......... .......... .......... .......... .......... .......... ......
DBP_1200_Brazil-BA    .......... .......... .......... ........A. .......... .......... .......... ......
DBP_1209_Brazil-BA    .......... .......... .......... .......... .......... .......... .......... ......
DBP_1272_Brazil-BA    .......... .......... .......... .......... .......... .......... .......... ......
DBP_1295_Brazil-BA    .......... .......... .......... .......... .......... .......... .......... ......
DBP_1320_Brazil-BA    .......... .......... .......... .......... .......... .......... .......... ......
DBP_1327_Brazil-BA    .......... .......... .......... .......... .......... .......... .......... ......
DBP_1335_Brazil-BA    .......... .......... .......... ........A. .......... .......... .......... ......
DBP_1340_Brazil-BA    .......... .......... .......... ........A. .......... .......... .......... ......
DBP_1346_Brazil-BA    .......... .......... .......... .......... .......... .......... .......... ......
DBP_1382_Brazil-BA    .......... .......... .......... .......... .......... .......... .......... ......
DBP_1428_Brazil-BA    .......... .......... .......... ........A. .......... .......... .......... ......
DBP_1447_Brazil-BA    .......... .......... .......... ........A. .......... .......... .......... ......
DBP_1469_Brazil-BA    .......... .......... .......... ........A. .......... .......... .......... ......
DBP_1473_Brazil-BA    .......... .......... .......... .......... .......... .......... .......... ......
DBP_1475_Brazil-BA    .......... .......... .......... ........A. .......... .......... .......... ......
DBP_1493_Brazil-BA    .......... .......... .......... .......... .......... .......... .......... ......
DBP_1532_Brazil-BA    .......... .......... .......... ........A. .......... .......... .......... ......
DBP_1540_Brazil-BA    .......... .......... .......... .......... .......... .......... .......... ......
DBP_1621_Brazil-BA    .......... .......... .......... ........A. .......... .......... .......... ......
DBP_1627_Brazil-BA    .......... .......... .......... ........A. .......... .......... .......... ......
DBP_1641_Brazil-BA    .......... .......... .......... ........A. .......... .......... .......... ......
DBP_1664_Brazil-BA    .......... .......... .......... .......... .......... .......... .......... ......
DBP_1741_Brazil-BA    .......... .......... .......... ........A. .......... .......... .......... ......
DBP_1747_Brazil-BA    .......... .......... .......... ........A. .......... .......... .......... ......
DBP_1748_Brazil-BA    .......... .......... .......... ........A. .......... .......... .......... ......
DBP_1777_Brazil-BA    .......... .......... .......... .......... .......... .......... .......... ......
DBP_1789_Brazil-BA    .......... .......... .......... .......... .......... .......... .......... ......
DBP_1812_Brazil-BA    .......... .......... .......... ........A. .......... .......... .......... ......
DBP_1824_Brazil-BA    .......... .......... .......... ........A. .......... .......... .......... ......
DBP_1830_Brazil-BA    .......... .......... .......... ........A. .......... .......... .......... ......
DBP_1837_Brazil-BA    .......... .......... .......... ........A. .......... .......... .......... ......
DBP_1853_Brazil-BA    .......... .......... .......... ........A. .......... .......... .......... ......
DBP_1867_Brazil-BA    .......... .......... .......... .......... .......... .......... .......... ......
DBP_1914_Brazil-BA    .......... .......... .......... .......... .......... .......... .......... ......
DBP_1874_Brazil-BA    .......... .......... .......... .......... .......... .......... .......... ......
```

```
DBP_2036_Brazil-BA    .......... .......... .......... .......... .......... .......... .......... ......
DBP_2159_Brazil-BA    .......... .......... .......... .......... .......... .......... .......... ......
DBP_2212_Brazil-BA    .......... .......... .......... .......... .......... .......... .......... ......
DBP_2272_Brazil-BA    .......... .......... .......... .......... .......... .......... .......... ......
DBP_2327_Brazil-BA    .......... .......... .......... ........A. .......... .......... .......... ......
DBP_2339_Brazil-BA    .......... .......... .......... .......... .......... .......... .......... ......
DBP_2452_Brazil-BA    .......... .......... .......... .......... .......... .......... .......... ......
DBP_2462_Brazil-BA    .......... .......... .......... .......... .......... .......... .......... ......
DBP_2517_Brazil-BA    .......... .......... .......... .......... .......... .......... .......... ......
DBP_2592_Brazil-BA    .......... .......... .......... .......... .......... .......... .......... ......
DBP_2600_Brazil-BA    .......... .......... .......... .......... .......... .......... .......... ......
DBP_2650_Brazil-BA    .......... .......... .......... .......... .......... .......... .......... ......
DBP_2688_Brazil-BA    .......... .......... .......... .......... .......... .......... .......... ......
DBP_2695_Brazil-BA    .......... .......... .......... .......... .......... .......... .......... ......
DBP_2692_Brazil-BA    .......... .......... .......... .......... .......... .......... .......... ......
DBP_2693_Brazil-BA    .......... .......... .......... .......... .......... .......... .......... ......
DBP_2705_Brazil-BA    .......... .......... .......... .......... .......... .......... .......... ......
DBP_2737_Brazil-BA    .......... .......... .......... .......... .......... .......... .......... ......
DBP_2751_Brazil-BA    .......... .......... .......... .......... .......... .......... .......... ......
DBP_2773_Brazil-BA    .......... .......... .......... .......... .......... .......... .......... ......
DBP_3124_Brazil-BA    .......... .......... .......... ........A. .......... .......... .......... ......
DBP_3164_Brazil-BA    .......... .......... .......... ........A. .......... .......... .......... ......
DBP_3260_Brazil-BA    .......... .......... .......... .......... .......... .......... .......... ......
DBP_3280_Brazil-BA    .......... .......... .......... ........A. .......... .......... .......... ......
DBP_3307_Brazil-BA    .......... .......... .......... .......... .......... .......... .......... ......
DBP_3311_Brazil-BA    .......... .......... .......... ........A. .......... .......... .......... ......
DBP_3314_Brazil-BA    .......... .......... .......... ........A. .......... .......... .......... ......
DBP_3340_Brazil-BA    .......... .......... .......... ........A. .......... .......... .......... ......
DBP_3404_Brazil-BA    .......... .......... .......... ........A. .......... .......... .......... ......
DBP_3507_Brazil-BA    .......... .......... .......... .......... .......... .......... .......... ......
DBP_3516_Brazil-BA    .......... .......... .......... ........A. .......... .......... .......... ......
DBP_3530_Brazil-BA    .......... .......... .......... .......... .......... .......... .......... ......
DBP_3556_Brazil-BA    .......... .......... .......... .......... .......... .......... .......... ......
DBP_3560_Brazil-BA    .......... .......... .......... .......... .......... .......... .......... ......
DBP_3569_Brazil-BA    .......... .......... .......... ........A. .......... .......... .......... ......
DBP_3601_Brazil-BA    .......... .......... .......... .......... .......... .......... .......... ......
DBP_3617_Brazil-BA    .......... .......... .......... .......... .......... .......... .......... ......
DBP_3661_Brazil-BA    .......... .......... .......... .......... .......... .......... .......... ......
DBP_3707_Brazil-BA    .......... .......... .......... .......... .......... .......... .......... ......
DBP_3814_Brazil-BA    .......... .......... .......... .......... .......... .......... .......... ......
DBP_3842_Brazil-BA    .......... .......... .......... .......... .......... .......... .......... ......
DBP_3876_Brazil-BA    .......... .......... .......... .......... .......... .......... .......... ......
DBP_3880_Brazil-BA    .......... .......... .......... .......... .......... .......... .......... ......
DBP_3908_Brazil-BA    .......... .......... .......... ........A. .......... .......... .......... ......
DBP_3913_Brazil-BA    .......... .......... .......... ........A. .......... .......... .......... ......
DBP_3914_Brazil-BA    .......... .......... .......... .......... .......... .......... .......... ......
DBP_3937_Brazil-BA    .......... .......... .......... .......... .......... .......... .......... ......
DBP_3974_Brazil-BA    .......... .......... .......... ........A. .......... .......... .......... ......
DBP_3976_Brazil-BA    .......... .......... .......... .......... .......... .......... .......... ......
DBP_3998_Brazil-BA    .......... .......... .......... ........A. .......... .......... .......... ......
DBP_4030_Brazil-BA    .......... .......... .......... .......... .......... .......... .......... ......
```

```
DBP_4071_Brazil-BA        .......... .......... .......... ........A. .......... .......... .......... ......
DBP_4109_Brazil-BA        .......... .......... .......... .......... .......... .......... .......... ......
DBP_4135_Brazil-BA        .......... .......... .......... .......... .......... .......... .......... ......
DBP_1274_Brazil-AF        .......... .......... .......... ........A. .......... .......... .......... ......
DBP_1347_Brazil-AF        .......... .......... .......... .......... .......... .......... .......... ......
DBP_1436_Brazil-AF        .......... .......... .......... .......... .......... .......... .......... ......
DBP_1737_Brazil-AF        .......... .......... .......... ........A. .......... .......... .......... ......
DBP_1652_Brazil-AF        .......... .......... .......... ........A. .......... .......... .......... ......
DBP_1745_Brazil-AF        .......... .......... .......... ........A. .......... .......... .......... ......
DBP_1856_Brazil-AF        .......... .......... .......... .......... .......... .......... .......... ......
DBP_1906_Brazil-AF        .......... .......... .......... .......... .......... .......... .......... ......
DBP_1948_Brazil-AF        .......... .......... .......... .......... .......... .......... .......... ......
DBP_1960_Brazil-AF        .......... .......... .......... .......... .......... .......... .......... ......
DBP_1961_Brazil-AF        .......... .......... .......... ........A. .......... .......... .......... ......
DBP_2728_Brazil-AF        .......... .......... .......... .......... .......... .......... .......... ......
DBP_2734_Brazil-AF        .......... .......... .......... .......... .......... .......... .......... ......
DBP_2736_Brazil-AF        .......... .......... .......... .......... .......... .......... .......... ......
DBP_2747_Brazil-AF        .......... .......... .......... .......... .......... .......... .......... ......
DBP_2752_Brazil-AF        .......... .......... .......... .......... .......... .......... .......... ......
DBP_2762_Brazil-AF        .......... .......... .......... .......... .......... .......... .......... ......
DBP_2765_Brazil-AF        .......... .......... .......... .......... .......... .......... .......... ......
DBP_2769_Brazil-AF        .......... .......... .......... .......... .......... .......... .......... ......
DBP_2770_Brazil-AF        .......... .......... .......... .......... .......... .......... .......... ......
DBP_2775_Brazil-AF        .......... .......... .......... .......... .......... .......... .......... ......
DBP_2779_Brazil-AF        .......... .......... .......... .......... .......... .......... .......... ......
DBP_2782_Brazil-AF        .......... .......... .......... .......... .......... .......... .......... ......
DBP_2792_Brazil-AF        .......... .......... .......... .......... .......... .......... .......... ......
DBP_2793_Brazil-AF        .......... .......... .......... .......... .......... .......... .......... ......
DBP_2816_Brazil-AF        .......... .......... .......... .......... .......... .......... .......... ......
DBP_2840_Brazil-AF        .......... .......... .......... .......... .......... .......... .......... ......
DBP_2859_Brazil-AF        .......... .......... .......... .......... .......... .......... .......... ......
DBP_2900_Brazil-AF        .......... .......... .......... .......... .......... .......... .......... ......
DBP_2916_Brazil-AF        .......... .......... .......... .......... .......... .......... .......... ......
DBP_2923_Brazil-AF        .......... .......... .......... .......... .......... .......... .......... ......
DBP_2980_Brazil-AF        .......... .......... .......... .......... .......... .......... .......... ......
DBP_2982_Brazil-AF        .......... .......... .......... .......... .......... .......... .......... ......
DBP_2983_Brazil-AF        .......... .......... .......... .......... .......... .......... .......... ......
DBP_3071_Brazil-AF        .......... .......... .......... .......... .......... .......... .......... ......
DBP_3079_Brazil-AF        .......... .......... .......... .......... .......... .......... .......... ......
DBP_3084_Brazil-AF        .......... .......... .......... .......... .......... .......... .......... ......
DBP_3089_Brazil-AF        .......... .......... .......... .......... .......... .......... .......... ......
DBP_3157_Brazil-AF        .......... .......... .......... .......... .......... .......... .......... ......
DBP_3276_Brazil-AF        .......... .......... .......... .......... .......... .......... .......... ......
DBP_3277_Brazil-AF        .......... .......... .......... .......... .......... .......... .......... ......
DBP_3278_Brazil-AF        .......... .......... .......... .......... .......... .......... .......... ......
DBP_3284_Brazil-AF        .......... .......... .......... .......... .......... .......... .......... ......
DBP_3296_Brazil-AF        .......... .......... .......... ........A. .......... .......... .......... ......
DBP_3300_Brazil-AF        .......... .......... .......... .......... .......... .......... .......... ......
DBP_3310_Brazil-AF        .......... .......... .......... .......... .......... .......... .......... ......
DBP_3315_Brazil-AF        .......... .......... .......... .......... .......... .......... .......... ......
DBP_3325_Brazil-AF        .......... .......... .......... .......... .......... .......... .......... ......
```

```
DBP_3334_Brazil-AF    .......... .......... .......... .......... .......... .......... .......... ......
DBP_3371_Brazil-AF    .......... .......... .......... .......... .......... .......... .......... ......
DBP_3376_Brazil-AF    .......... .......... .......... ........A. .......... .......... .......... ......
DBP_3379_Brazil-AF    .......... .......... .......... ........A. .......... .......... .......... ......
DBP_3406_Brazil-AF    .......... .......... .......... .......... .......... .......... .......... ......
DBP_3612_Brazil-AF    .......... .......... .......... .......... .......... .......... .......... ......
DBP_3613_Brazil-AF    .......... .......... .......... .......... .......... .......... .......... ......
DBP_3614_Brazil-AF    .......... .......... .......... ........A. .......... .......... .......... ......
DBP_3616_Brazil-AF    .......... .......... .......... .......... .......... .......... .......... ......
DBP_3630_Brazil-AF    .......... .......... .......... .......... .......... .......... .......... ......
DBP_3633_Brazil-AF    .......... .......... .......... .......... .......... .......... .......... ......
DBP_3643_Brazil-AF    .......... .......... .......... .......... .......... .......... .......... ......
DBP_3659_Brazil-AF    .......... .......... .......... .......... .......... .......... .......... ......
DBP_3698_Brazil-AF    .......... .......... .......... .......... .......... .......... .......... ......
DBP_3705_Brazil-AF    .......... .......... .......... .......... .......... .......... .......... ......
DBP_3722_Brazil-AF    .......... .......... .......... .......... .......... .......... .......... ......
DBP_3735_Brazil-AF    .......... .......... .......... .......... .......... .......... .......... ......
DBP_3736_Brazil-AF    .......... .......... .......... .......... .......... .......... .......... ......
DBP_3739_Brazil-AF    .......... .......... .......... .......... .......... .......... .......... ......
DBP_3746_Brazil-AF    .......... .......... .......... .......... .......... .......... .......... ......
DBP_3750_Brazil-AF    .......... .......... .......... .......... .......... .......... .......... ......
DBP_3916_Brazil-AF    .......... .......... .......... ........A. .......... .......... .......... ......
DBP_3931_Brazil-AF    .......... .......... .......... .......... .......... .......... .......... ......
DBP_3962_Brazil-AF    .......... .......... .......... .......... .......... .......... .......... ......
DBP_4083_Brazil-AF    .......... .......... .......... .......... .......... .......... .......... ......
DBP_4096_Brazil-AF    .......... .......... .......... ........A. .......... .......... .......... ......
DBP_4097_Brazil-AF    .......... .......... .......... .......... .......... .......... .......... ......
DBP_4112_Brazil-AF    .......... .......... .......... ........A. .......... .......... .......... ......
DBP_4118_Brazil-AF    .......... .......... .......... .......... .......... .......... .......... ......
DBP_4131_Brazil-AF    .......... .......... .......... ........A. .......... .......... .......... ......
DBP_4161_Brazil-AF    .......... .......... .......... ........A. .......... .......... .......... ......
```
