# Supplementary material for: Extensive genetic diversity of Plasmodium vivax dbp-II in Rio de Janeiro Atlantic Forest and Brazilian Amazon Basin: evidence of positive selection
Source: Malar J. 2020 Feb 19;19:81. doi: 10.1186/s12936-020-03159-y (PMC7031913; doi:10.1186/s12936-020-03159-y)
Supplement: Supplementary file 3 — Additional file 3.Pvdbp-II haplotypes. Identification of 90 haplotypes and their respective nucleotide sequence compared to Salvador 1 reference sequence (PVX_110810). [file 12936_2020_3159_MOESM3_ESM.pdf]

Identifier	Haplotypes	N of polymorphic sites
DB01	YSIFKNRGKTHSRNNIIWQQI	11
DB02	NRILKNRGEKHSTKNILWQQK	3
DB03	NRILENRGEQRSTKKIIRQQI	6
DB04	NRILENRGEQRSTKKIIRQQK	7
DB05	NRILKNRGEKHSTKNILWQQI	2
DB06	NSILENRGKNRSTKKIIRQQK	9
DB07	YSIFKNRGKNHSRNNLIWQQI	12
DB08	NRILKNRGEKHSTKNILWQEI	3
DB09	NRILKNRDEKRSTKKIIRQQK	4
DB10	NRILENRGEKRSTKKMIRQQI	6
DB11	YSSFKNRGQNHSRNNLIWQQI	13
DB12	YRILENRGEQRSTKKIIRQQK	8
DB13	YRILENRGEQRSTKKIIRPQK	9
DB14	NRILENRGEKHSTKNILWQQI	3
DB15	NSILKNRGKNHTRKNIIWQQI	9
DB16	NRILENRGEQRSTKKIIRPQK	8
DB17	YSSFNNRGQNHSRNNLIWQQI	14
DB18	YSILKNRGTNHSRNNIIWQQI	10
DB19	NSILENRGKNRSTKKIIRQQI	8
DB20	NRILKNRGEKRSTKNILWQQI	1
DB21	NRILENRGEQRSTKNIIRQQK	6
DB22	YRILENRGEKRSTKKMIRQQK	8
DB23	YSSFNNRGQNHSRNNRIWQQK	15
DB24	YSSFNNRGQNHSRNNRIWQQI	14
DB25	YSIFKNRGQNHSRNNIIWQQK	12
DB26	YSIFKNRGKTHSRNNIIWPQI	12
DB27	YSIFKNRGKNHSRNNIIWQQI	11
DB28	NSILKNRGKNHTRKNIIWQQK	9
DB29	NSILKNRGKNHSTKNILWQQI	6
DB30	NSILENRGKNRSTKKIIRPQK	10
DB31	NRILKNRGEKRSTKNILRQQK	3
DB32	NRIFKDRGKNHSTKKIIRQQI	10
DB33	YSSFNNRGQNHSRNNLIWPQI	15
DB34	YSSFKNRGQNHSRNNRIWQQI	13
DB35	YSSFKNRGQNHSRNNRIWPQK	15
DB36	YSSFKNRGQNHSRNNLIWPQI	14
DB37	YSILKNRGKNRSTKNILWPQK	7
DB38	YSILKNRGKNHSRNNLIWQQI	11
DB39	YSILKNRGKNHSRNNIIWPQI	11
DB40	YSILKNRGEKRSTKNILRPQI	5
DB41	YSILENRGKNRSTKKIIRQQI	8
DB42	YSILENRGEQRSTKKIIWQQK	8

DB43	YSIFNNRGQNHSRNNIIWQQI	12
DB44	YSIFKNRGQNHSRNNLIWQQI	12
DB45	YSIFKNRGQNHSRNNIIWQQI	11
DB46	YSIFKNRGKTHSRNNIIWQQK	12
DB47	YSIFKNRGKNRSRNNLIWQQI	9
DB48	YRILKNRGEKHSTKNILWQQK	4
DB49	YRILENRGEKRSTKKMIRQQI	7
DB50	YRIFKNRGKTHSRNNIIWQQI	10
DB51	YRIFKNRGKQHSRKNIIWQQK	9
DB52	NSSFNNRGQNHSRNNLIWQQI	14
DB53	NSILKNRGKTHSRNNIIWQQI	8
DB54	NSILKNRGKNRSTKKIIRQQI	7
DB55	NSILKNRGKNHTRKNIIWQEI	10
DB56	NSILKNRGKNHSTKNMLWQQI	6
DB57	NSILKNRGEKHSTKNILWQQI	3
DB58	NSILENRGEKHSTKNIIRQQI	6
DB59	NSIFKNRGKTHSRNNIIWQQI	9
DB60	NRSLENRGEQRSTNKIIRQQI	8
DB61	NRILKNRGKNRSTKNIIWQQI	4
DB62	NRILKNRGKNHTRKNIIWQQI	8
DB63	NRILKNRGKKRSTKNILWQQI	2
DB64	NRILKNRGEQRSTKNILWQQI	2
DB65	NRILKNRGEQRSTKKIIRPQK	7
DB66	NRILKNRGEKRSTKNIIWQQI	2
DB67	NRILKNRGEKRSTKKMIWQQI	4
DB68	NRILKNRGEKRSTKKMIRQQI	5
DB69	NRILKNRGEKRSTKKIIRQQI	4
DB70	NRILKNRGEKHSTKNILWPQK	4
DB71	NRILKNRDEQRSTKKIIRQQI	4
DB72	NRILKNRDENRSTKNIIRQQI	3
DB73	NRILKNRDEKRSTKNILWQQK	1
DB74	NRILKNRDEKRSTKKIIRPQI	4
DB75	NRILKNRDEKHSTKNILWQQK	2
DB76	NRILKNRDEKHSTKNIIRQQI	3
DB77	NRILKDRDEQHSTKNILRQQI	4
DB78	NRILKDRDEKRSTKKIIRQQI	4
DB79	NRILENRGQNRSTKNIIRQQI	6
DB80	NRILENRGEQRSTNKIIRPQK	9
DB81	NRILENRGEQRSTKKILWQQI	4
DB82	NRILENRGEQRSTKKILRQQK	6
DB83	NRILENRGEKRSTKKMLRQQI	5
DB84	NRILENRGEKRSTKKIIRQQI	4
DB85	NRILENRGEKHSTKKMIRQQI	8
DB86	NRIFKNRGKKHSRNNLIWQQI	9

DB87	NRIFK**D**R**GKNH**STK**K**I**IR**QQK	8
DB88	NRI**F**K**DR**D**KQH**STK**K**I**I**WQQI	8
DB89	NRI**FE**N**RGKNH**STK**K**I**IR**QQI	10
DB90	NRI**FE**NR**G**E**Q**RSTK**K**I**IR**QQI	6

PvDBP-II gene haplotypes. Characters in bold represent amino acids resulting from non-synonymous (red) and synonymous (blue) substitutions observed in their respective nucleotide sequence compared to Salvador 1 (Sal-1) strain PvDBP-II gene sequence PVX_110810.
